# Impact of high fat diet on long non-coding RNAs and messenger RNAs expression in the aortas of ApoE(−/−) mice

**DOI:** 10.1038/srep34161

**Published:** 2016-10-04

**Authors:** Mei-hua Bao, Huai-qing Luo, Li-hua Chen, Liang Tang, Kui-fen Ma, Ju Xiang, Li-ping Dong, Jie Zeng, Guang-yi Li, Jian-ming Li

**Affiliations:** 1 Department of Anatomy, Histology and Embryology, Institute of of Neuroscience, Changsha Medical University, Changsha, 410219, China; 2 The Third Xiangya Hospital of Central South University, Changsha 410013, Hunan, PR China; 3 The First Affiliated Hospital, Zhejiang University, Hangzhou, Zhejiang, PR China; 4 Department of Neurology, Xiangya Hospital, Central South University, Changsha, Hunan 410008, China; 5 Department of Anatomy, Xiangya School of Medicine, Central South University, Changsha, Hunan 410013, China

## Abstract

Atherosclerosis is a chronic multifactorial inflammatory disease with high prevalence worldwide, and has become the leading cause of death. The present study was designed to investigate the impact of high-fat diet on ApoE(−/−) mice exhibiting atherosclerosis by detecting the genome-wide expression profile of lncRNAs and mRNAs. A total of 354 differentially expressed lncRNAs were identified (≥2.0 folds). Simultaneously, 357 differentially expressed mRNAs from the same chip were found. The expression differences of lncRNAs and mRNAs were consistent in both qPCR and microarray detection. Annotation results of the mRNAs which correlated with lncRNAs showed that the commonly related pathways were metabolism and inflammation. Hypergeometric distribution analysis indicated that the differentially expressed lncRNAs had been mostly regulated by transcription factors (TFs) such as Myod1, Rxra, Pparg, Tcf3, *etc.* Additional lncRNA-target-TFs network analysis was conducted for the top 20 differentially expressed lncRNAs. The results indicated Hnf4a, Ppara, Vdr, and Runx3 as the TFs most likely to regulate the production of these lncRNAs, and might play roles in inflammatory and metabolic processes in atherosclerosis. In a nutshell, the present study identified a panel of dysregulated lncRNAs and mRNAs that may be potential biomarkers or drug targets relevant to the high-fat diet related atherogenesis.

Atherosclerosis is a chronic multifactorial inflammatory disease with high prevalence worldwide and has become the leading cause of death^1^. The principal clinical manifestations of atherosclerosis are represented by coronary artery disease (CAD), acute myocardial infarction, cerebral stroke and peripheral vascular disease. Atherosclerosis represents a heterogeneous group of pathological phenomena that include endothelium damage, inflammation, metabolic disorder, cell proliferation, foam cell formation, and soft inflamed atherosclerotic plaque rupture^2^,^3^.

Long non-coding RNAs (lncRNAs; >200 bp) participate in many biological and pathological processes such as carcinogenesis and cardiovascular diseases by acting as signals, decoys, guides, and scaffolds^4^,^5^,^6^,^7^. Recently, the roles of lncRNAs in atherosclerosis have gained a lot of attention. It was reported that lncRNA-H19 and antisense noncoding RNA in the INK4 locus (ANRIL) are related to the risk of atherosclerosis and CAD^8^,^9^,^10^. Long intergenic noncoding RNA p21 (lincRNA-p21) is also reported to be associated with atherosclerosis through vascular smooth muscle cell proliferation and apoptosis^11^. However, the knowledge about the genome scale of lncRNAs and their potential biological functions in atherosclerosis are still far from clear.

In the present study, we investigated the lncRNA and mRNA expression differences in the aorta of the ApoE(−/−) mice fed with normal or high-fat diet using microarray analysis. The microarray results were further verified by qPCR. LncRNAs and mRNAs correlation network, gene ontology (GO; website: http://david.abcc.ncifcrf.gov/home.jsp) enrichment, and Kyoto Encyclopedia of Genes and Genomes (KEGG; websit: http://www.genome.jp/kegg) pathway analysis were performed to explore the potential functions of these differentially expressed lncRNAs. We further predicted the potential transcription factors (TFs) which may regulate the production of these lncRNAs. In order to identify the roles of individual lncRNA, we additionally analyzed the lncRNA-target-TFs network for top 20 most differentially expressed lncRNAs.

## Results

### The body weight and serum lipid levels of two group mice

**Figure 1:**
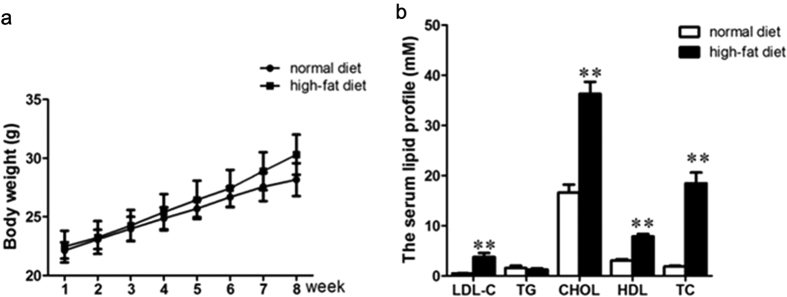
The body weight and serum lipid levels in normal diet and high-fat diet ApoE(−/−) mice. (**a**) the body weight changes in normal diet or high-fat diet fed ApoE(−/−) mice during 8-week treatment. (**b**) the serum lipid levels in ApoE(−/−) mice fed with normal diet or high-fat diet. Data are presented as means ± SD, n = 8. **P < 0.01 vs normal diet mice.

After 8 weeks of treatment, the body weight changes between the two diet groups were closely similar (Fig. 1a). However, LDL-C, CHOL and HDL-CH levels in high-fat diet group mice were dramatically higher than that in normal-diet group mice (*P* < 0.01, Fig. 1b).

### Differentially expressed lncRNAs in ApoE(−/−) mice

**Figure 2:**
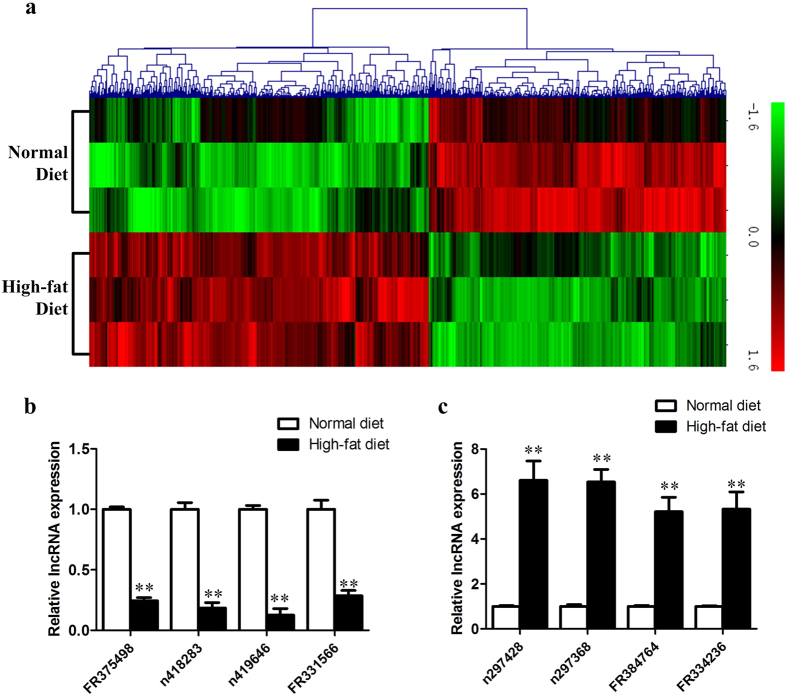
The differentially expressed lncRNAs in normal diet and high-fat diet fed apoE(−/−) mice. (**a**) the hierarchical clustering of the differentially expressed lncRNA probes in normal diet and high-fat diet fed apoE(−/−) mice. (**b**,**c**) the qPCR verification of 8 randomly selected differentially expressed lncRNAs.

Feature Extraction software (version10.7.1.1, Agilent Technologies) was used to analyze array images to get raw data. Genespring (version 13.1, Agilent Technologies) was employed to finish the basic analysis with the raw data. To begin with, the raw data was normalized with the quantile algorithm. The probes that have at least 1 out of 2 conditions and have flags in “P” were chosen for further data analysis. Differentially expressed mRNAs or lncRNAs were then identified through fold change (FC) as well as *p-*value calculated with t-test. The threshold set for up- or down-regulated RNAs was a FC ≥ 2.0 and a *p*-value ≤ 0.05. We scanned 51,302 lncRNA probes in normal diet and high-fat diet fed ApoE(−/−) mice, and identified 354 differentially expressed lncRNAs. Among them, 168 were up-regulated and 186 were down-regulated (Supplementary Table-S1). Next, hierarchical clustering was performed to display the distinguishable gene expression patterns among normal diet and high-fat diet apoE(−/−) mice (Fig. 2a).

To verify the results of lncRNA microarray, we detected the expression of 8 lncRNAs (4 up-regulated and 4 down-regulated) using qPCR. These lncRNAs were selected randomly from differentially expressed lncRNA transcripts. Figure 2b,c showed that the results were consistent with the lncRNA array analysis.

### Differentially expressed mRNAs in ApoE(−/−) mice

**Figure 3:**
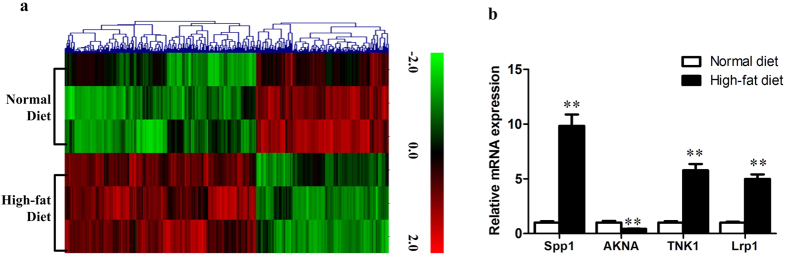
The differentially expressed mRNAs in normal diet and high-fat diet fed apoE(−/−) mice. (**a**) hierarchical clustering of the differentially expressed mRNA probes in normal diet and high-fat diet fed apoE(−/−) mice. (**b**) qPCR verification of 4 randomly selected differentially expressed mRNAs.

We also scanned the differentially expressed mRNAs between normal diet and high-fat diet fed ApoE(−/−) mice using the criteria of FC ≥ 2.0 and *p* ≤ 0.05. Among the 39430 mRNAs, 357 were found to be differentially expressed including 211 up-regulated and 146 down-regulated mRNAs (Supplementary Table-S2). Hierarchical clustering was performed and the results from mRNAs microarray were verified using qPCR by selecting 4 mRNAs randomly from the differentially expressed mRNA transcripts. Consistent results were found in the qPCR and mRNA microarray analysis (Fig. 3a,b).

### Co-expression network and potential functions identification

**Figure 4:**
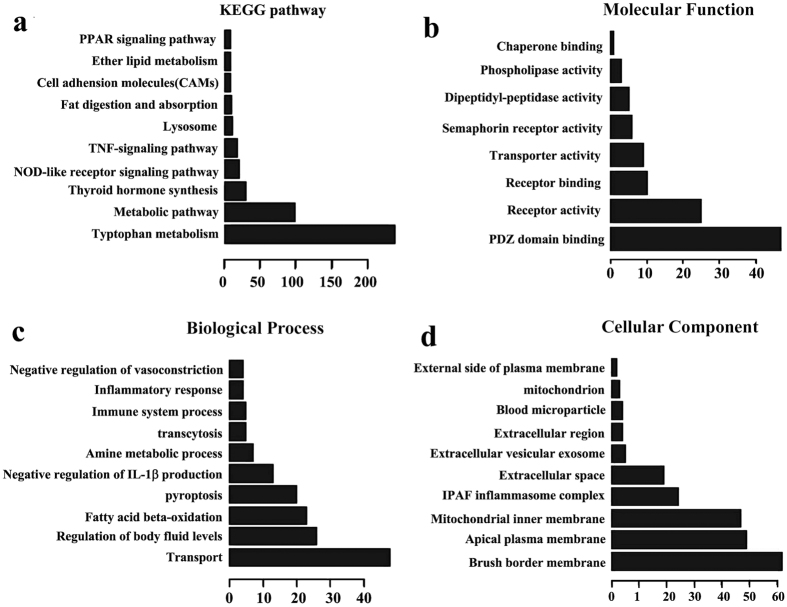
KEGG pathway and GO enrichment analysis of differentially expressed lncRNAs. The top 10 most enriched GO categories and pathways were calculated and plotted. (**a**) KEGG pathway; (**b**) Molecular Function; (**c**) Biological Process; (**d**) Cellular Component.

For each differentially expressed lncRNA, we investigated the correlation between each dysregulated mRNAs. We ranked the *p*-value of each lncRNA-mRNA correlation and selected the top 500 for functional enrichment analysis (Supplementary Table-S3). The enriched functional terms were used as the predicted functional terms of given lncRNAs. As shown in Fig. 4a and Supplementary Table-S5, KEGG pathway analysis indicated that, the lncRNAs were most likely related to metabolism (including tryptophan metabolism, metabolic pathway, and ether lipid metabolism) and inflammation (including NOD-like receptor signaling pathway, TNF signaling pathway, Cell adhesion molecules (CAMs), and PPAR signaling pathway). Since atherosclerosis is considered to be a metabolic disorder and inflammatory disease, these two procedures might be regulated by the aberrantly expressed lncRNAs. And for further GO enrichment, we found that the differentially expressed lncRNAs were mostly enriched in PDZ domain binding, receptor activity, and receptor binding in its Molecular Function (Fig. 4b); transport and body fluid levels regulation in its Biological Process (Fig. 4c); and membrane in its Cellular Component (Fig. 4d).

### LncRNA-TFs network analysis

**Figure 5:**
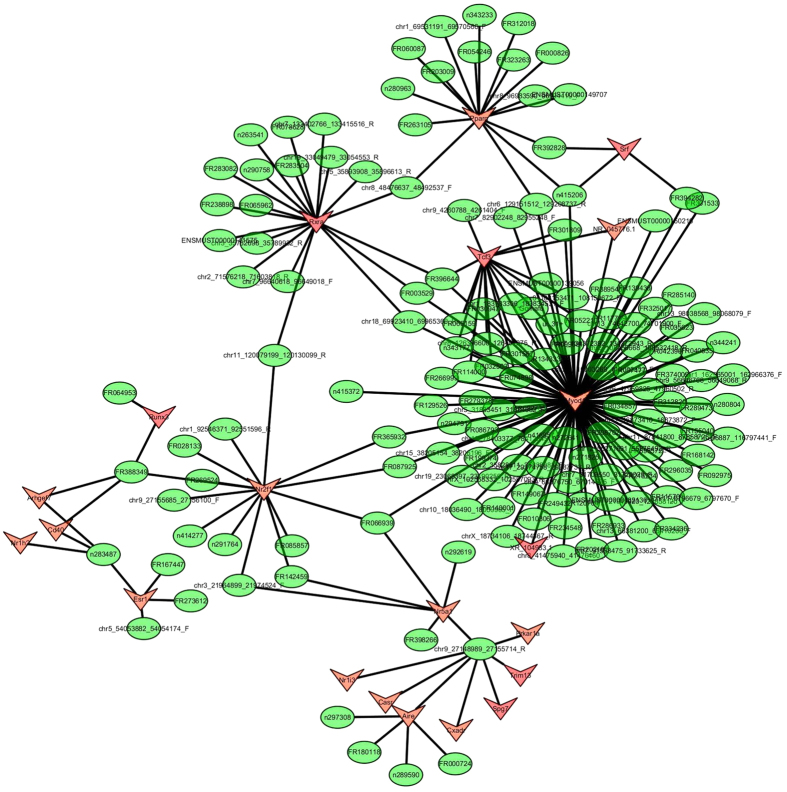
Network of the top 200 most related LncRNA-TFs pairs according to the *p* value. Red arrow: TFs; Green round: lncRNAs.

Since TFs were indicated to regulate the production of lncRNAs, we therefore predicted the TFs that could regulate the production of differentially expressed lncRNAs using hypergeometric distribution testing method. We selected 400 most differentially regulated lncRNAs (200 up-regulated and 200 down-regulated) for the prediction. Eventually, 395 out of the 400 lncRNAs have been predicted to be regulated by 283 TFs. We then selected the top 200 most related lncRNA-TF pairs according to the *p*-value, and found these lncRNAs were mostly regulated by Myod1, Rxra, Pparg, Tcf3, Nr2f1, Nr5a1, Aire, Esr1, Srf, Runx3, and Arhgef7 (Fig. 5, Supplementary Table-S6). Among these TFs, Myod1, Rxra, Pparg, Esr1, Srf, and Runx3 were previously demonstrated to be related with atherosclerosis^12^,^13^,^14^,^15^,^16^,^17^.

### LncRNA-target-TFs network analysis of 20 most differentially expressed lncRNAs

**Figure 6:**
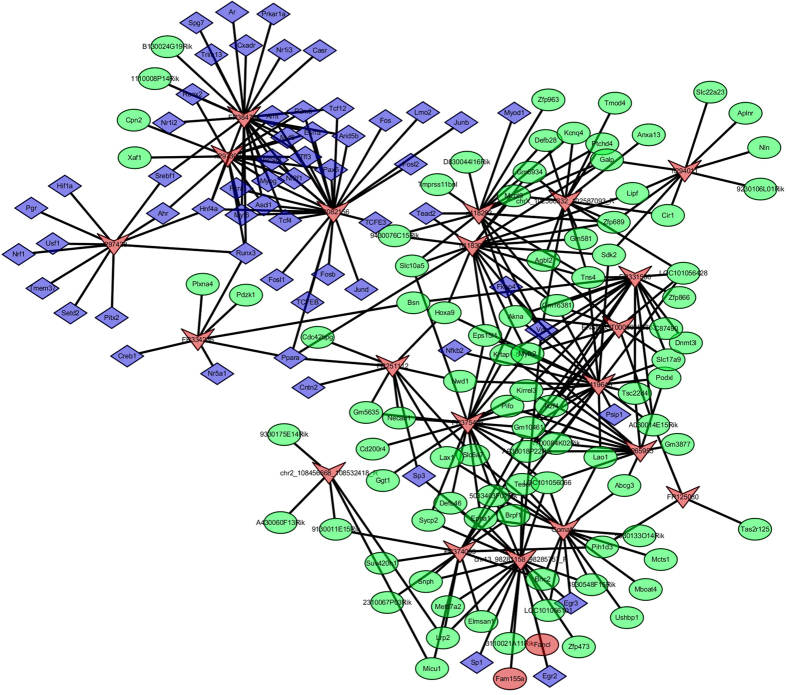
lncRNA-target-TFs network of 20 most differentially expressed lncRNAs. Red arrow: lncRNAs; Green round: target mRNAs; Blue diamond: TFs.

In order to further identify the functions of each individual lncRNA which may play critical roles in atherogenesis, we analyzed 20 most differentially expressed lncRNAs (10 up-regulated and 10 down-regulated) between normal diet and high-fat diet fed ApoE(−/−) mice. A total of 157 TFs were predicted to regulate the transcription of these lncRNAs. And also a total of 268 correlated mRNAs were predicted to be the targets of these lncRNAs. We selected 100 most related TFs and 200 most correlated mRNAs according to the *p*-value to conduct the lncRNA-target-TFs network analysis. The network included 170 nodes and 299 edges (Fig. 6). Among these TFs; Hnf4a, Ppara, Vdr, and Runx3 were predicted to regulate several lncRNAs. For example, Hnf4a was predicted to regulate the FR384764, n297308, FR082156 and n297428; Ppara was predicted to regulate the FR251112, FR082156, FR334236 and n418309; Vdr was predicted to regulate the n418309, n418283, chr13_98281158_98285757_F and chrX_102568332_102587093_R; Runx3 was predicted to regulate the n297308, FR384764, n297428 and FR334236. Moreover, all these four TFs were previously demonstrated to be related with atherosclerosis^17^,^18^,^19^,^20^. We also conducted the GO enrichment and KEGG pathway analysis for these lncRNA-targeted mRANs. The results indicated that they were most likely related to Molecular Functions like ion binding, transcription regulation, and KEGG pathways like tryptophan metabolism, neuroactive ligand-receptor interaction, ABC transporters, glutathione metabolism, intestinal immune network for IgA production, hedgehog signaling pathway, long-term depression, arachidonic acid metabolism, vascular smooth muscle contraction, cell adhesion molecules, *etc.* (Supplementary Figure-S1).

## Discussion

In the present study, we investigated the genome-wide expression profile of lncRNAs and mRNAs in normal diet and high-fat diet fed ApoE(−/−) mice using microarray. A total of 354 lncRNAs, including 168 up-regulated and 186 down-regulated were identified to be differentially expressed between the two groups of mice. Simultaneously, we also identified 357 differentially expressed protein coding mRNAs from the same chip, including 211 up-regulated and 146 down-regulated mRNAs. The expression differences of lncRNAs and mRNAs were consistent in qPCR detection with microarray detection.

In order to explore the functions of these differentially expressed lncRNAs, we conducted the co-expression analysis with the aberrantly expressed mRNAs. These mRNAs were then used to predict the potential function of the differentially expressed lncRNAs. The results indicated that these lncRNAs were mostly related to metabolisms and inflammation. Many studies have indicated that atherosclerosis is a metabolic disease. Our study found that tryptophan metabolism was the most affected pathway in KEGG analysis. Tryptophan is the least abundant amino acid of all essential amino acids, which are necessary for protein synthesis. The major catabolic route of Tryptophan in mammalians is the kynurenine pathway^21^. In kynurenine pathway, the indoleamine 2,3-dioxygenase (IDO) is firstly induced by inflammatory cytokines, such as interferon-γ (IFN-γ) and tumor necrosis factor-α (TNF-α), and then leads to the catabolic of kynurenine, causing an increase of Kynurenine/Tryptophan ratio, which is associated with the greater carotid plaque in atherosclerosis patients and in coronary heart disease patients^22^,^23^. Besides metabolism, these differentially expressed lncRNAs also participated in inflammatory signal pathways, such as NOD-like receptor signaling pathway, TNF signaling pathway, CAMs, and PPAR signaling pathway. These inflammatory pathways were widely researched and demonstrated to be associated with atherogenesis^24^,^25^,^26^. Therefore, it’s reasonable to propose that the aberrantly expressed lncRNAs participated in the diet-induced atherogenesis, through affecting its correlated metabolic or inflammatory mRNAs.

In the Molecular Function of GO enrichment assay, we found “PDZ domain binding” was the most enriched term for these lncRNA-correlated mRNAs. PDZ binding proteins which binds to the carboxy-termini of a number of membrane transporter proteins, including ion channels and cell surface receptors, were found to play key roles in lipoprotein metabolism and atherosclerosis^27^,^28^. Thus, “PDZ domain binding”, in addition to “receptor activity” and “receptor binding” in its Molecular Function, and “membrane” in its Cellular Component, might explain the way these lncRNAs play their roles in diet-induced atherogenesis.

Furthermore, we predicted the TFs which might regulate the production of lncRNAs via hypergeometric distribution analysis. We found that Myod1, Rxra, Pparg, Tcf3, Nr2f1, Nr5a1, Aire, Esr1, Srf, Runx3, and Arhgef7 were among the mostly correlated TFs. Previous studies have demonstrated that Rxra, Pparg, Nrf1, and Srf were associated with atherosclerosis through lipid metabolism or transportation^14^,^29^,^30^; vascular smooth muscle cell proliferation^31^; inflammation^32^,^33^; Esr1 and Runx3 were related to the risk of atherosclerosis^15^,^17^. Interestingly, we found that Myod1 was the most correlated TF with lncRNAs in the present study. Previous studies, however, are mostly focused on its effects on muscle differentiation and regeneration^34^. How it acts in atherogenesis still need further investigation.

To further study the roles of specific lncRNAs in atherogenesis, we analyzed the relationship between 20 most differentially expressed lncRNAs with TFs and their target mRNAs. LncRNA-target-TFs network analysis found several most likely TFs which were for the transcription of these lncRNAs. These include Hnf4a, Ppara, Vdr, and Runx3. Interestingly, we found FR384764, n297308, and n297428 were predicted to be regulated by both Hnf4a and Runx3. Further pathway analysis indicated that these 3 lncRNAs were mostly related to inflammatory process, lipid or amino acid metabolism, and oxidative phosphorylation. Since both Hnf4a and Runx3 demonstrated to have functions on lipid metabolism or inflammation^17^,^19^, we presumed that these two TFs work together with FR384764, n297308 and n297428 to regulate the expression of certain mRNAs, and eventually affect the procedures of metabolism or inflammatory process to control the atherogenesis.

In our present study, we compared the lncRNAs and mRNAs expression profile between normal diet and high-fat diet fed ApoE(−/−). Previous studies have demonstrated that important roles of diet in the formation of atherosclerosis in ApoE(−/−) mice. High-fat and high-cholesterol diet induced atherosclerosis faster and more severe than normal diet in ApoE(−/−) mice^35^,^36^. The diet also affected the intimal hyperplasia significantly^37^. Our present study found a significant difference in the lipid levels between these two groups. Some differentially expressed lncRNAs and mRNAs were found between them as well, which we presume to represent the effects of diet on RNA profile during the progress of atherosclerosis. However, ApoE(−/−) mice spontaneously exhibit atherosclerosis. Even those ApoE(−/−) mice fed with normal diet showed spontaneous elevations in plasma cholesterol compared with wild-type C57BL6J mice because of impaired clearance of cholesterol-rich remnant particles. Therefore, the RNA profile between wild-type C57BL6J mice and ApoE(−/−) mice still needs further analysis.

H19 is an imprinted gene, first discovered as a tumor suppressor. In 1996, Han *et al.* found that H19 was expressed in the adult human atherosclerotic lesion^10^. Further studies have found that hyperhomocysteinemia and Hcy treatment causes hypomethylation, which subsequently increases the expression of H19 in adult mice or VSMCs^38^,^39^. Another study found a common polymorphism of H19 which was associated with increasing the risk and severity of CAD in a Chinese population^8^. All these research indicated that H19 is related to atherosclerosis. In our present study, we also found a high expression of H19 in both normal diet group and high-fat diet group. Since ApoE(−/−) mice spontaneously exhibit atherosclerosis despite the diet, the results indicated that the high levels of H19 expression found in these mice might contributed to the spontaneous development of atherosclerosis. However, no significant difference between the normal diet and high-fat diet group of ApoE(−/−) mice was found in our study, which might mean that the diet is not the cause of H19 expression changes.

Taking all these together, we have identified a panel of dysregulated lncRNAs and mRNAs that may be potential biomarkers or drug targets relevant to the high-fat diet related atherogenesis.

## Materials and Methods

### Animals and diets

The homozygous male ApoE(−/−) mice, 22–24 g, were obtained from Shanghai Slac Laboratory Animal Co., Ltd (Shanghai, People’s Republic of China). The care and use of these animals were following the institutional and national guidelines. All experimental procedures involving animals were approved by the Ethics Committee of the Central South University, China. The mice were housed in a temperature-controlled room (22 ± 0.8 °C) and a relative humidity of 55 ± 10%, with 12 h light-dark cycles and free access to water and chow. The ApoE(−/−)(−/−) mice were randomly divided into two groups with 8 animals in either. The normal diet group was fed with normal chow, while the treated group was fed with high-fat and high-cholesterol diet containing 20% fat and 2.5% cholesterol. The treatment duration is 8 weeks. The body weight of each group of mice was monitored weekly. At the end of the treatment, the mice were anesthetized by intraperitoneal injection of 10% chloral hydrate (0.4 mL/100 g body weight), and the blood was collected from the retro-orbital plexus. The serum lipid levels of each mouse were detected using automatic biochemical analyzer (Selectra E). After perfusion with ice-cold normal saline, the aorta of each mouse was taken and stored in liquid nitrogen until further analysis. We selected 3 aorta samples from each group randomly for the detection of lncRNAs and mRNAs array. Other samples were prepared for further verification of microarray results using qPCR.

### Array data production

The experiments were performed in the lab of OeBiotech Corporation (Shanghai, China). Agilent mouse lncRNA Microarray (4*180K, Design ID: 049801), which contains 51302 lncRNAs and 39430 mRNAs, was used in this experiment. To sum up, the total RNA was extracted using Trizol reagent and then quantified using the NanoDrop ND-2000 (Thermo Scientific). The RNA integrity was assessed using Agilent Bioanalyzer 2100 (Agilent Technologies). The sample labeling, microarray hybridization, and washing were performed based on the manufacturer’s standard protocols. Then, the total RNA was transcribed to double strand cDNA, synthesized into cRNA and labeled with Cyanine-3-CTP. The labeled cRNA was hybridized onto the microarray. And finally, the arrays were scanned by the Agilent Scanner G2505C(Agilent Technologies) after washing.

### Differential expression analysis

Data analysis was performed using the GeneSpring GX v13.1 software package (Agilent Technologies, Santa Clara, USA). The Quantile method was used to normalize the raw data. After normalization, the data with a 100% “P” flags in at least one group (normal diet group or high-fat diet group) were selected for further analysis. Differentially expressed lncRNAs and mRNAs were assessed using the t-test. The FC ≥ 2.0 between the compared two groups, as well as the *p*-value < 0.05, was defined as differentially expressed lncRNAs or mRNAs. Hierarchical Clustering was performed using the Agilent GeneSpring GX software (version 11.5.1).

### Quantitative RT-PCR

QRT-PCR was used to validate some of the differentially expressed lncRNAs and mRNAs. The primers were listed in Table 1. The qPCR was performed using the SYBR Green Premix DimerEraser kit (TaKaRa Bio Inc., Dalian, China) on the Roche LightCycler 480 Instrument II. The relative gene expression was analyzed using the 2^(−ΔΔCt)^ Method as previously described^40^. The significance of differences was analyzed using one-way analysis of variance or Student’s t-test (unpaired). Multiple comparisons between groups were performed using Tukey’s method. SPSS software, version 19.0 (SPSS, Inc., Chicago, IL, USA) was used to perform the statistical analysis. *p* < 0.05 was considered to indicate a statistically significant difference.

### Co-expression network analysis

The co-expression of lncRNA and mRNA was calculated by the Pearson correlation coefficients (PCC). The correlated *p* < 0.05 was considered as statistically significant. We also detected the correlations between lncRNAs and TFs via hypergeometric distribution analysis in MATLAB environment. Firstly, the mRNAs which are co-expressed with the aberrant lncRNAs were identified. The overlap of the co-expressed mRNAs set with TFs target genes set was calculated. Then hypergeometirc distribution analysis was used in calculating the significance of this overlap. If the co-expressed mRNAs of the given lncRNAs significantly overlapped with the target genes of the given TFs, the TFs would then be considered as interacting with these lncRNAs. The most recent data released by the Encyclopedia of DNA Elements (ENCODE) on TFs and their targets were used in the present analysis^41^,^42^. The graph of the lncRNAs-TFs network was drawn with the help of Cytoscape 3.11 (Agilent and IBS).

### Functional classification and pathway analysis

The function of lncRNA co-expressed mRNA was analyzed using the Gene Ontology enrichment analysis. The GO analysis was divided into Molecular Function, Biological Process and Cellular Component. The pathway of lncRNA co-expressed mRNA was analyzed using the KEGG pathway analysis. These analyses allowed us to predict the functional classification and biological pathway in which the co-expressed mRNAs of the differentially expressed lncRNAs are enriched. A *p* < 0.05 was considered statistically significant for the correlation.

### Data Availability

The authors confirm that all data underlying the findings are fully available without restriction. All relevant data are within the paper and its Supporting Information files or in a public repository (GEO series accession number GSE83112 (http://www.ncbi.nlm.nih.gov/geo/query/acc.cgi?acc = GSE83112).

## Additional Information

**How to cite this article**: Bao, M.-h. *et al.* Impact of high fat diet on long non-coding RNAs and messenger RNAs expression in the aortas of ApoE(−/−) mice. *Sci. Rep.* **6**, 34161; doi: 10.1038/srep34161 (2016).

## Supplementary Material

Supplementary Information

## Figures and Tables

**Table 1:** The primers used for qPCR detection of selected lncRNAs and mRNAs.

Gene name	Forward	Reverse
n419646	CGGCTTTGTGGAGGATTAGA	TGGTAGGTGCTGGGTGTTG
FR0334236	ATGACCCCCTCTGACTTCCT	TCACAAACTGACTGGCTGGA
FR384764	AGGGATGTGGCTGTTGACTT	CCAGGTTGCTGTAGGTCTCC
N297368	ACACTGCCACATCCTTGTTG	CCATAATCTACACCCCACACC
N297428	AGGGCTTCTCTTCTCTTCACAG	TCTCCCAACGCCTCATAAAG
N418283	CCTCCCAGGTTGAAGTGATT	AAGCCCGTCTCTGCTAAAAATA
FR375498	CCCGTGAATGTAACTCCTGAC	TGTGTTCCTCTCCCCTTCTG
FR331566	CACCCATTTGCTCGTAGACC	GGAACATTGGCATCAGTGG
AKNA	CAAGCAGAGGAGCAAGCAG	TGGGAACCGAGGAGATGTAG
TNK1	GAAAACCCCCACACAATCAC	GCTCCACCTCCATAATCTTCC
Spp1	GGATTCTGTGGACTCGGATG	CGACTGTAGGGACGATTGGA
Lrp1	GAGGAGCGAGGAGTAAAGCA	GGGGCATAGGTGAAATGGTA
β-actin	GACTGACTACCTCATGAAGAT	CATGATGGAGTTGAAGGTAGTT
