# Supplementary material for: Impact of high fat diet on long non-coding RNAs and messenger RNAs expression in the aortas of ApoE(−/−) mice
Source: Sci Rep. 2016 Oct 4;6:34161. doi: 10.1038/srep34161 (PMC5048419; doi:10.1038/srep34161)
Supplement: Supplementary Information [file srep34161-s1.pdf]

Impact of high fat diet on long non-coding RNAs and messenger RNAs expression in the aortas of ApoE(-/-) mice

Mei-hua Bao[1*], Huai-qing Luo[1], Li-hua Chen[2], Liang Tang[1], Kui-fen Ma [3], Ju Xiang[1], Li-ping Dong[1], Jie Zeng[1], Guang-yi Li[1], Jian-ming Li[1,4,5*]

[1] Department of Anatomy, Histology and Embryology, Institute of of Neuroscience, Changsha Medical University, Changsha, 410219, China. E-mails: luohuaiqing@163.com (Huai-qing Luo); tlcool318@163.com (Liang Tang); Xiang.ju@foxmail.com (Ju Xiang); ddongliping@163.com (Li-ping Dong); zengjie84117@163.com (Jie Zeng); 470064429@163.com (Guang-yi Li)

[2] The Third Xiangya Hospital of Central South University, Changsha 410013, Hunan, PR China. Email: chenlihuacc@163.com (Li-hua Chen).

[3] The First Affiliated Hospital, Zhejiang University, Hangzhou, Zhejiang, PR Chian. Email: jancemkf@163.com (Kui-fen Ma)

[4] Department of Neurology, Xiangya Hospital, Central South University, Changsha, Hunan 410008, China

[5] Department of Anatomy, Xiangya School of Medicine, Central South University , Changsha, Hunan 410013,China)

[*] Correspondence to:

Mei-hua Bao, PhD, OR Jian-ming Li, PhD

Department of Anatomy, Histology and Embryology, Institute of Neuroscience, Changsha Medical University, Changsha, 410219, China. Tel: +86 731 88602839; Fax: +86 731 88602669

Email: mhbao78@163.com (Mei-hua Bao); ljming0901@sina.com (Jian-ming Li)

Figure-S1 KEGG pathway and GO enrichment (Molecular Function) analysis of FR384764, n297308, n297428 and n418309 correlated mRNAs.

Table-S1: Significantly and differentially expressed lncRNAs in high-fat and normal diet ApoE(-/-) mice

Table-S2: Significantly and differentially expressed mRNAs in high-fat and normal diet ApoE(-/-) mice

Table-S3 Top 500 most correlated LncRNAs and mRNAs

Table-S4 GO functional enrichment analysis of top 500 lncRNA-correlated mRNAs

Table-S5 KEGG pathway analysis of top 500 lncRNA-correlated mRNAs

Table-S6 200 most significant related TF-lncRNA pairs

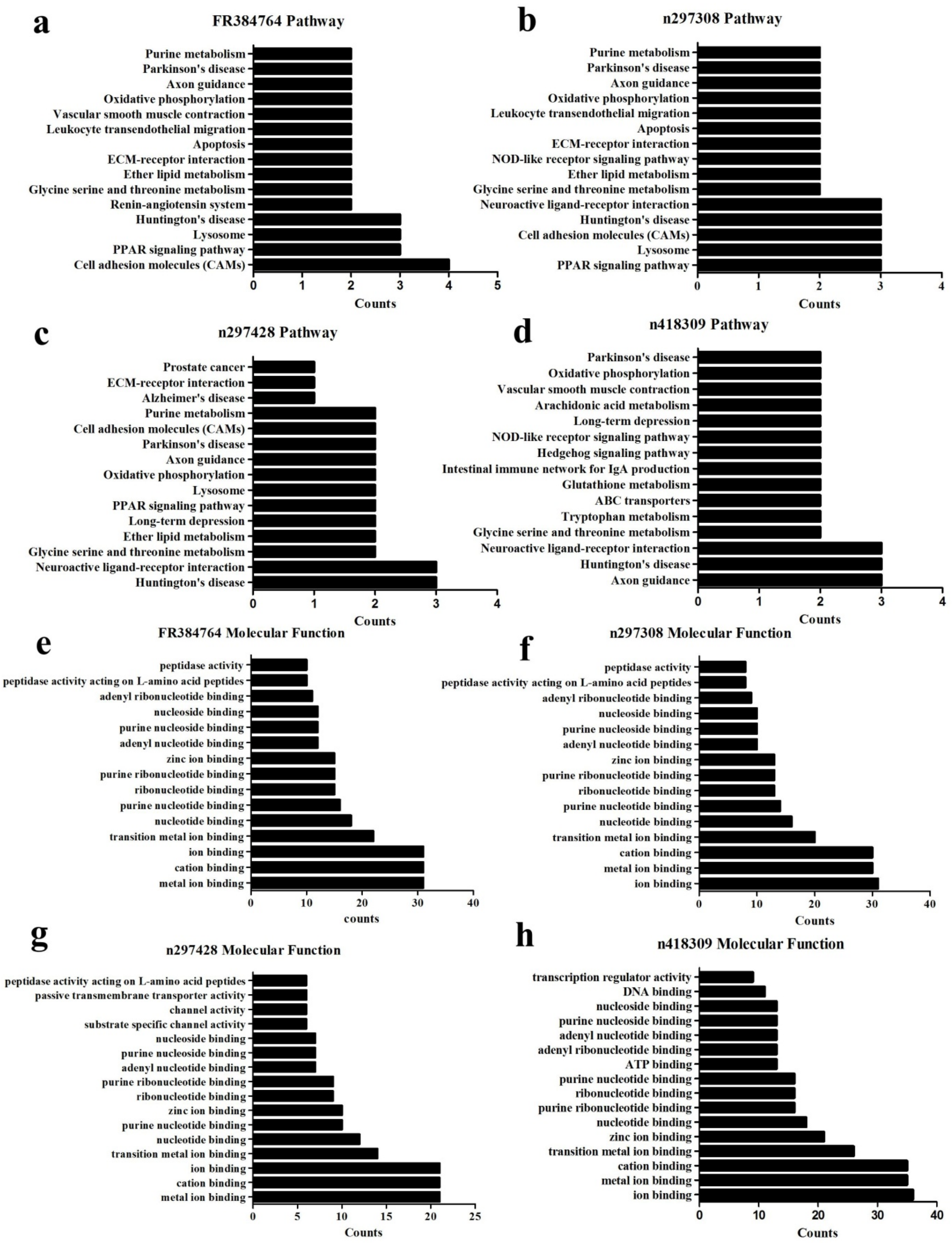

Figure S1 KEGG pathway and GO enrichment (Molecular Function) analysis of FR384764, n297308, n297428 and n418309 correlated mRNAs. The top 15 most enriched GO (Molecular Function) categories and KEGG pathways were calculated and plotted. a-d, KEGG pathway for FR384764, n297308, n297428 and n418309; e-h, GO (Molecular Function) categories for FR384764, n297308, n297428 and n418309

Table-S1: Significantly and differentially expressed lncRNAs in high-fat and normal diet ApoE(-/-) mice

ProbeName	p	FC (abs)	Regulation	BioType	Primary.Accession
mmu_19889_PI428960544	0.023563156	6.6065483	up	ncRNA	FR326589
mmu_22142_PI428960544	0.00770111	6.5403714	up	ncRNA	FR194887
mmu_5643_PI428960544	0.028952846	5.4900584	up	ncRNA	FR078628
mmu_12355_PI428960544	0.038484745	5.279569	up	ncRNA	FR134333
mmu_23341_PI428960544	0.009124966	4.8421855	up	ncRNA	FR204953
mmu_23966_PI428960544	0.002932061	4.5948863	up	ncRNA	FR082156
mmu_23928_PI428960544	0.03821654	4.3853273	up	ncRNA	FR289473
mmu_20538_PI428960544	0.048980407	4.3433394	up	ncRNA	FR279620
mmu_963_PI428960544	0.015355721	4.145839	up	lincRNA	ENSMUST00000150216
mmu_650_PI428960544	0.017392438	3.9888875	up	lincRNA	ENSMUST00000149707
mmu_21368_PI428960544	0.013935655	3.8130763	up	ncRNA	FR279378
mmu_12484_PI428960544	0.03792329	3.6627505	up	ncRNA	FR301809
mmu_8491_PI428960544	0.048261233	3.6376286	up	ncRNA	FR208702
mmu_7133_PI428960544	0.036832657	3.557992	up	ncRNA	FR000826
mmu_16075_PI428960544	0.002524217	3.5169146	up	ncRNA	FR278883
mmu_30153_PI428960544	0.04753332	3.5146444	up	mRNAlike lncRNA	n289317
mmu_24149_PI428960544	0.019089084	3.5034988	up	ncRNA	FR347914
mmu_23919_PI428960544	0.008941423	3.3492064	up	ncRNA	FR247760
mmu_28104_PI428960544	0.00575809	3.3455012	up	mRNAlike lncRNA	n263575
mmu_2818_PI428960544	0.03773945	3.312418	up	ncRNA	FR268824
mmu_11081_PI428960544	0.020598492	3.311713	up	ncRNA	FR392828
A_30_P01018797	0.01600619	3.2422783	up	unknown	chr15:38205154-38206196_F
mmu_3548_PI428960544	0.027604822	3.2340698	up	ncRNA	FR120704
A_30_P01021354	0.017083308	3.2002678	up	unknown	chr18:5813291-5813708_R
mmu_2136_PI428960544	0.040144473	3.1492121	up	ncRNA	FR092975
mmu_12879_PI428960544	0.040874362	3.1173925	up	ncRNA	FR168142
mmu_13420_PI428960544	0.01950497	3.1114726	up	ncRNA	FR398266
mmu_6376_PI428960544	0.005027039	3.103433	up	ncRNA	FR323263
mmu_11157_PI428960544	0.046267338	3.08132	up	ncRNA	FR040835
A_30_P01018166	0.036979765	3.0806632	up	unknown	chr7:134372392-134372943_R
mmu_1274_PI428960544	0.001154897	3.0332644	up	lincRNA	ENSMUST00000125309
mmu_14003_PI428960544	0.029083358	3.0099747	up	ncRNA	FR254927
mmu_11823_PI428960544	0.024438512	2.965918	up	ncRNA	FR017535
A_30_P01032080	0.010737287	2.954146	up	unknown	chr18:70400082-70404236_R

mmu_3078_PI428960544	0.023029735	2.9380777	up	ncRNA	FR325017
mmu_16214_PI428960544	0.02486451	2.9271333	up	ncRNA	FR155040
A_30_P01020159	0.026785105	2.9209218	up	unknown	chr11:6796679-6797670_F
mmu_5104_PI428960544	0.04391799	2.906936	up	ncRNA	FR149067
mmu_25931_PI428960544	0.017815096	2.9042013	up	ncRNA	FR030947
mmu_17866_PI428960544	0.032166865	2.895322	up	ncRNA	FR117744
mmu_2940_PI428960544	0.030127095	2.8804865	up	ncRNA	FR389545
A_30_P01032005	0.047144275	2.8385632	up	unknown	chr13:98038568-98068079_F
mmu_34905_PI428960544	0.039697703	2.8361952	up	ncRNA	XR_141936.2
mmu_32769_PI428960544	0.028759228	2.8340204	up	lncRNA	n415282
mmu_34077_PI428960544	0.009751953	2.8175285	up	ncRNA	XR_140871.1
mmu_23856_PI428960544	0.04172041	2.8053327	up	ncRNA	FR028133
mmu_13666_PI428960544	0.024528423	2.7964616	up	ncRNA	FR142459
A_30_P01029641	0.026217261	2.7920523	up	unknown	chr5:31883451-31884082_F
mmu_25297_PI428960544	0.043720126	2.7866087	up	ncRNA	FR124060
mmu_5087_PI428960544	0.015784875	2.7857049	up	ncRNA	FR334236
mmu_25672_PI428960544	0.025070107	2.7848315	up	ncRNA	FR112324
mmu_30578_PI428960544	0.010306944	2.769985	up	mRNAlike lncRNA	n292619
mmu_30276_PI428960544	0.0075121	2.7561219	up	mRNAlike lncRNA	n290544
mmu_3781_PI428960544	0.048080303	2.7474217	up	ncRNA	FR186619
mmu_4508_PI428960544	0.016159829	2.7340727	up	ncRNA	FR150621
mmu_29058_PI428960544	0.03210392	2.7291615	up	mRNAlike lncRNA	n273954
A_30_P01023930	0.013882695	2.7205498	up	unknown	chr14:33923944-34003394_F
mmu_12982_PI428960544	0.020821778	2.7023633	up	ncRNA	FR100205
mmu_26983_PI428960544	0.009816133	2.6920204	up	ncRNA	FR395103
mmu_26731_PI428960544	0.037888624	2.691197	up	ncRNA	FR233225
mmu_179_PI428960544	0.03264019	2.6338413	up	lincRNA	ENSMUST00000134627
mmu_27496_PI428960544	0.038659267	2.6007533	up	ncRNA	FR065159
mmu_26_PI428960544	0.02758966	2.6001775	up	ncRNA	Gomafu
mmu_33968_PI428960544	0.03291615	2.5979187	up	ncRNA	NR_045776.1
mmu_9384_PI428960544	8.55E-04	2.594224	up	ncRNA	FR007551
A_30_P01022813	0.005008529	2.5852578	up	unknown	chr10:39666719-39679959_R
A_30_P01031372	0.021731475	2.5756884	up	unknown	chr3:87757295-87774970_R
mmu_28896_PI428960544	0.044392936	2.5640917	up	mRNAlike lncRNA	n271825
mmu_3871_PI428994341	0.022747232	2.556269	up	ncRNA	uc.411+

mmu_29547_PI428960544	0.03551171	2.5496967	up	mRNAlike lncRNA	n280963
A_30_P01027923	0.02887718	2.5425677	up	unknown	chr2:116796887-116797441_F
mmu_33795_PI428960544	0.008219932	2.5424557	up	ncRNA	XR_105914.2
A_30_P01018796	0.001646837	2.5223703	up	unknown	chr6:115468025-115489250_R
mmu_20225_PI428960544	0.033889852	2.5207129	up	ncRNA	FR090778
A_30_P01027887	0.010532687	2.5182033	up	unknown	chr6:47959825-47960502_R
mmu_12301_PI428960544	0.019807743	2.506138	up	ncRNA	FR391590
mmu_7034_PI428960544	0.037889376	2.5056431	up	ncRNA	FR034857
mmu_10617_PI428960544	0.011292373	2.5050325	up	ncRNA	FR333763
mmu_5073_PI428960544	0.037074327	2.5026336	up	ncRNA	FR375933
A_30_P01025310	0.036782425	2.499172	up	unknown	chr2:108456668-108532418_R
mmu_15412_PI428960544	0.005050104	2.495746	up	ncRNA	FR351980
mmu_8772_PI428960544	0.022181565	2.4942906	up	ncRNA	FR125080
mmu_794_PI428960544	0.047215775	2.494239	up	lincRNA	ENSMUST00000139056
mmu_19296_PI428960544	0.04319484	2.4895132	up	ncRNA	FR202182
mmu_35640_PI428960544	0.04016116	2.464859	up	ncRNA	NR_033641.2
A_30_P01029190	0.038305547	2.4646716	up	unknown	chr8:41475940-41476460_F
mmu_21962_PI428960544	0.007326488	2.4407306	up	ncRNA	FR330268
mmu_24266_PI428960544	0.042930093	2.398131	up	ncRNA	FR090859
mmu_31453_PI428960544	0.002365315	2.3979487	up	mRNAlike lncRNA	n297308
mmu_8275_PI428960544	0.040743016	2.395396	up	ncRNA	FR286933
mmu_4608_PI428960544	0.043318145	2.3918982	up	ncRNA	FR117799
mmu_24659_PI428960544	0.046140507	2.3859751	up	ncRNA	FR114000
mmu_34053_PI428960544	0.049227282	2.373831	up	ncRNA	XR_104983.1
A_30_P01032161	0.04683037	2.372761	up	unknown	chr1:75075500-75102200_R
mmu_15584_PI428960544	0.043778464	2.3314264	up	ncRNA	FR032503
mmu_8650_PI428960544	0.026800226	2.315048	up	ncRNA	FR106089
mmu_20514_PI428960544	0.041017815	2.3125463	up	ncRNA	FR074898
A_30_P01020003	0.04380446	2.3050747	up	unknown	chr14:77511189-77522852_F
mmu_6507_PI428990136	0.03990378	2.2886844	up	mRNAlike lncRNA	n294791
A_30_P01021502	0.007168382	2.2868297	up	unknown	chr2:157236070-157249445_R
A_30_P01018490	0.010897614	2.2779417	up	unknown	chr8:123901815-123902167_R

mmu_29019_PI428960544	0.03553014	2.2725246	up	mRNAlike lncRNA	n273521
A_30_P01023218	0.016908493	2.2713208	up	unknown	chr18:38836368-38935650_F
A_30_P01023590	0.009824371	2.2698638	up	unknown	chr8:96983590-96994419_F
mmu_19347_PI428960544	0.037541483	2.2686877	up	ncRNA	FR374009
mmu_27624_PI428960544	0.045829844	2.2664516	up	ncRNA	FR234548
mmu_10219_PI428990136	0.031872008	2.266443	up	ncRNA	FR382845
mmu_32790_PI428960544	0.030185405	2.2590914	up	lncRNA	n415323
mmu_7917_PI428990136	0.036564685	2.252442	up	lncRNA	n416957
mmu_12259_PI428960544	0.03304125	2.2468956	up	ncRNA	FR171433
mmu_27231_PI428960544	0.036587734	2.2451406	up	ncRNA	FR115704
mmu_7024_PI428960544	0.002463199	2.2381797	up	ncRNA	FR302411
mmu_22632_PI428960544	0.007559009	2.233079	up	ncRNA	FR060087
A_30_P01031117	0.03083943	2.2303858	up		chr14:73729977-73751877_R
mmu_32835_PI428960544	0.036067802	2.2250137	up	lncRNA	n415399
mmu_17330_PI428960544	0.046079483	2.224169	up	ncRNA	FR269524
mmu_34221_PI428960544	0.031710315	2.2170591	up	ncRNA	NR_045899.1
mmu_32820_PI428960544	0.041244015	2.2163415	up	lncRNA	n415372
mmu_4683_PI428960544	0.032453883	2.2124991	up	ncRNA	FR003993
A_30_P01027909	0.026618496	2.2103121	up	unknown	chr8:48476637-48492537_R
mmu_27371_PI428960544	0.029853629	2.2081804	up	ncRNA	FR044110
A_30_P01033308	0.010824242	2.2074087	up	unknown	chr3:21964899-21974524_F
mmu_22029_PI428960544	0.006356597	2.2064266	up	ncRNA	FR263105
mmu_5571_PI428960544	0.033310674	2.2026677	up	ncRNA	FR283503
mmu_21236_PI428960544	0.04997005	2.1980102	up	ncRNA	FR301567
mmu_2490_PI428960544	0.034862373	2.1943767	up	ncRNA	FR046015
mmu_1774_PI428960544	0.042885456	2.190391	up	misc_RNA	ENSMUST00000158863
mmu_10236_PI428960544	0.0325216	2.188417	up	ncRNA	FR042390
A_30_P01020618	0.015307171	2.173115	up	unknown	chr3:21964899-21974885_F
A_30_P01029980	0.013885483	2.168227	up	unknown	chr16:44720170-44723911_R
mmu_20936_PI428960544	0.026254963	2.1682143	up	ncRNA	FR312018
mmu_22806_PI428960544	0.047292385	2.1537068	up	ncRNA	FR007873
A_30_P01033059	0.04722711	2.1501608	up	unknown	chr13:74642700-74701900_F
mmu_8777_PI428990136	0.01678756	2.148299	up	ncRNA	NR_076393.1
mmu_11584_PI428960544	0.017224615	2.1373372	up	ncRNA	FR064953
mmu_21306_PI428960544	0.029599363	2.1371691	up	ncRNA	FR387804
mmu_4919_PI428960544	0.030656677	2.1349902	up	ncRNA	FR019091

mmu_31141_PI428960544	0.03829869	2.1335437	up	mRNAlike lncRNA	n295927
A_30_P01026093	0.030422924	2.1327136	up	unknown	chr4:123572090-123581365_F
mmu_19378_PI428960544	0.032811638	2.132594	up	ncRNA	FR029539
mmu_628_PI428990136	0.029572587	2.128087	up	ncRNA	FR043548
mmu_3101_PI428960544	0.04058376	2.123979	up	ncRNA	FR001172
A_30_P01028704	0.037502278	2.1123943	up	unknown	chr1:69531191-69570566_F
mmu_18963_PI428960544	0.01957074	2.1121643	up	ncRNA	FR136228
mmu_11492_PI428960544	0.002826148	2.1115856	up	ncRNA	FR384764
mmu_35520_PI428960544	0.011742999	2.1050763	up	ncRNA	NR_073368.1
mmu_31483_PI428960544	0.020259283	2.1040065	up	mRNAlike lncRNA	n297428
mmu_10205_PI428960544	0.049763545	2.1008732	up	ncRNA	FR319563
mmu_28019_PI428960544	0.026878856	2.09898	up	ncRNA	FR199374
mmu_22177_PI428960544	0.03932856	2.0946405	up	ncRNA	FR394282
mmu_4095_PI428960544	0.049991973	2.0877526	up	ncRNA	FR140001
A_30_P01028297	0.019010816	2.082816	up	unknown	chr6:129151512-129208737_R
mmu_30244_PI428960544	0.018782414	2.0738676	up	mRNAlike lncRNA	n290305
mmu_4301_PI428960544	0.02303339	2.0653408	up	ncRNA	FR396644
mmu_28406_PI428960544	0.047664855	2.0561695	up	mRNAlike lncRNA	n265736
A_30_P01018273	0.04074268	2.0560977	up	unknown	chr12:55271691-55276492_R
mmu_3844_PI428960544	0.00968833	2.0468798	up	ncRNA	FR167447
mmu_4083_PI428990136	0.0399365	2.0432239	up	ncRNA	FR086793
mmu_32730_PI428960544	0.047250725	2.036267	up	lncRNA	n415206
A_30_P01020096	0.04601822	2.0341218	up	unknown	chr14:14688472-14747272_F
A_30_P01019611	0.012922458	2.033711	up	unknown	chr7:82902248-82955248_F
mmu_31184_PI428960544	0.019483639	2.0326414	up	mRNAlike lncRNA	n296070
mmu_31652_PI428960544	0.006439674	2.0230834	up	lincRNA	n343233
A_30_P01026958	0.013192127	2.014876	up	unknown	chr10:18036490-18059965_F
A_30_P01025459	0.01128211	2.0123346	up	unknown	chr13:34693404-34717987_F
mmu_8762_PI428960544	0.032639958	2.0102925	up	ncRNA	FR066939
mmu_34273_PI428960544	0.020541158	2.0008893	up	ncRNA	NR_033764.1
A_30_P01032003	0.03989104	2.0007617	up	unknown	chr13:107822042-107823753_F

A_30_P01032459	0.030034743	6.9042463	down	unknown	chrX:102568332-102587093_R
mmu_33288_PI428960544	0.01764818	4.2644243	down	lncRNA	n418283
mmu_21105_PI428960544	0.031598497	4.136219	down	ncRNA	FR375498
mmu_28436_PI428960544	0.038946223	4.1236954	down	mRNAlike lncRNA	n265955
mmu_33300_PI428960544	0.011352751	4.014023	down	lncRNA	n418309
mmu_33423_PI428960544	0.014372224	3.942122	down	lncRNA	n419646
A_30_P01026041	0.026998533	3.8855588	down	unknown	chr13:98281158-98285757_F
mmu_12453_PI428960544	0.03952362	3.6667264	down	ncRNA	FR331566
mmu_30778_PI428960544	0.04003301	3.5566716	down	mRNAlike lncRNA	n294011
mmu_27139_PI428960544	0.004500653	3.5109322	down	ncRNA	FR251112
A_30_P01022596	0.01831967	3.4984138	down	unknown	chr13:15947521-15999246_R
A_30_P01031423	0.019505797	3.4926527	down	unknown	chr5:149751979-149808240_F
mmu_2946_PI428990136	0.016914584	3.4344575	down	ncRNA	FR312820
A_30_P01027189	0.04459727	3.2933893	down	unknown	chr10:116814196-116920696_R
A_30_P01022319	0.01807285	3.265757	down	unknown	chr15:100908323-100908645_F
A_30_P01030233	0.013721625	3.1706235	down	unknown	chr9:56646768-56649068_R
A_30_P01025341	0.012241075	3.0579627	down	unknown	chr18:69923410-69965360_R
A_30_P01019875	0.002412234	3.0573971	down	unknown	chr5:110182844-110228345_R
A_30_P01022610	0.03731524	3.0285838	down	unknown	chr1:183142656-183163206_R
mmu_22178_PI428960544	0.0438909	2.9946027	down	ncRNA	FR402142
mmu_10165_PI428990136	0.023415042	2.8444433	down	processed_transcript	ENSMUST00000163396
mmu_21960_PI428960544	0.019531608	2.825909	down	ncRNA	FR283931
A_30_P01019907	0.007787522	2.8112855	down	unknown	chr13:76326228-76332632_F
mmu_32583_PI428960544	0.033733517	2.796274	down	lncRNA	n414371
mmu_5662_PI428960544	0.046895236	2.7944968	down	ncRNA	FR284965
mmu_11372_PI428960544	0.046551984	2.7935345	down	ncRNA	FR097477
mmu_8192_PI428960544	0.03205137	2.768107	down	ncRNA	FR087925
mmu_11164_PI428960544	0.030689355	2.7354522	down	ncRNA	FR185165
A_30_P01027302	0.024274392	2.7353764	down	unknown	chr13:66381200-66410200_F

A_30_P01027503	0.02138378	2.705112	down	unknown	chr11:120041124-120052299_F
A_30_P01030642	0.011392873	2.7048914	down	unknown	chr11:120079199-120130099_R
A_30_P01023528	0.024389397	2.6986594	down	unknown	chr1:138570107-138632310_R
mmu_7989_PI428960544	0.033628024	2.6926668	down	ncRNA	FR301533
A_30_P01028835	0.023176897	2.6676996	down	unknown	chr1:92549499-92550225_R
mmu_25642_PI428960544	9.08E-05	2.6572998	down	ncRNA	FR283504
mmu_5159_PI428960544	0.022221426	2.6336071	down	ncRNA	FR010306
mmu_32238_PI428960544	0.02047917	2.6229687	down	lncRNA	n412060
mmu_14252_PI428960544	0.048546165	2.5963526	down	ncRNA	FR052210
A_30_P01022679	0.017787576	2.5792506	down	unknown	chr3:40439525-40509975_R
mmu_11513_PI428960544	0.028221956	2.5293198	down	ncRNA	FR019981
A_30_P01032830	6.66E-04	2.505982	down	unknown	chr5:137392195-137426421_F
mmu_1097_PI428960544	0.024150344	2.5038707	down	antisense	ENSMUST00000181887
mmu_118_PI428960544	0.04198807	2.4989903	down	lincRNA	ENSMUST00000155173
A_30_P01019182	0.037324745	2.4944587	down	unknown	chr1:138473514-138476054_R
mmu_31315_PI428960544	0.031338718	2.4922397	down	mRNAlike lncRNA	n296710
mmu_10808_PI428960544	0.028998792	2.4909513	down	ncRNA	FR298560
mmu_29726_PI428960544	0.012212492	2.4785485	down	mRNAlike lncRNA	n283487
A_30_P01022534	0.01814157	2.4778404	down	unknown	chr10:62262341-62269593_F
A_30_P01018129	0.031643514	2.4745913	down	unknown	chr19:23068397-23190256_R
A_30_P01033107	0.01159532	2.4407716	down	unknown	chr14:55317199-55317670_R
mmu_6385_PI428960544	0.049094852	2.4406228	down	ncRNA	FR071800
A_30_P01024600	0.037837543	2.436896	down	unknown	chr8:126396600-126407275_R
A_30_P01024783	0.021961328	2.435037	down	unknown	chr10:8606683-8665783_R
mmu_9198_PI428960544	0.015093512	2.410183	down	ncRNA	FR382833
mmu_28093_PI428960544	0.02704069	2.3958325	down	mRNAlike lncRNA	n263541
A_30_P01022501	0.028991783	2.3936949	down	unknown	chr8:119732348-119749198_R
A_30_P01031789	0.035382196	2.3922997	down	unknown	chr1:196445304-196445818_R

mmu_29988_PI428960544	0.011641372	2.384501	down	mRNAlike lncRNA	n287284
mmu_26183_PI428960544	0.03498044	2.3754466	down	ncRNA	FR296035
A_30_P01027704	0.025110798	2.373197	down	unknown	chr12:110003268-110030953_F
mmu_4021_PI428994341	0.004077047	2.3708851	down	ncRNA	uc.427+
A_30_P01030161	0.013195121	2.3652973	down	unknown	chr7:133402766-133415516_R
mmu_21127_PI428960544	0.03303173	2.3562133	down	ncRNA	FR168758
mmu_1624_PI428990136	0.03797109	2.349007	down	ncRNA	FR287008
mmu_2189_PI428960544	0.013430556	2.3424354	down	ncRNA	FR279905
mmu_945_PI428960544	0.037189703	2.3379676	down	lincRNA	ENSMUST00000180590
A_30_P01020655	0.03191105	2.3371174	down	unknown	chr1:92546371-92551596_R
mmu_452_PI428960544	0.00764293	2.3305519	down	processed_transcript	ENSMUST00000125250
A_30_P01027698	0.018198254	2.3292997	down	unknown	chr1:167515313-167516122_F
A_30_P01023619	0.025082035	2.318276	down	unknown	chr12:89519675-89533975_F
A_30_P01026874	0.004059054	2.3156645	down	unknown	chr1:183783306-183834531_F
A_30_P01028311	0.012571103	2.3097718	down	unknown	chr2:158169020-158191295_F
A_30_P01032716	0.014462663	2.3088627	down	unknown	chr9:27148989-27155714_R
mmu_29734_PI428960544	0.0415631	2.2987878	down	mRNAlike lncRNA	n283793
mmu_7865_PI428960544	0.025199296	2.290531	down	ncRNA	FR395572
mmu_34977_PI428960544	0.01206234	2.288283	down	ncRNA	XR_140433.1
mmu_25111_PI428960544	0.025136832	2.2875602	down	ncRNA	FR266999
mmu_3227_PI428990136	0.00117968	2.2856014	down	ncRNA	FR341317
mmu_16003_PI428960544	0.017741848	2.2849877	down	ncRNA	FR101946
A_30_P01017745	0.014221753	2.2778318	down	unknown	chr9:14480441-14483362_F
mmu_18132_PI428960544	0.035464574	2.2764018	down	ncRNA	FR385633
mmu_21041_PI428960544	0.002842722	2.273278	down	ncRNA	FR087537
A_30_P01020505	0.03601559	2.2726173	down	unknown	chr6:52046805-52072744_R
A_30_P01026936	0.030754719	2.2713442	down	unknown	chr12:16873410-16873872_F
A_30_P01024794	0.004521682	2.2709405	down	unknown	chr17:64063362-64066067_R
A_30_P01027781	0.013154849	2.267218	down	unknown	chr7:26212709-26230775_F

A_30_P01023252	0.017467622	2.2656896	down	unknown	chr9:58301467-58323783_F
A_30_P01030037	0.003630241	2.2651737	down	unknown	chr14:25923295-25923893_F
mmu_19340_PI428960544	0.027411953	2.2645314	down	ncRNA	FR057053
mmu_8561_PI428960544	0.03338078	2.263598	down	ncRNA	FR139436
mmu_24851_PI428960544	0.04559214	2.2566936	down	ncRNA	FR285140
mmu_344_PI428960544	0.014360337	2.2562218	down	antisense	ENSMUST00000181563
mmu_22446_PI428960544	0.027249636	2.2557466	down	ncRNA	FR035623
mmu_23140_PI428960544	0.03697652	2.2543762	down	ncRNA	FR049065
mmu_18194_PI428960544	0.025665564	2.252142	down	ncRNA	FR283082
A_30_P01030857	0.043520357	2.2486176	down	unknown	chr15:96597330-96663683_F
mmu_10283_PI428960544	0.036773346	2.2440228	down	ncRNA	FR066795
A_30_P01030971	0.014055186	2.2380555	down	unknown	chr9:121845920-121858120_F
A_30_P01028973	0.047730476	2.2370107	down	unknown	chr9:4260788-4261404_F
mmu_331_PI428960544	0.008400203	2.2346442	down	lincRNA	ENSMUST00000141575
mmu_361_PI428960544	0.017884566	2.2156718	down	unknown	ENSMUST00000174110
mmu_5246_PI428960544	0.041470658	2.212419	down	ncRNA	FR100940
mmu_2724_PI428960544	0.023820087	2.208435	down	ncRNA	FR000724
mmu_10225_PI428960544	3.83E-05	2.2045014	down	ncRNA	FR238898
A_30_P01021046	0.031785145	2.1967378	down	unknown	chr2:132722530-132726802_F
A_30_P01026615	0.010771721	2.1907341	down	unknown	chr9:66976750-67014625_F
mmu_25579_PI428960544	0.04850081	2.1822586	down	ncRNA	FR057875
mmu_27236_PI428960544	0.009006086	2.1809776	down	ncRNA	FR160504
A_30_P01019125	0.00945897	2.1598532	down	unknown	chr7:96640618-96649018_F
mmu_17024_PI428960544	0.015198571	2.1597576	down	ncRNA	FR016598
A_30_P01018441	0.022463292	2.1544268	down	unknown	chr15:32167516-32174417_R
mmu_14135_PI428960544	0.041658662	2.1518717	down	ncRNA	FR230183
mmu_271_PI428994341	0.045557186	2.1491482	down	ncRNA	uc.31+
mmu_10275_PI428960544	0.020131843	2.1467834	down	ncRNA	FR152607
A_30_P01020067	0.004916142	2.138676	down	unknown	chr14:21621875-21638625_R
mmu_26090_PI428960544	0.019206157	2.1361675	down	ncRNA	FR215734
A_30_P01020455	0.034770347	2.1358068	down	unknown	chr6:86425510-86450160_F
mmu_17900_PI428960544	0.023148267	2.135754	down	ncRNA	FR380314
A_30_P01017878	0.007169262	2.135222	down	unknown	chr5:35893908-35896613_R
mmu_15990_PI428960544	0.047904074	2.1343312	down	ncRNA	FR338634

mmu_1364_PI428960544	0.013056339	2.133426	down	misc_RNA	ENSMUST00000157205
A_30_P01019217	0.016911205	2.1285634	down	unknown	chr13:98513868-98517366_R
mmu_28939_PI428960544	0.013129035	2.1280828	down	mRNAlike lncRNA	n272641
mmu_19062_PI428960544	0.016851876	2.126122	down	ncRNA	FR121241
A_30_P01031934	0.037664108	2.1228049	down	unknown	chr4:35106125-35127700_F
A_30_P01026357	0.019616615	2.1220376	down	unknown	chr4:116790373-116797823_F
mmu_585_PI428980890	0.044789713	2.1214995	down	ncRNA	FR001084
mmu_26351_PI428960544	0.01881121	2.118441	down	ncRNA	FR064590
mmu_12315_PI428960544	0.02310125	2.1174073	down	ncRNA	FR054476
A_30_P01025047	0.002430505	2.1158981	down	unknown	chrX:165792277-165807952_R
A_30_P01021835	0.03904252	2.114595	down	unknown	chr7:91558475-91733625_R
mmu_15351_PI428960544	0.017821867	2.114136	down	ncRNA	FR087516
A_30_P01027969	0.048478946	2.1117315	down	unknown	chr16:4871449-4874336_R
A_30_P01018820	0.009623749	2.1096225	down	unknown	chr9:27148989-27155714_R
A_30_P01032833	0.012419155	2.1093123	down	unknown	chr2:72818943-72826893_R
mmu_771_PI428994341	0.039914522	2.1060395	down	ncRNA	uc.83+
A_30_P01032425	0.015645128	2.1054952	down	unknown	chr8:26345571-26346095_R
A_30_P01022508	0.012299637	2.104819	down	unknown	chr5:54053882-54054174_F
A_30_P01030360	0.028412344	2.0962455	down	unknown	chr11:33414450-33450750_F
A_30_P01031798	0.005420569	2.0944998	down	unknown	chr7:31709550-31723025_F
mmu_27955_PI428960544	0.010624005	2.0937715	down	ncRNA	FR065962
A_30_P01031642	0.044676058	2.0923152	down	unknown	chr17:29374758-29387733_R
A_30_P01027155	0.044732917	2.089499	down	unknown	chr9:68349343-68411643_R
A_30_P01019679	0.016120747	2.086273	down	unknown	chr6:37630192-37682692_R
A_30_P01033372	3.40E-04	2.08536	down	unknown	chr10:24269165-24276690_F
mmu_2185_PI428960544	0.03831714	2.0796025	down	ncRNA	FR302003
A_30_P01018664	0.026820624	2.07102	down	unknown	chr3:35782698-35789932_R

A_30_P01018171	0.027798342	2.06712	down	unknown	chr11:69184157-69201732_R
mmu_19439_PI428960544	0.014271176	2.0669875	down	ncRNA	FR061730
mmu_5787_PI428960544	0.01091862	2.0647528	down	ncRNA	FR187810
A_30_P01022455	0.041985184	2.0641687	down	unknown	chr9:27155685-27156100_F
mmu_12637_PI428960544	0.026744938	2.062086	down	ncRNA	FR162622
mmu_3104_PI428960544	0.0402705	2.0592556	down	ncRNA	FR011959
mmu_17416_PI428960544	4.00E-05	2.0587	down	ncRNA	FR184042
A_30_P01025376	0.003237647	2.0559545	down	unknown	chr2:71576218-71603818_R
mmu_22751_PI428960544	0.013097336	2.0553932	down	ncRNA	FR121323
mmu_24455_PI428960544	0.002966158	2.0541143	down	ncRNA	FR219193
mmu_1125_PI428960544	0.005725785	2.0520942	down	antisense	ENSMUST00000134129
A_30_P01021538	0.021673927	2.0518541	down	unknown	chr2:151992806-152025456_F
mmu_9752_PI428960544	0.006350653	2.0506284	down	ncRNA	FR055065
A_30_P01018439	0.022327261	2.0498476	down	unknown	chr3:93582250-93592950_F
mmu_334_PI428960544	0.023119668	2.0493479	down	lincRNA	ENSMUST00000132130
A_30_P01031525	0.018976577	2.0490186	down	unknown	chr7:80762708-80763274_R
mmu_345_PI428960544	0.003001115	2.0465593	down	lincRNA	ENSMUST00000130677
A_30_P01026685	0.024701972	2.0422573	down	unknown	chr11:116936834-116948799_R
mmu_33766_PI428960544	0.042725112	2.0394514	down	ncRNA	XR_141342.2
mmu_12081_PI428960544	0.010358787	2.0362997	down	ncRNA	FR365932
A_30_P01023983	0.044055622	2.0273085	down	unknown	chr16:59557120-59602070_R
mmu_34199_PI428960544	0.017124603	2.0272737	down	ncRNA	XR_141927.1
A_30_P01032892	0.049760524	2.024637	down	unknown	chr8:129102060-129109035_F
A_30_P01031061	0.020148082	2.021439	down	unknown	chr1:183783306-183834531_F
mmu_18399_PI428960544	0.010291548	2.0208619	down	ncRNA	FR047247
mmu_17324_PI428960544	0.040216777	2.020591	down	ncRNA	FR129526
A_30_P01025347	0.041025043	2.0202909	down	unknown	chr8:108153471-108159672_F
mmu_4199_PI428960544	0.01466605	2.01822	down	ncRNA	FR177295
A_30_P01030661	0.005499327	2.0165284	down	unknown	chr15:60732209-60732822_R
mmu_18525_PI428960544	0.027496647	2.0165238	down	ncRNA	FR029778
mmu_9585_PI428960544	0.015517354	2.0138235	down	ncRNA	FR237950
mmu_15016_PI428960544	0.005695541	2.0123365	down	ncRNA	FR003529
mmu_2856_PI428960544	0.033858698	2.0116575	down	ncRNA	FR294279

A_30_P01032356	0.023731638	2.0089705	down	unknown	chr14:105497388-105511063_F
mmu_32101_PI428960544	0.020649977	2.0072768	down	lincRNA	n344241
A_30_P01023152	0.048081096	2.0023656	down	unknown	chr17:10057283-10075014_F
mmu_8619_PI428990136	0.026401438	2.001239	down	ncRNA	NR_102339.1
A_30_P01019636	0.015422435	2.0004997	down	unknown	chr1:183783306-183834531_F
A_30_P01029676	0.02586736	2.0004468	down	unknown	chr13:48576557-48581632_F

Table-S2: Significantly and differentially expressed mRNAs in high-fat and normal diet ApoE(-/-) mice

ProbeName	p	FC (abs)	Regulation	Entrez.Gene.ID	Symbol
A_55_P2085142	0.047909692	12.779388	up	20750	Spp1
A_55_P2099677	0.039917916	10.249345	up	14725	Lrp2
A_55_P2126167	0.038570475	8.750357	up	234155	Mboat4
A_52_P333097	1.15145E-05	6.273958	up	171166	Mcoln3
A_55_P2154933	0.040734425	6.209726	up	239849	Cd200r4
A_55_P1968078	0.0309128	5.3137326	up	269109	Dpp10
A_52_P622667	0.04698343	5.2924924	up	75300	4930548F15Rik
A_51_P304478	0.028914226	5.2438664	up	270028	Fam155a
A_51_P363187	0.041922424	5.223882	up	14825	Cxcl1
A_55_P2350710	0.03724817	5.1480927	up	unknown	unknown
A_52_P258617	0.005367049	5.1299367	up	17079	Cd180
A_55_P2185000	0.015237055	4.998701	up	72065	Rap2c
A_55_P1953758	0.01608326	4.9926405	up	230779	Serinc2
A_51_P241068	0.011512839	4.8305373	up	56811	Dkk2
A_51_P454873	0.008715192	4.591792	up	109648	Npy
A_51_P461319	0.010853741	4.5736413	up	67092	Gatm
A_51_P128075	0.00890484	4.3971786	up	69301	Tescl
A_51_P246653	0.047535725	4.3486605	up	56644	Clec7a
A_55_P2230484	0.029416125	4.333681	up	75958	5033403F01Rik
A_55_P2067583	0.018470999	4.32177	up	12514	Cd68
A_55_P1984262	0.04379719	4.245508	up	79455	Pdcl2
A_55_P1957353	0.008730723	4.219927	up	unknown	unknown
A_51_P275454	0.030209051	4.218605	up	20128	Trim30a
A_55_P2053532	0.020991549	4.1950393	up	242819	Rundc3b
A_55_P2046693	0.00312842	4.0943704	up	11740	Slc25a5
A_51_P468260	0.003079893	4.04279	up	20203	S100b
A_55_P2094262	0.02177802	4.0096984	up	21990	Tph1
A_51_P133884	0.023813922	3.9952018	up	170786	Cd209a
A_52_P480044	0.000556209	3.9528449	up	667597	BC023105
A_55_P2055063	0.03961769	3.9309678	up	68995	Mcts1
A_55_P2317730	0.048966106	3.826572	up	70286	2310067P03Rik
A_51_P279693	0.005365545	3.8041263	up	13076	Cyp1a1
A_55_P2057881	0.032142926	3.7899761	up	71617	9130011E15Rik
A_51_P170562	0.003372009	3.7382753	up	71756	Cpn2
A_55_P1956897	0.043850403	3.7054188	up	574081	Defb46
A_55_P1984815	0.03947168	3.682672	up	241727	Snph
A_55_P2039541	0.0434416	3.5888941	up	76117	Arhgap15
A_51_P164203	0.04683555	3.553887	up	56520	Nme4
A_51_P209327	0.003861398	3.5199928	up	30878	Apln

A_52_P623511	0.035699442	3.4919183	up	100042512	Gm3877
A_55_P2006499	0.02531045	3.4900794	up	26381	Esrrg
A_55_P2472435	4.65072E-05	3.441332	up	55932	Gbp3
A_52_P300376	0.025017247	3.4256492	up	213742	Xist
A_55_P2069197	0.045313142	3.347283	up	387352	Tas2r125
A_55_P2126557	0.032374784	3.2908635	up	unknown	unknown
A_51_P155723	0.04013059	3.2466013	up	27405	Abcg3
A_55_P1985239	0.01165134	3.206714	up	69352	Necab1
A_52_P35072	0.024097955	3.1915276	up	668408	1700084K02Rik
A_51_P103222	0.040311474	3.180983	up	72027	Slc39a4
A_55_P2077368	0.02412998	3.1513274	up	319446	Dpep2
A_55_P2106440	0.042023487	3.1331503	up	unknown	unknown
A_55_P2119263	0.017465679	3.1298544	up	unknown	unknown
A_55_P2009375	0.008165068	3.1272662	up	18557	Cdk18
A_55_P2035623	0.032914773	3.1226034	up	243963	Zfp473
A_51_P210956	0.011301947	3.0950074	up	22329	Vcam1
A_51_P286748	0.019359628	3.063156	up	20378	Frzb
A_55_P1962400	0.016647352	3.0623827	up	16181	Il1rn
A_55_P2072816	0.019117346	3.007732	up	245684	Cnksr2
A_55_P2241553	0.047021598	2.9521923	up	100689704	9330133O14Rik
A_51_P236267	0.03945416	2.942745	up	20452	St8sia4
A_55_P2170953	0.045423012	2.9404774	up	unknown	unknown
A_55_P2063654	0.02876305	2.8701584	up	381530	Mup20
A_55_P2296641	0.031239254	2.8638194	up	74672	4930449A18Rik
A_51_P302181	0.002274608	2.8605325	up	83813	Tnk1
A_52_P520495	0.017399061	2.8501952	up	22329	Vcam1
A_55_P1962766	0.024083316	2.8326273	up	277743	Fam131c
A_55_P2004447	0.021743901	2.828154	up	234199	Fgl1
A_55_P1955931	0.03226132	2.8075843	up	73095	Slc25a42
A_55_P1964247	0.038869355	2.7931912	up	unknown	unknown
A_66_P100284	0.047623623	2.73436	up	100039736	A430060F13Rik
A_55_P2026139	0.012940364	2.7317605	up	381680	Nxpe5
A_66_P132825	0.03295673	2.69237	up	unknown	unknown
A_55_P2128068	0.026557067	2.6916502	up	unknown	unknown
A_51_P196925	0.03813867	2.680274	up	20312	Cx3cl1
A_51_P354744	0.005033741	2.6524894	up	226139	Cox15
A_55_P2004168	0.0333444	2.603626	up	unknown	unknown
A_51_P239166	0.040161207	2.6027062	up	56622	Adam21
A_66_P111090	0.026383657	2.5926566	up	319229	Sctr
A_55_P1984243	0.016010102	2.5654674	up	17948	Naip2
A_55_P2312532	0.006061391	2.562299	up	101769	AU023617
A_51_P346938	0.01721557	2.559332	up	76905	Lrg1
A_52_P282741	0.022040052	2.5564668	up	20970	Sdc3

A_51_P364485	0.004587128	2.5560002	up	21928	Tnfaip2
A_55_P2012989	0.04921939	2.544938	up	75345	Slamf7
A_51_P338443	0.014924821	2.541999	up	57875	Angptl4
A_55_P1973279	0.018423704	2.5357826	up	unknown	unknown
A_55_P2092310	0.010213793	2.5322466	up	236539	Phgdh
A_55_P2096917	0.04812036	2.532236	up	381269	Mreg
A_55_P1984416	0.014690093	2.5270193	up	unknown	unknown
A_55_P2112642	0.015213939	2.5263479	up	22095	Tshr
A_55_P2037613	0.03113549	2.5138779	up	238317	Elmsan1
A_55_P1968245	0.02284636	2.5138156	up	14325	Ftl1
A_55_P1965368	0.031910896	2.507902	up	68964	Ctc1
A_55_P1972582	0.04831336	2.5078235	up	240921	Gm4955
A_55_P2015169	0.02385201	2.5064943	up	unknown	unknown
A_52_P547662	0.024957659	2.5007973	up	18441	P2ry1
A_55_P2033650	0.004920321	2.4731076	up	12896	Cpt2
A_52_P52618	0.022852102	2.4707537	up	12983	Csf2rb
A_55_P2021659	0.013369442	2.4625342	up	74610	Abcb8
A_55_P1980426	0.0328923	2.4310877	up	18111	Nnat
A_55_P1959863	0.028833413	2.4274862	up	unknown	unknown
A_55_P1989767	0.04968325	2.4261076	up	100042656	Gm3952
A_51_P405638	0.012202355	2.4243295	up	unknown	unknown
A_55_P2132626	0.021819005	2.4219851	up	unknown	unknown
A_66_P122781	0.01115184	2.420465	up	268319	BC025920
A_55_P2066116	0.002826603	2.411398	up	12051	Bcl3
A_51_P304170	0.008278701	2.409842	up	67775	Rtp4
A_52_P623226	0.038927548	2.3968885	up	14344	Fut2
A_55_P1973995	0.026997153	2.3954573	up	627427	Gm6756
A_52_P97572	0.01776409	2.3884819	up	23796	Aplnr
A_55_P2018412	0.026621796	2.3882632	up	320558	Sycp2
A_51_P514029	0.042780545	2.381031	up	17228	Cma1
A_65_P17218	0.015489441	2.3785565	up	100040464	Mndal
A_55_P2273656	0.02539133	2.3694644	up	unknown	unknown
A_51_P369862	0.03298347	2.3680797	up	320024	Nceh1
A_55_P2115981	0.001495549	2.3680742	up	27084	Xlr5c
A_55_P1960999	0.033409998	2.3661308	up	18703	Pigr
A_55_P2099750	0.01373058	2.3596137	up	unknown	unknown
A_55_P2273929	0.04164725	2.348326	up	330108	Gm20558
A_55_P2058812	0.036225017	2.3447452	up	67030	Fancl
A_55_P1980237	0.03506632	2.33826	up	102632	Acad11
A_55_P2033780	0.031219523	2.3373573	up	14077	Fabp3
A_66_P119034	0.023960907	2.3339715	up	27226	Pla2g7
A_52_P90363	0.009038393	2.3280945	up	76933	Ifi27l2a
A_55_P2148333	0.049886998	2.325698	up	69944	2810021J22Rik

A_51_P128463	0.001507823	2.3247635	up	72690	Grrp1
A_55_P2161400	0.026398655	2.323082	up	319229	Sctr
A_51_P477458	0.019334232	2.3133683	up	14804	Grid2
A_55_P2144456	0.036116224	2.3094978	up	225888	Suv420h1
A_55_P1975370	0.01679943	2.304937	up	11812	Apoc1
A_66_P114784	0.011915872	2.2910724	up	27226	Pla2g7
A_52_P93910	0.018065158	2.289967	up	18187	Nrp2
A_55_P1965882	0.044359222	2.2788634	up	67133	Gp2
A_55_P2000543	0.03023971	2.2699194	up	69142	Cd209f
A_55_P2041539	0.024599515	2.267068	up	72084	Pigx
A_55_P1983523	0.002685566	2.2605038	up	217305	Cd300ld
A_55_P2090835	0.012910369	2.2516289	up	668929	Rad21l
A_52_P116264	0.035204124	2.2509747	up	76187	Adhfe1
A_51_P161636	0.010151357	2.2429368	up	385643	Kng2
A_55_P2084283	0.023545582	2.2344277	up	100039952	Gm581
A_55_P2081462	0.04366323	2.2329564	up	unknown	unknown
A_55_P2114143	0.02245103	2.228168	up	66832	Rsph3a
A_55_P1959938	0.001651314	2.2166774	up	29863	Pde7b
A_55_P2095311	0.026882017	2.208151	up	546644	Ly6g
A_51_P178772	0.022451349	2.2010272	up	234564	Ces1f
A_51_P359636	0.007052681	2.2003767	up	19039	Lgals3bp
A_66_P100496	0.032784443	2.1986833	up	73526	Speer4b
A_55_P2151116	0.026731754	2.1941268	up	216001	Micu1
A_55_P2141699	0.02525764	2.1892893	up	67702	Rnf149
A_55_P1978927	0.011771943	2.1854382	up	117600	Srgap1
A_55_P2069480	0.022922821	2.1837528	up	unknown	unknown
A_55_P1966833	0.003900858	2.1811943	up	327959	Xaf1
A_55_P2026614	0.037938036	2.1706357	up	unknown	unknown
A_55_P2140745	0.029643333	2.1668842	up	unknown	unknown
A_51_P214269	0.034306634	2.161458	up	13835	Epha1
A_52_P198898	0.009658755	2.161073	up	320825	Samd5
A_55_P2110743	0.04241234	2.1587713	up	393082	Mettl7a2
A_55_P2004507	0.030344062	2.1565373	up	15950	Ifi203
A_55_P1994773	0.020364463	2.1558022	up	100042784	Gm16381
A_51_P207153	0.000938668	2.1557894	up	73737	1110008P14Rik
A_66_P122086	0.005391482	2.1554174	up	105892	9030619P08Rik
A_52_P183038	0.007086929	2.1539397	up	23917	Impdh1
A_51_P211854	0.021653045	2.1474924	up	20344	Selp
A_55_P2152304	0.01247126	2.1405215	up	71679	Atp5h
A_66_P127070	0.0321974	2.13673	up	14563	Gdf5
A_55_P2074796	0.003146816	2.1320302	up	12580	Cdkn2c
A_55_P2119613	0.012425814	2.1315558	up	71037	Prss55
A_55_P2101021	0.035336614	2.1289787	up	100470	Lao1

A_55_P2023912	0.043699194	2.124647	up	unknown	unknown
A_55_P2029945	0.019290447	2.1240935	up	unknown	unknown
A_55_P2035772	0.006187427	2.1191614	up	26565	Pla2g10
A_51_P386503	0.048231937	2.1185246	up	67477	Abhd15
A_52_P223809	0.033739008	2.117534	up	80861	Dhx58
A_55_P2073432	0.03358394	2.1129231	up	20890	Wnt8a
A_55_P1998601	0.041798722	2.1128073	up	228993	Slc17a9
A_55_P2022123	0.036919165	2.1082647	up	380709	Spata22
A_55_P2158592	0.002482493	2.1008945	up	320832	Sirpb1a
A_55_P2052485	0.020539246	2.0909472	up	234395	Ushbp1
A_51_P256202	0.011073636	2.0902686	up	64138	Ctsz
A_52_P484956	0.006802814	2.0882976	up	18111	Nnat
A_55_P1953063	0.006051819	2.0864136	up	15528	Hspe1
A_55_P2055087	0.04376721	2.084407	up	19088	Prkar2b
A_55_P2075692	0.011896908	2.0833895	up	226025	Trpm3
A_51_P309534	0.017590698	2.0742583	up	56273	Pex14
A_55_P1984168	0.018851578	2.070067	up	12363	Casp4
A_55_P1973723	0.019624356	2.0664158	up	66841	Etfdh
A_55_P2015687	0.037229303	2.0660286	up	219132	Phf11d
A_55_P1991773	0.039708514	2.0593126	up	54167	Icos
A_55_P1985850	0.04247972	2.0563192	up	21857	Timp1
A_55_P1969251	0.044733945	2.0445273	up	54607	Socs6
A_55_P2010048	0.044077646	2.0437882	up	100040352	2810416G20Rik
A_55_P2133370	0.03651179	2.0423667	up	100043728	Gm4610
A_51_P269084	0.015994722	2.0413504	up	103172	Chchd10
A_55_P2048912	0.012918615	2.035696	up	230787	Themis2
A_55_P2124346	0.0379883	2.035425	up	117198	Ivns1abp
A_55_P2364768	0.014896594	2.0349605	up	17223	Mcpt-ps1
A_52_P609109	0.023460018	2.0323677	up	71795	Pitpnc1
A_55_P2071751	0.046933923	2.0314403	up	unknown	unknown
A_55_P1997766	0.007344226	2.030851	up	11433	Acp5
A_55_P2044547	0.03059977	2.0296483	up	100040352	2810416G20Rik
A_55_P2281891	0.036241744	2.0277634	up	242509	Bnc2
A_55_P2095498	0.013830009	2.0269701	up	16528	Kcnk4
A_51_P464918	0.024659347	2.0207765	up	54483	Mefv
A_51_P483483	0.022292726	2.0194538	up	unknown	unknown
A_52_P584335	0.03732438	2.017755	up	14275	Folr1
A_52_P121960	0.01788441	2.0136852	up	unknown	unknown
A_55_P2032768	0.036759336	2.009535	up	unknown	unknown
A_55_P2118684	0.01620513	2.009426	up	269951	Idh2
A_51_P377452	0.03724348	2.0084043	up	17972	Ncf4
A_52_P475738	0.035908252	2.0057378	up	75805	Nln
A_51_P329949	0.002439679	2.001273	up	58909	Fam13a

A_51_P202331	0.031255517	7.6572948	down	18843	Bpifa1
A_55_P2079269	0.00440951	5.486639	down	unknown	unknown
A_55_P1994739	0.014814098	4.6729183	down	unknown	unknown
A_55_P2103001	0.006502333	4.636288	down	unknown	unknown
A_55_P1995580	0.025260188	4.2711887	down	17232	Mcpt9
A_51_P346565	0.014681984	4.1693983	down	237979	Sdk2
A_55_P2126441	0.033518147	4.042017	down	75657	Speer4a
A_55_P2243162	0.016519992	3.6924362	down	unknown	unknown
A_55_P2191303	0.001593046	3.6259832	down	unknown	unknown
A_55_P2114959	0.043359816	3.4325883	down	100038864	Btnl1
A_55_P2099881	0.030040398	3.412374	down	100038480	Gm10461
A_52_P117408	0.025265183	3.3665097	down	21819	Tg
A_55_P2066214	0.02178445	3.360974	down	unknown	unknown
A_55_P2006385	0.026010714	3.3271277	down	545475	Defb28
A_51_P133638	0.039128315	3.167933	down	67289	3110021A11Rik
A_55_P1984401	0.018641414	3.1141481	down	210274	Shank2
A_55_P2011096	0.023785863	3.0995102	down	76758	Gsdma2
A_55_P1977451	0.030442294	2.9830418	down	unknown	unknown
A_55_P2119917	0.031079613	2.9718652	down	22781	Ikzf4
A_52_P468683	3.27028E-05	2.9708135	down	235599	6430571L13Rik
A_55_P1955602	0.045019343	2.945545	down	unknown	unknown
A_55_P2126528	0.025554769	2.9330158	down	unknown	unknown
A_55_P2173039	0.023144877	2.9307976	down	unknown	unknown
A_55_P1963221	0.009599702	2.8623981	down	unknown	unknown
A_55_P2133943	0.03997427	2.8491256	down	101056064	LOC101056066
A_51_P201174	0.015308277	2.7984986	down	67717	Lipf
A_55_P2044232	0.024074197	2.7781916	down	241877	Slc10a5
A_55_P2410463	0.000292941	2.7454906	down	98087	C87487
A_55_P2186978	0.006922412	2.7305107	down	unknown	unknown
A_55_P2058040	0.00017882	2.6976464	down	unknown	unknown
A_55_P2060897	0.039009877	2.6701639	down	unknown	unknown
A_52_P88007	0.021873903	2.656091	down	240332	Slc6a7
A_55_P2055970	0.04857904	2.649529	down	unknown	unknown
A_55_P2145224	0.047457587	2.6447887	down	unknown	unknown
A_55_P2065510	0.04002854	2.6179883	down	unknown	unknown
A_55_P2090265	0.006611924	2.5945392	down	319555	Nwd1
A_55_P1968799	0.021759612	2.5923593	down	69664	Krtap1-5
A_55_P2108221	0.033530883	2.5824623	down	unknown	unknown
A_55_P2335878	0.01586151	2.572494	down	77813	A930012N16Rik
A_52_P213483	0.021483103	2.5461378	down	619994	Ighv1-77
A_51_P245202	0.005832666	2.5343828	down	unknown	unknown
A_55_P2053923	0.0016555	2.5257208	down	19329	Rab17
A_51_P483576	0.021697117	2.5185776	down	unknown	unknown

A_55_P2166618	0.017512461	2.5181432	down	unknown	unknown
A_55_P2088113	0.012783063	2.5147479	down	100038592	Gm16287
A_52_P662711	0.044325747	2.511018	down	69787	Anxa13
A_52_P534001	0.021442037	2.507867	down	74708	Pih1d3
A_55_P1958227	0.037325732	2.4807858	down	unknown	unknown
A_51_P230734	0.016764158	2.4642513	down	142681	Slc34a3
A_55_P1988994	0.025150066	2.458563	down	unknown	unknown
A_52_P270429	0.044121213	2.445454	down	69134	Fam25c
A_55_P2031851	0.021446884	2.4425776	down	unknown	unknown
A_55_P2213214	0.009114099	2.4409592	down	100126240	A030001D20Rik
A_66_P124420	0.006866672	2.423194	down	unknown	unknown
A_55_P2003911	0.013248686	2.4227715	down	unknown	unknown
A_55_P2255206	0.000331767	2.415714	down	319782	A730021G18Rik
A_55_P2116853	0.041127652	2.4116418	down	78783	Brpf1
A_55_P2047043	0.018602936	2.4042046	down	228785	Mylk2
A_52_P374983	0.02728542	2.4030964	down	320377	9330175E14Rik
A_55_P1985814	0.024032002	2.39653	down	unknown	unknown
A_55_P1957144	0.024472598	2.3762164	down	217169	Tns4
A_52_P384036	0.018876197	2.375959	down	271813	Agbl2
A_55_P2179884	0.03272248	2.3512454	down	unknown	unknown
A_51_P468073	0.043852445	2.3508759	down	14598	Ggt1
A_52_P442169	0.020081572	2.3049946	down	12217	Bsn
A_55_P2148726	0.03631526	2.2980077	down	unknown	unknown
A_55_P2086193	0.027911872	2.2975194	down	60613	Kcnq4
A_66_P130241	0.009273455	2.293709	down	13358	Slc25a1
A_55_P2009430	0.03612608	2.2680302	down	101056104	LOC101056101
A_51_P496720	0.03713821	2.2667727	down	54427	Dnmt3l
A_55_P2300421	0.032299157	2.2643816	down	319875	Tmprss11bnl
A_55_P2002954	0.000458747	2.2522793	down	unknown	unknown
A_51_P230142	0.044258393	2.251463	down	68243	A930018P22Rik
A_55_P2047196	0.000153995	2.2453992	down	75641	1700029I15Rik
A_55_P2169431	0.025901236	2.2400284	down	unknown	unknown
A_55_P2072881	0.034041513	2.2375202	down	unknown	unknown
A_55_P1989076	0.046067115	2.231466	down	73102	Slc22a23
A_51_P340170	0.020352589	2.2196074	down	237313	Il20ra
A_55_P2032458	0.019200377	2.217785	down	628919	Gm6934
A_55_P2154994	0.029733976	2.2170353	down	77917	A030014E15Rik
A_52_P567200	0.026564317	2.206506	down	237178	Ppef1
A_55_P2076826	0.007595602	2.201091	down	320189	9430076C15Rik
A_55_P2037643	0.019085467	2.185478	down	unknown	unknown
A_55_P1976574	0.049756996	2.183924	down	50874	Tmod4
A_55_P1983253	0.018878039	2.1786005	down	unknown	unknown
A_51_P197680	0.021271529	2.174422	down	70515	5730407I07Rik

A_52_P684179	0.017711962	2.1713197	down	66779	4933432I09Rik
A_52_P330111	0.028130854	2.1640189	down	75136	Rsph10b2
A_55_P2084855	0.015315488	2.1618073	down	381922	D830044I16Rik
A_55_P2127194	0.034788437	2.1586893	down	unknown	unknown
A_55_P2082403	0.008919989	2.1568027	down	20473	Six3
A_52_P22781	0.019874422	2.1557739	down	330788	Zfp866
A_52_P105765	0.001277731	2.1536753	down	240505	Cdc42bpg
A_55_P2089472	0.048422836	2.1494179	down	75656	1700020A23Rik
A_55_P2344804	0.019417379	2.1428874	down	97028	C87490
A_55_P2020090	0.032266323	2.142365	down	245631	Mum1l1
A_55_P2142990	0.030027848	2.1320937	down	100503312	Pifo
A_66_P130730	0.009548812	2.1169531	down	620419	Zfp963
A_55_P2056580	0.033821307	2.116641	down	unknown	unknown
A_55_P1954061	0.011704716	2.114691	down	18190	Nrxn2
A_55_P1957867	0.016407108	2.1137652	down	100041144	Gm3161
A_51_P209818	0.008810632	2.113711	down	19152	Prtn3
A_55_P2056443	0.020867284	2.1130006	down	100182	Akna
A_52_P650037	0.005532681	2.1103785	down	243743	Plxna4
A_55_P2117092	0.04277561	2.106562	down	unknown	unknown
A_55_P2075278	0.01667714	2.096321	down	71131	Zfp689
A_55_P2086198	0.019227259	2.0916715	down	60613	Kcnq4
A_55_P2088497	0.029133176	2.0901682	down	641235	LOC641235
A_55_P2317346	0.039405257	2.088493	down	unknown	unknown
A_51_P414879	0.040188473	2.088178	down	unknown	unknown
A_55_P2071646	0.005754638	2.0858612	down	unknown	unknown
A_55_P1986665	0.008474236	2.0855317	down	258493	Olfr319
A_66_P132515	0.002603067	2.0829477	down	66371	Chmp4c
A_55_P2006629	0.04670997	2.0792224	down	unknown	unknown
A_55_P2005898	0.011413094	2.0776815	down	unknown	unknown
A_55_P2243237	0.008989604	2.0688028	down	102975	AU015621
A_66_P136778	0.046309486	2.0681882	down	332110	Mapk15
A_55_P2013559	0.001842453	2.065804	down	13483	Dpp6
A_55_P2131672	0.010677956	2.0636127	down	320878	Mical2
A_66_P126848	0.009278496	2.0605028	down	434729	Gm5635
A_55_P2177463	0.001163216	2.059919	down	434198	B130024G19Rik
A_55_P2275999	0.026746618	2.0593078	down	100504352	C78197
A_51_P302520	0.004849006	2.054695	down	17929	Myom1
A_55_P2000369	0.016052974	2.0531092	down	11829	Aqp4
A_55_P2221107	0.028678203	2.0513802	down	77703	9230106L01Rik
A_55_P2133027	0.036322895	2.0475428	down	67703	Kirrel3
A_66_P124776	0.018997245	2.04497	down	101056432	LOC101056428
A_55_P2096602	0.03691907	2.0443246	down	78829	Tsc22d4
A_55_P1979477	0.040414736	2.0403657	down	unknown	unknown

A_55_P2122300	0.025178948	2.0379837	down	66935	Cir1
A_51_P175580	0.03223037	2.0339873	down	60599	Trp53inp1
A_55_P2410626	0.01283084	2.0325806	down	319285	A430061O12Rik
A_52_P223127	0.015601805	2.0304494	down	118449	Synpo2
A_55_P1975937	0.012108337	2.0295537	down	unknown	unknown
A_55_P1966239	0.021343568	2.0288959	down	27205	Podxl
A_55_P2066894	0.012908251	2.0206168	down	unknown	unknown
A_55_P2040227	0.014465137	2.0175316	down	627626	Ptchd4
A_65_P16483	0.009964675	2.016442	down	59020	Pdzk1
A_55_P1953013	0.039945114	2.014867	down	232035	Ccser1
A_55_P2042341	0.012612159	2.0137198	down	258768	Olfr1189
A_55_P1993089	0.044883043	2.0101953	down	100043680	Gm4583
A_55_P2081652	0.019330632	2.00956	down	232836	Galp
A_55_P1979768	0.002832676	2.0093908	down	15405	Hoxa9
A_55_P2158251	0.028082196	2.0065305	down	14807	Grik3
A_55_P2045002	0.013045222	2.0054398	down	13859	Eps15l1
A_51_P105887	0.00938716	2.0045881	down	240754	Lax1

Table-S3 Top 500 most correlated LncRNAs and mRNAs

No	lncRNA	correlated mRNA	*p* -Value	correlation
1	chr18_5813291_5813708_R	Kirrel3	0.000100214	-0.9918151
2	chr4_88745771_88776323_F	Mylk2	0.000100263	0.99181313
3	uc.427+	Dpp6	0.000100461	0.99180505
4	NR_073368.1	Prtn3	0.000100477	-0.9918044
5	FR155040	Mboat4	0.000100553	0.9918013
6	FR301809	LOC101056101	0.000100615	-0.9917987
7	NR_045899.1	Icos	0.000100615	0.99179873
8	FR374009	Suv420h1	0.000100812	0.99179072
9	FR043548	Gm16381	0.000100817	0.99179052
10	n290305	Tescl	0.000100916	0.99178648
11	chr7_133402766_133415516_R	Rsph3a	0.000100953	-0.991785
12	FR199374	1700084K02Rik	0.000101151	0.99177689
13	n412060	D830044I16Rik	0.000101217	0.99177422
14	FR212572	Zfp689	0.000101245	0.99177308
15	chr1_183142656_183163206_R	Micu1	0.000101304	-0.9917707
16	chr17_10057283_10075014_F	Pigr	0.000101329	-0.9917697
17	chr9_67414463_67483185_R	Zfp689	0.000101623	0.9917577
18	chr1_75075500_75102200_R	Abcg3	0.000101638	0.99175712
19	FR171433	Brpf1	0.00010167	-0.9917558
20	FR268824	Bnc2	0.000101794	0.99175075
21	FR279620	LOC101056066	0.000101824	-0.9917496
22	FR114000	Defb46	0.000101832	0.99174923
23	chr1_138473514_138476054_R	A930018P22Rik	0.000101967	0.99174375
24	NR_033641.2	Bnc2	0.000102028	0.99174129
25	FR392828	Mreg	0.000102058	0.99174006
26	Gomafu	Pih1d3	0.000102072	-0.9917395
27	FR298560	Suv420h1	0.000102097	-0.9917385
28	FR120704	A430060F13Rik	0.000102291	0.99173063
29	FR035623	Btnl1	0.000102343	0.99172851
30	FR042390	Slc6a7	0.000102381	-0.991727
31	FR215148	Mettl7a2	0.000102551	0.99172013
32	chr10_18036490_18059965_F	Pla2g10	0.00010256	0.99171976
33	FR312820	Agbl2	0.000102603	0.99171799
34	FR168758	Fam25c	0.000102923	0.99170508
35	NR_045899.1	Ushbp1	0.000102956	0.99170377
36	FR168142	Snph	0.00010296	0.99170361
37	chr13_48576557_48581632_F	Btnl1	0.00010297	0.99170317
38	FR298560	9330175E14Rik	0.00010297	0.9917032
39	FR152607	Tmprss11bnl	0.000103012	0.99170149
40	chr3_35782698_35789932_R	Ighv1-77	0.000103415	0.99168525
41	FR139436	Grik3	0.000103494	0.99168206

42	n296710	LOC101056428	0.000103523	0.99168093
43	FR029778	Kcnq4	0.000103559	0.99167947
44	chr16_59557120_59602070_R	Nme4	0.000103605	-0.9916776
45	FR136228	Fam155a	0.000103624	0.99167684
46	n413982	Ikzf4	0.000103643	-0.9916761
47	FR185165	Ikzf4	0.000103769	0.99167103
48	FR212572	Agbl2	0.000103848	0.99166785
49	FR052210	5033403F01Rik	0.000104026	-0.9916607
50	n412060	Dnmt3l	0.00010443	0.9916445
51	FR155648	Kcnk4	0.000104497	0.9916418
52	ENSMUST00000125250	Akna	0.000104806	0.99162945
53	chr3_86827945_86886695_R	Epha1	0.000105014	0.99162114
54	chr15_78403377_78412545_F	Snph	0.000105218	-0.991613
55	FR064590	Anxa13	0.000105229	0.99161253
56	FR186619	LOC101056101	0.000105419	-0.991605
57	FR011959	Gm16381	0.000105674	-0.9915948
58	chr8_123901815_123902167_R	Pitpnc1	0.000105739	0.99159222
59	FR092975	A930018P22Rik	0.000105824	-0.9915888
60	FR117744	Lrp2	0.000105886	0.99158637
61	Gomafu	Lao1	0.000105949	0.99158386
62	chr1_162965001_162966376_F	Slc39a4	0.000106006	-0.9915816
63	FR121241	LOC101056428	0.000106291	0.99157028
64	chr11_116936834_116948799_R	Anxa13	0.000106648	0.99155611
65	chrX_165792277_165807952_R	Dpp6	0.000106725	0.99155305
66	chr2_158169020_158191295_F	Prtn3	0.000106772	0.99155121
67	FR185165	LOC101056066	0.000106798	0.99155015
68	FR254927	Slc6a7	0.000106819	-0.9915493
69	FR155040	9130011E15Rik	0.000107011	0.99154172
70	chr4_116790373_116797823_F	LOC101056428	0.000107043	0.99154048
71	chr15_78403377_78412545_F	Mcts1	0.000107096	-0.9915384
72	FR011959	Pigr	0.000107222	-0.9915334
73	FR184042	6430571L13Rik	0.000107259	0.99153191
74	chr9_56646768_56649068_R	Ushbp1	0.000107385	-0.9915269
75	FR150621	Rundc3b	0.000107423	0.99152544
76	chr15_78403377_78412545_F	9130011E15Rik	0.000107424	-0.9915254
77	chr5_149751979_149808240_F	D830044I16Rik	0.000107453	0.99152427
78	chr4_35106125_35127700_F	9230106L01Rik	0.000107476	0.99152333
79	FR100940	Micu1	0.000107621	-0.9915176
80	chr4_116790373_116797823_F	Zfp866	0.000107661	0.99151605
81	n294011	9230106L01Rik	0.000107914	0.99150606
82	chr7_82902248_82955248_F	A930012N16Rik	0.00010807	-0.9914999
83	FR208702	Sycp2	0.000108176	0.99149573
84	FR055065	LOC101056428	0.000108212	0.99149432

85	chr8_26345571_26346095_R	Pdzk1	0.000108224	0.99149385
86	n273521	Nme4	0.000108427	0.99148586
87	chr14_55317199_55317670_R	1700084K02Rik	0.000108645	-0.9914773
88	FR375498	Kirrel3	0.000108673	0.9914762
89	ENSMUST00000155173	Tns4	0.000108797	0.99147135
90	FR112324	Socs6	0.000108865	0.99146868
91	FR114000	LOC101056101	0.000108945	-0.9914655
92	FR171433	Mettl7a2	0.000108973	0.99146445
93	FR294279	Gm10461	0.00010915	0.99145749
94	FR060087	Cd68	0.000109351	0.99144962
95	FR289473	Suv420h1	0.000109696	0.99143613
96	chr9_66976750_67014625_F	Aplnr	0.000109775	-0.991433
97	FR278883	Bcl3	0.000109817	0.99143138
98	FR001172	Mapk15	0.000109845	-0.9914303
99	n415399	Frzb	0.00010998	0.99142503
100	FR285140	Mettl7a2	0.000109985	-0.9914248
101	FR005836	Tns4	0.000110064	0.99142173
102	FR398266	Dpp10	0.000110283	0.9914132
103	chrX_165792277_165807952_R	Tnk1	0.0001103	-0.9914125
104	FR237950	LOC101056428	0.000110329	0.99141141
105	FR043548	Agbl2	0.000110359	-0.9914102
106	chr18_69923410_69965360_R	Gm16381	0.000110388	-0.9914091
107	n414371	Tns4	0.000110423	0.99140773
108	chr4_116790373_116797823_F	4933432I09Rik	0.000110501	0.9914047
109	n294791	Podxl	0.000110562	-0.9914023
110	n418283	Mcpt9	0.000110643	0.99139919
111	FR136228	Adam21	0.00011072	0.99139617
112	FR323263	Slc25a5	0.000111014	0.99138477
113	FR302003	C87490	0.000111087	0.99138191
114	FR337786	Ushbp1	0.000111264	-0.991375
115	FR331566	Tns4	0.00011148	0.99136665
116	FR139436	9330175E14Rik	0.000111485	0.99136648
117	chrX_102568332_102587093_R	Agbl2	0.000111577	0.99136289
118	n296070	Prtn3	0.000111595	-0.9913622
119	FR395572	9130011E15Rik	0.000111643	-0.9913603
120	FR312820	Gm581	0.000111739	-0.9913566
121	n343233	Slc25a1	0.000111967	-0.9913478
122	FR149067	Fancl	0.00011199	0.99134692
123	FR087735	Fancl	0.000112379	0.99133187
124	XR_140871.1	Nrxn2	0.000112489	-0.9913276
125	n296710	A030014E15Rik	0.000112538	0.99132575
126	n415372	4930548F15Rik	0.000112606	0.9913231
127	FR384764	1110008P14Rik	0.00011289	0.99131215

128	FR019091	Bnc2	0.000112944	0.99131007
129	FR064590	Gm6934	0.000112947	0.99130997
130	chr19_23068397_23190256_R	Elmsan1	0.000113098	-0.9913042
131	chr13_76326228_76332632_F	Prtn3	0.00011314	0.99130253
132	FR233225	Epha1	0.000113162	0.99130167
133	n418309	Gm581	0.00011319	-0.9913006
134	FR042390	Lrp2	0.000113437	0.9912911
135	n414371	LOC101056428	0.000113655	0.99128272
136	FR030947	Bnc2	0.000113783	0.9912778
137	chr1_196445304_196445818_R	Mylk2	0.000113918	0.99127262
138	FR199374	LOC101056101	0.000113962	-0.991271
139	chr2_158169020_158191295_F	Zfp866	0.000114076	0.99126657
140	FR194887	9030619P08Rik	0.000114156	0.99126349
141	n420390	Tmprss11bnl	0.000114341	0.99125644
142	n290305	Mcts1	0.000114498	0.99125039
143	chr12_16873410_16873872_F	Slc39a4	0.000114518	-0.9912497
144	chr3_86827945_86886695_R	Fam155a	0.000114544	0.99124864
145	FR087735	Ushbp1	0.000114606	0.99124627
146	ENSMUST00000157205	Slc10a5	0.000114742	0.99124107
147	chr12_111287772_111333890_F	Ushbp1	0.000114996	-0.9912314
148	FR237950	Tmprss11bnl	0.000115055	0.99122911
149	FR347914	Fut2	0.000115062	0.99122886
150	FR162622	Zfp689	0.000115146	0.99122564
151	FR029778	D830044I16Rik	0.000115242	0.99122199
152	chr10_8606683_8665783_R	Lipf	0.00011528	0.99122054
153	FR202182	LOC101056066	0.000115406	-0.9912157
154	chr1_183783306_183834531_F	Cpt2	0.000115464	-0.9912135
155	FR047247	Tns4	0.000115495	0.99121233
156	FR052210	Elmsan1	0.000115539	-0.9912107
157	FR366845	Sycp2	0.000115572	0.99120942
158	FR005836	Gm16381	0.000115621	-0.9912075
159	chr2_157236070_157249445_R	Aplnr	0.000115839	0.99119925
160	ENSMUST00000139056	Slc6a7	0.000115853	-0.9911987
161	Gomafu	LOC101056066	0.000115888	-0.9911974
162	uc.411+	Nme4	0.00011618	0.9911863
163	FR100940	Defb46	0.000116231	-0.9911843
164	n265955	Krtap1-5	0.000116448	0.99117609
165	FR029778	LOC101056428	0.00011648	0.99117488
166	FR202182	Snph	0.000116624	0.99116943
167	FR162485	LOC101056066	0.000116638	-0.9911689
168	chr15_96597330_96663683_F	Zfp473	0.000116679	-0.9911674
169	chr3_86827945_86886695_R	Sycp2	0.00011685	0.99116089
170	chr12_16873410_16873872_F	5033403F01Rik	0.000117158	-0.9911492

171	FR266999	Tescl	0.000117169	-0.9911488
172	FR121241	Gm16381	0.000117316	-0.9911432
173	FR019091	Fam155a	0.000117337	0.99114244
174	chr12_110003268_110030953_F	Lrp2	0.000117382	-0.9911408
175	Gomafu	9330133O14Rik	0.000117421	0.99113927
176	FR237950	Gm16381	0.000117458	-0.9911379
177	n416957	Fut2	0.000117474	0.99113726
178	ENSMUST00000181887	Prtn3	0.000117615	0.99113194
179	n418283	Akna	0.000117744	0.99112708
180	FR319563	Slc39a4	0.000117765	0.99112627
181	FR168142	Slc39a4	0.000117799	0.99112498
182	FR366845	Slc39a4	0.000117838	0.99112353
183	chr8_41475940_41476460_F	1700084K02Rik	0.000118227	0.99110887
184	chr9_14480441_14483362_F	Pifo	0.000118276	0.991107
185	FR185165	Pifo	0.000118279	0.99110691
186	FR283931	Nme4	0.000118352	-0.9911042
187	chr3_35782698_35789932_R	Bpifa1	0.000118462	0.99110003
188	FR162485	Zfp473	0.00011855	0.99109673
189	ENSMUST00000157205	Tns4	0.000118571	0.99109591
190	chr8_96983590_96994419_F	Rtp4	0.000118626	0.99109387
191	FR162622	Btnl1	0.000118637	0.99109344
192	chr7_91558475_91733625_R	Slc39a4	0.000118644	-0.9910932
193	FR171433	4930548F15Rik	0.000118655	0.99109278
194	chr8_26345571_26346095_R	D830044I16Rik	0.00011866	0.99109258
195	FR097477	Lao1	0.000118677	-0.9910919
196	FR199374	Gm4583	0.000118729	-0.99109
197	FR155040	Micu1	0.000118853	0.99108531
198	chr12_111287772_111333890_F	Mettl7a2	0.000118917	-0.9910829
199	FR331566	Gm16381	0.000118956	-0.9910814
200	FR136228	Zfp473	0.000118996	0.99107997
201	FR029778	A430061O12Rik	0.000119056	0.9910777
202	FR279905	Aqp4	0.000119121	0.99107528
203	FR287008	Tmprss11bnl	0.000119183	0.99107292
204	XR_104983.1	Snph	0.000119337	0.99106715
205	FR007551	1700029I15Rik	0.00011938	-0.9910656
206	uc.31+	A430060F13Rik	0.000119463	-0.9910625
207	chr1_183142656_183163206_R	Snph	0.000119501	-0.991061
208	FR186619	Micu1	0.000119613	0.99105682
209	FR301567	Lrp2	0.000119806	0.99104961
210	chr14_105497388_105511063_F	Tmod4	0.00011988	0.99104685
211	FR233225	9130011E15Rik	0.000120064	0.99103995
212	chr14_105497388_105511063_F	Anxa13	0.000120165	0.99103619
213	FR254927	Mcts1	0.000120254	0.99103284

214	FR155040	Fam155a	0.000120281	0.99103185
215	FR385633	Fut2	0.000120478	-0.9910245
216	FR032503	Suv420h1	0.00012048	0.99102443
217	FR185165	Mylk2	0.00012084	0.99101102
218	chr1_183142656_183163206_R	Bnc2	0.000120923	-0.9910079
219	chr11_116936834_116948799_R	Tmprss11bnl	0.000120958	0.99100661
220	FR087925	4930449A18Rik	0.000121046	-0.9910033
221	FR117744	Defb46	0.000121279	0.99099468
222	ENSMUST00000139056	Epha1	0.000121612	0.99098228
223	chr12_16873410_16873872_F	Suv420h1	0.000121701	-0.990979
224	FR319563	Gm10461	0.000121835	-0.990974
225	FR294279	Gm4583	0.00012185	0.99097344
226	chr5_31883451_31884082_F	Abcg3	0.000121853	0.99097336
227	chr6_52046805_52072744_R	Tmprss11bnl	0.000121892	0.9909719
228	NR_033641.2	Slc6a7	0.000121898	-0.9909717
229	FR298560	Fam155a	0.000122175	-0.9909614
230	FR212572	LOC101056428	0.000122438	0.99095168
231	chr2_151992806_152025456_F	Gm581	0.000122503	-0.9909493
232	chr12_89519675_89533975_F	C87490	0.000122574	0.99094664
233	FR283504	C87487	0.000122778	0.99093909
234	FR287008	Gm6934	0.000122825	0.99093735
235	chr14_105497388_105511063_F	Tmprss11bnl	0.000123025	0.99092999
236	chr1_75075500_75102200_R	A930018P22Rik	0.000123088	-0.9909276
237	n412060	9430076C15Rik	0.000123131	0.99092607
238	chr13_98281158_98285757_F	Zfp473	0.000123224	-0.9909226
239	n290544	Cnksr2	0.000123232	0.99092235
240	ENSMUST00000125250	Mylk2	0.000123425	0.99091522
241	chrX_102568332_102587093_R	Gm6934	0.000123434	0.99091488
242	FR202182	9130011E15Rik	0.000123628	0.99090775
243	ENSMUST00000180590	Cir1	0.000123639	0.99090734
244	FR279620	Fam155a	0.000123674	0.99090606
245	FR149067	Sycp2	0.000123684	0.99090569
246	chr1_138473514_138476054_R	Pifo	0.00012378	0.99090214
247	FR389545	Pih1d3	0.000124018	-0.9908934
248	chr15_60732209_60732822_R	Necab1	0.000124038	-0.9908926
249	chr9_14480441_14483362_F	Slc10a5	0.000124146	0.99088866
250	FR375498	Slc10a5	0.000124174	0.99088764
251	FR065159	LOC641235	0.000124247	-0.990885
252	FR029778	Zfp689	0.000124279	0.99088381
253	FR121241	Galp	0.000124347	0.99088131
254	XR_141342.2	Slc25a42	0.000124436	-0.990878
255	FR043548	Kcnq4	0.000124586	-0.9908725
256	FR032503	Abcg3	0.000124598	0.99087208

257	FR279620	Mettl7a2	0.000124642	0.99087046
258	chr8_41475940_41476460_F	Mboat4	0.000124642	0.99087049
259	FR005836	D830044I16Rik	0.000124706	0.99086811
260	ENSMUST00000155173	Tmprss11bnl	0.000124904	0.99086086
261	FR057053	Kirrel3	0.00012491	0.99086065
262	FR199374	A930018P22Rik	0.000124927	-0.99086
263	n296710	Akna	0.000124969	0.99085847
264	chr5_149751979_149808240_F	Gm16287	0.000125093	0.99085396
265	chr10_8606683_8665783_R	Prtn3	0.000125157	0.99085162
266	n418283	Tmprss11bnl	0.000125237	0.99084867
267	FR208702	Abcg3	0.000125601	0.99083538
268	FR065962	Zfp866	0.000125668	0.99083291
269	FR115704	Slc6a7	0.000125893	-0.9908247
270	FR254927	3110021A11Rik	0.000126062	-0.9908185
271	chr8_26345571_26346095_R	Prtn3	0.000126155	0.99081514
272	chr5_31883451_31884082_F	Tescl	0.000126245	0.99081185
273	n283487	Mcpt-ps1	0.000126273	-0.9908108
274	chr13_34693404_34717987_F	Necab1	0.00012628	0.99081057
275	chr14_20893694_20903726_F	Galp	0.000126352	0.99080795
276	n418309	Mcpt9	0.000126379	0.99080699
277	chr9_14480441_14483362_F	Kirrel3	0.000126457	0.99080415
278	n419646	Podxl	0.000126615	0.99079837
279	n265955	A930018P22Rik	0.000126782	0.9907923
280	FR065962	Gm6934	0.000126786	0.99079215
281	chr5_149751979_149808240_F	Prtn3	0.000126873	0.99078901
282	ENSMUST00000134129	Gatm	0.000127049	-0.9907826
283	FR162622	D830044I16Rik	0.000127345	0.99077187
284	NR_045899.1	Fam155a	0.000127347	0.99077177
285	chr14_25923295_25923893_F	Prtn3	0.000127433	0.99076866
286	chr13_98281158_98285757_F	Epha1	0.000127453	-0.9907679
287	FR184042	Mcoln3	0.000127596	-0.9907628
288	chr9_56646768_56649068_R	Pla2g10	0.0001276	-0.9907626
289	FR035623	Agbl2	0.000127605	0.99076244
290	FR185165	Slc39a4	0.000127649	-0.9907608
291	FR007873	Abcg3	0.000127669	0.99076012
292	chr2_158169020_158191295_F	Gm16381	0.00012767	-0.9907601
293	chr1_92546371_92551596_R	P2ry1	0.00012775	-0.9907572
294	chr12_16873410_16873872_F	Bnc2	0.000127783	-0.990756
295	chr9_67414463_67483185_R	Galp	0.000127868	0.99075291
296	chr7_26212709_26230775_F	Lax1	0.00012806	0.99074595
297	FR168142	Lrp2	0.000128225	0.99073997
298	FR074898	Defb46	0.000128262	0.99073862
299	Gomafu	4930548F15Rik	0.000128294	0.99073748

300	FR019091	Mettl7a2	0.000128296	0.99073741
301	FR152607	Pigr	0.000128403	-0.9907335
302	FR237950	Kcnq4	0.000128447	0.99073194
303	FR238565	Fut2	0.000128532	0.99072887
304	chr10_18036490_18059965_F	Cox15	0.000128699	0.99072285
305	FR238565	Suv420h1	0.000128711	0.9907224
306	FR152607	Prtn3	0.000128747	0.99072113
307	FR238898	A730021G18Rik	0.000129037	0.99071065
308	FR326589	9130011E15Rik	0.000129315	0.99070063
309	chr1_183142656_183163206_R	Mettl7a2	0.000129318	-0.9907005
310	chr15_100908323_100908645_F	Gm581	0.000129375	-0.9906985
311	FR219193	Olfr319	0.000129382	0.99069824
312	n290305	5033403F01Rik	0.000129677	0.99068762
313	n290305	Ushbp1	0.000129738	0.99068541
314	chr8_41475940_41476460_F	Slc17a9	0.000129791	0.9906835
315	uc.411+	2310067P03Rik	0.000129973	0.99067699
316	chr12_55271691_55276492_R	Zfp473	0.000130033	0.99067481
317	chr2_72818943_72826893_R	Prtn3	0.000130424	0.99066078
318	FR202182	Gm3952	0.000130444	0.99066009
319	FR283504	Mcoln3	0.000130481	-0.9906587
320	FR247760	Cd180	0.00013061	0.99065411
321	FR066795	Slc6a7	0.000130693	0.99065116
322	FR283931	Fam155a	0.000130702	-0.9906508
323	FR155040	Snph	0.000130789	0.99064771
324	NR_033641.2	Fancl	0.000131019	0.99063949
325	chr6_47959825_47960502_R	Ighv1-77	0.000131153	-0.9906347
326	n290305	Epha1	0.000131308	0.99062916
327	FR168142	Fancl	0.000131356	0.99062742
328	chr6_52046805_52072744_R	Tmod4	0.000131768	0.99061273
329	FR092975	Pifo	0.000131898	-0.9906081
330	FR097477	Gm4583	0.000132057	0.99060242
331	chr14_105497388_105511063_F	Gm6934	0.000132149	0.99059914
332	chr1_196445304_196445818_R	2310067P03Rik	0.000132419	-0.9905895
333	FR285140	Slc17a9	0.000132445	-0.9905886
334	FR114000	Adam21	0.000132482	0.99058728
335	FR055065	Gm6934	0.000132525	0.99058575
336	chr1_75075500_75102200_R	Bnc2	0.000132585	0.9905836
337	n272641	Gsdma2	0.000132606	0.99058287
338	chr2_151992806_152025456_F	Kcnq4	0.000132998	0.99056894
339	n416957	Icos	0.000133083	0.9905659
340	FR117744	Fam155a	0.000133124	0.99056447
341	chr1_162965001_162966376_F	Eps15l1	0.000133143	0.9905638
342	FR171433	Abcg3	0.000133281	0.99055891

343	NR_076393.1	Slc34a3	0.000133313	-0.9905578
344	ENSMUST00000130677	Cd300ld	0.000133577	-0.9905484
345	FR090859	Slc6a7	0.000133661	-0.9905454
346	chr7_80762708_80763274_R	Rsph3a	0.000133786	-0.990541
347	chr1_167515313_167516122_F	Btnl1	0.000133801	0.99054046
348	FR087537	S100b	0.000133883	-0.9905376
349	chr8_26345571_26346095_R	Mylk2	0.000133909	0.99053665
350	FR097477	Sycp2	0.00013393	-0.9905359
351	chr8_119732348_119749198_R	Ikzf4	0.000134216	0.99052579
352	FR162622	Kcnq4	0.000134247	0.99052469
353	FR032503	Bnc2	0.000134322	0.99052202
354	ENSMUST00000141575	Zfp866	0.000134515	0.99051521
355	chr15_38205154_38206196_F	Podxl	0.000134751	-0.9905069
356	chr3_40439525_40509975_R	Gm16381	0.000135283	-0.9904881
357	chr13_48576557_48581632_F	Anxa13	0.000135407	0.99048377
358	FR326589	Defb46	0.000135422	0.99048323
359	FR187810	Mical2	0.000135465	0.99048173
360	FR233225	Suv420h1	0.000135475	0.99048139
361	FR302411	C87487	0.000135527	-0.9904796
362	chr1_138570107_138632310_R	A930018P22Rik	0.000135584	0.99047755
363	chr10_8606683_8665783_R	Sdk2	0.000135607	0.99047673
364	FR140001	Socs6	0.000135638	0.99047566
365	FR054476	Zfp689	0.000135655	0.99047505
366	n294791	Epha1	0.000135656	0.99047502
367	FR162622	Tmod4	0.000135896	0.99046657
368	n414371	Gm10461	0.000136336	0.99045113
369	ENSMUST00000155173	Gm581	0.000136417	-0.9904483
370	FR326589	Epha1	0.000136484	0.99044595
371	FR028809	9330175E14Rik	0.000136486	0.99044586
372	n290305	Mboat4	0.000136663	0.99043968
373	FR202182	Gm4583	0.000136677	-0.9904392
374	FR215734	Grid2	0.000136787	-0.9904353
375	ENSMUST00000155173	Akna	0.000136962	0.99042921
376	FR233225	Fam155a	0.000137069	0.99042544
377	chr9_4260788_4261404_F	3110021A11Rik	0.000137242	0.9904194
378	NR_033641.2	Snph	0.000137396	0.99041404
379	n287284	Npy	0.000137407	-0.9904136
380	FR202182	Fancl	0.000137711	0.99040302
381	chr13_98513868_98517366_R	Eps15l1	0.000137805	0.99039973
382	FR007873	Brpf1	0.000137872	-0.9903974
383	FR155040	Zfp473	0.000137991	0.99039326
384	FR121323	Prtn3	0.000138172	0.99038695
385	chr9_67414463_67483185_R	Krtap1-5	0.000138226	0.99038505

386	FR168142	Mettl7a2	0.000138374	0.99037992
387	n418283	Galp	0.000138471	0.99037652
388	n296710	C87490	0.000138587	0.99037249
389	chr8_26345571_26346095_R	Tmprss11bnl	0.000138601	0.99037201
390	XR_141927.1	Kcnq4	0.000138641	0.99037062
391	chr4_88745771_88776323_F	Agbl2	0.000138726	0.99036768
392	FR019091	4930548F15Rik	0.000138738	0.99036723
393	n418283	Tns4	0.000138887	0.99036207
394	chr1_183142656_183163206_R	Abcg3	0.000139137	-0.9903534
395	FR121323	Gm16381	0.000139153	-0.9903528
396	FR389545	Tas2r125	0.000139557	0.99033879
397	n290305	Fam155a	0.000139882	0.99032754
398	FR375498	Cd200r4	0.000139963	-0.9903248
399	FR168142	Gm4583	0.000139964	-0.9903247
400	ENSMUST00000163396	Slc17a9	0.00014012	-0.9903193
401	chr12_110003268_110030953_F	A930018P22Rik	0.000140301	0.99031307
402	FR043548	Tmod4	0.000140359	-0.990311
403	chr9_58301467_58323783_F	Lao1	0.000140398	-0.9903097
404	chr7_80762708_80763274_R	Pifo	0.00014059	0.99030308
405	FR279620	Slc6a7	0.000140633	-0.9903016
406	FR388349	Ces1f	0.000140761	0.99029716
407	FR215734	Icos	0.000140903	-0.9902922
408	FR055065	Hoxa9	0.000141172	0.99028298
409	FR087516	Aplnr	0.000141289	-0.990279
410	FR074898	Micu1	0.000141389	0.9902755
411	FR326589	Micu1	0.000141674	0.99026568
412	FR057053	Tsc22d4	0.000141918	0.99025731
413	FR035623	Kcnq4	0.000141952	0.99025611
414	chr5_31883451_31884082_F	Lrp2	0.000142216	0.99024704
415	n289317	2310067P03Rik	0.00014233	0.99024313
416	FR064590	Tmprss11bnl	0.000142422	0.99023997
417	FR100940	Suv420h1	0.000142481	-0.990238
418	chr3_86827945_86886695_R	Bnc2	0.000142504	0.99023717
419	FR351980	Cd180	0.000142736	0.99022921
420	FR090859	Slc39a4	0.00014284	0.99022566
421	ENSMUST00000158863	Ikzf4	0.000142857	-0.9902251
422	FR064590	Ptchd4	0.000143008	0.9902199
423	chr10_8606683_8665783_R	A430061O12Rik	0.000143134	0.99021557
424	n289590	Chchd10	0.000143228	0.99021234
425	chr8_41475940_41476460_F	9330133O14Rik	0.000143482	0.99020366
426	chr15_78403377_78412545_F	Bnc2	0.000144295	-0.9901759
427	FR374009	Bnc2	0.000144391	0.99017265
428	FR140001	4930548F15Rik	0.000144857	0.99015678

429	ENSMUST00000158863	Mylk2	0.000145252	-0.9901433
430	XR_141927.1	Sdk2	0.000145626	0.99013062
431	FR005836	Btnl1	0.000145855	0.99012286
432	FR374009	Elmsan1	0.000145915	0.99012081
433	FR044110	Selp	0.00014598	0.99011864
434	chr18_5813291_5813708_R	LOC101056428	0.000146067	-0.9901157
435	FR266999	Gm10461	0.00014666	0.99009561
436	FR387804	9330133O14Rik	0.00014699	0.99008444
437	FR202182	Fam155a	0.000147007	0.99008388
438	FR312820	Cd200r4	0.000147025	-0.9900833
439	chr15_60732209_60732822_R	9430076C15Rik	0.000147145	0.99007921
440	FR185165	Slc17a9	0.000147197	-0.9900774
441	FR238565	Slc6a7	0.000147352	-0.9900722
442	chr9_58301467_58323783_F	Pih1d3	0.00014765	0.99006216
443	chr12_110003268_110030953_F	LOC101056066	0.000147735	0.99005931
444	FR279620	9130011E15Rik	0.000147846	0.99005555
445	NR_045776.1	9330133O14Rik	0.000148033	0.99004926
446	FR237950	D830044I16Rik	0.00014819	0.99004398
447	FR279620	2310067P03Rik	0.000148219	0.99004301
448	FR112324	Elmsan1	0.000148272	0.99004121
449	XR_105914.2	Prss55	0.00014842	0.99003624
450	FR254927	Mboat4	0.0001485	0.99003354
451	FR301809	Adam21	0.000148587	0.99003062
452	FR347914	Micu1	0.000148643	0.99002876
453	FR302003	Abcg3	0.000148911	-0.9900197
454	n273521	A430060F13Rik	0.000149111	0.99001303
455	FR202182	Elmsan1	0.000149277	0.99000746
456	chr14_55317199_55317670_R	Sycp2	0.000149362	-0.9900046
457	FR155040	Gm3952	0.000149384	0.9900039
458	n289317	LOC101056066	0.000149402	-0.9900033
459	FR331566	Mylk2	0.000149465	0.99000119
460	FR285140	LOC101056066	0.000149576	0.98999745
461	n343177	Socs6	0.000149587	0.98999709
462	FR030947	Mboat4	0.000149593	0.98999688
463	FR168142	Abcg3	0.00014963	0.98999563
464	FR266999	Fancl	0.000149643	-0.9899952
465	chr12_89519675_89533975_F	Gm16381	0.000149679	-0.989994
466	FR171433	5033403F01Rik	0.000149725	0.98999245
467	FR149067	Ushbp1	0.000149913	0.98998617
468	FR302411	Slc25a5	0.000150041	0.9899819
469	chr6_129151512_129208737_R	A030001D20Rik	0.000150066	-0.9899811
470	chr17_64063362_64066067_R	Prss55	0.000150346	-0.9899717
471	FR238565	Adam21	0.000150497	0.98996667

472	XR_141342.2	Prkar2b	0.000150778	-0.9899573
473	chr1_75075500_75102200_R	Adam21	0.000150841	0.98995516
474	chr13_98513868_98517366_R	Zfp866	0.000150898	0.98995326
475	chr15_96597330_96663683_F	Nme4	0.000150949	-0.9899516
476	chr2_71576218_71603818_R	9430076C15Rik	0.000150958	0.98995127
477	ENSMUST00000139056	Snph	0.000150982	0.98995046
478	FR092975	Gm4955	0.000151007	0.98994964
479	chr1_196445304_196445818_R	Abcg3	0.000151026	-0.989949
480	n289317	Lrp2	0.000151145	0.98994503
481	n280963	Il1rn	0.000151214	0.98994273
482	NR_045776.1	Cnksr2	0.000151493	0.98993347
483	FR233225	LOC101056101	0.000151522	-0.9899325
484	FR294279	Fancl	0.00015164	-0.9899286
485	FR238565	Lrp2	0.000151719	0.98992593
486	FR385633	Zfp473	0.000151769	-0.9899243
487	FR035623	Tns4	0.000151774	0.98992409
488	chr15_38205154_38206196_F	Lao1	0.000151839	0.98992193
489	FR057053	Mylk2	0.000151887	0.98992034
490	chr1_138473514_138476054_R	Krtap1-5	0.000152037	0.98991536
491	FR199374	Gm10461	0.000152228	-0.989909
492	ENSMUST00000157205	Gm581	0.000152262	-0.9899079
493	chr13_98038568_98068079_F	Sycp2	0.000152326	0.98990578
494	chr19_23068397_23190256_R	1700084K02Rik	0.00015244	-0.989902
495	chr17_29374758_29387733_R	LOC101056066	0.000152475	0.98990083
496	FR238565	9330175E14Rik	0.000152595	-0.9898968
497	chr1_75075500_75102200_R	Snph	0.000152653	0.98989494
498	chr15_38205154_38206196_F	Pih1d3	0.000152664	-0.9898946
499	chr8_26345571_26346095_R	Dnmt3l	0.000152799	0.9898901
500	FR057053	Pifo	0.000153011	0.98988306

Table-S4 GO functional enrichment analysis of top 500 lncRNA-correlated mRNAs

No	groups	enrichmentTerm	p.value	enrichment IDs	GO_term	Category
1	chr1_134167181_134181386_R	chromosome	0.000215058	Ctc1; Rad21l; Spata22; Suv420h1; Sycp2; Xist	chromosome	Component
2	chr1_134167181_134181386_R	transcytosis	0.000257462	Rab17; Tg	transcytosis	Process
3	chr1_134167181_134181386_R	fertilization	0.000265772	Rad21l; Spata22; Sycp2	fertilization	Process
4	chr1_134167181_134181386_R	chaperone binding	0.000501509	Hspe1; Pih1d3; Tg	chaperone binding	Function
5	chr1_138570107_138632310_R	brush border membrane	0.000430827	Folr1; Lrp2; Pdzk1; Shank2	brush border membrane	Component
6	chr1_162965001_162966376_F	brush border membrane	0.000409642	Folr1; Lrp2; Pdzk1; Shank2	brush border membrane	Component
7	chr1_167515313_167516122_F	brush border membrane	0.000350557	Folr1; Pdzk1; Shank2; Slc34a3	brush border membrane	Component
8	chr1_183783306_183834531_F	transport	0.000154079	Abcb8; Abcg3; Aqp4; Atp5h; Chmp4c; Cpt2; Etfdh; Fabp3; Folr1; Grid2; Grik3; Kcnk4; Kcnq4; Mcoln3; Micu1; Mup20; Pdzk1; Pex14; Pitpnc1; Rab17; Slc10a5; Slc17a9; Slc25a1; Slc25a42; Slc25a5; Slc39a4; Slc6a7; Trpm3	transport	Process
9	chr1_196445304_196445818_R	brush border membrane	0.000420139	Folr1; Lrp2; Pdzk1; Shank2	brush border membrane	Component
10	chr1_69531191_69570566_F	phospholipase activity	0.000338157	Abhd15; Nceh1; Pla2g10	phospholipase activity	Function
11	chr1_75075500_75102200_R	PDZ domain binding	0.000465558	Grid2; Kirrel3; Lrp2; Pdzk1; Ushbp1	PDZ domain binding	Function
12	chr1_92546371_92551596_R	transport	0.00024062	Abcb8; Chmp4c; Cpt2; Etfdh; Fabp3; Grid2; Kcnk4; Mcoln3; Micu1; Mup20; Pitpnc1; Rab17; Slc25a1; Slc25a42; Slc25a5; Slc39a4; Slc6a7; Trpm3	transport	Process
13	chr1_92549499_92550225_R	transport	0.000229024	Abcb8; Aqp4; Atp5h; Chmp4c; Cpt2; Etfdh; Fabp3; Grid2; Kcnk4; Kcnq4; Mcoln3; Mup20; Pdzk1; Pex14; Pitpnc1; Rab17; Slc17a9; Slc25a42; Slc25a5; Slc39a4; Slc6a7; Trpm3	transport	Process
14	chr1_92549499_92550225_R	transporter activity	0.000407316	Aqp4; Fabp3; Grid2; Mup20; Slc17a9; Slc25a5	transporter activity	Function

15	chr1_92549499_92550225_R	fatty acid beta-oxidation using acyl-CoA dehydrogenase	0.000418462	Acad11; Etfdh	fatty acid beta-oxidation using acyl-CoA dehydrogenase	Process
16	chr10_116814196_116920696_R	regulation of body fluid levels	0.000371315	Apln; Aplnr	regulation of body fluid levels	Process
17	chr10_39666719_39679959_R	transporter activity	0.000111808	Aqp4; Fabp3; Grid2; Mup20; Slc17a9; Slc22a23; Slc25a5	transporter activity	Function
18	chr10_39666719_39679959_R	receptor binding	0.000244324	Apln; Galp; Gdf5; Kng2; Pex14; S100b; Six3; Tg; Wnt8a	receptor binding	Function
19	chr10_8606683_8665783_R	brush border membrane	0.000399334	Folr1; Lrp2; Pdzk1; Shank2	brush border membrane	Component
20	chr11_116936834_116948799_R	brush border membrane	0.000273743	Lrp2; Pdzk1; Shank2; Slc34a3	brush border membrane	Component
21	chr11_120041124_120052299_F	receptor activity	0.000403666	Cd200r4; Folr1; Grid2; Grik3; Lrp2; Plxna4	receptor activity	Function
22	chr11_120079199_120130099_R	mitochondrial inner membrane	0.000185336	Abcb8; Atp5h; Cox15; Cpt2; Etfdh; Gatm; Idh2; Micu1; Slc25a1; Slc25a5	mitochondrial inner membrane	Component
23	chr11_120079199_120130099_R	apical plasma membrane	0.000337522	Gp2; Lrp2; P2ry1; Pdzk1; Podxl; Rab17; Shank2; Slc39a4	apical plasma membrane	Component
24	chr11_120079199_120130099_R	transport	0.000369785	Abcb8; Abcg3; Apoc1; Aqp4; Atp5h; Chmp4c; Cpt2; Etfdh; Fabp3; Folr1; Grid2; Grik3; Kcnk4; Kcnq4; Mcoln3; Micu1; Pdzk1; Pitpnc1; Rab17; Slc10a5; Slc17a9; Slc25a1; Slc25a5; Slc39a4; Slc6a7; Trpm3	transport	Process
25	chr11_33414450_33450750_F	transcytosis	0.00034282	Rab17; Tg	transcytosis	Process
26	chr11_33414450_33450750_F	fertilization	0.000405384	Rad21l; Spata22; Sycp2	fertilization	Process
27	chr11_6796679_6797670_F	brush border membrane	0.000350557	Folr1; Lrp2; Pdzk1; Shank2	brush border membrane	Component
28	chr11_69184157_69201732_R	brush border membrane	0.000236738	Lrp2; Pdzk1; Shank2; Slc34a3	brush border membrane	Component
29	chr11_87841800_87858725_F	apical plasma membrane	0.000199219	Gp2; Lrp2; P2ry1; Pdzk1; Podxl; Rab17; Slc39a4	apical plasma membrane	Component
30	chr11_87841800_87858725_F	PDZ domain binding	0.000286675	Grid2; Kirrel3; Lrp2; Pdzk1; Ushbp1	PDZ domain binding	Function
31	chr11_87841800_87858725_F	mitochondrial inner membrane	0.000320715	Abcb8; Cox15; Cpt2; Gatm; Idh2; Micu1; Slc25a1; Slc25a5	mitochondrial inner membrane	Component
32	chr12_110003268_110030953_F	mitochondrial inner membrane	0.000364271	Abcb8; Atp5h; Cox15; Cpt2; Gatm; Idh2; Micu1; Slc25a1; Slc25a5	mitochondrial inner membrane	Component
33	chr12_110003268_110030953_F	brush border membrane	0.000420139	Folr1; Lrp2; Pdzk1; Shank2	brush border membrane	Component

34	chr12_111287772_111333890_F	mitochondrial inner membrane	0.000364271	Abcb8; Atp5h; Cox15; Cpt2; Gatm; Idh2; Micu1; Slc25a1; Slc25a5	mitochondrial inner membrane	Component
35	chr12_55271691_55276492_R	apical plasma membrane	0.000299072	Gp2; Lrp2; P2ry1; Pdzk1; Podxl; Rab17; Slc39a4	apical plasma membrane	Component
36	chr12_55271691_55276492_R	PDZ domain binding	0.000388956	Grid2; Kirrel3; Lrp2; Pdzk1; Ushbp1	PDZ domain binding	Function
37	chr12_55271691_55276492_R	receptor activity	0.000439935	Cd200r4; Folr1; Grid2; Grik3; Lrp2; Plxna4	receptor activity	Function
38	chr12_89519675_89533975_F	brush border membrane	0.000409642	Folr1; Lrp2; Pdzk1; Shank2	brush border membrane	Component
39	chr13_107822042_107823753_F	apical plasma membrane	0.000345526	Gp2; Lrp2; P2ry1; Pdzk1; Podxl; Rab17; Slc39a4	apical plasma membrane	Component
40	chr13_107822042_107823753_F	PDZ domain binding	0.000433668	Grid2; Kirrel3; Lrp2; Pdzk1; Ushbp1	PDZ domain binding	Function
41	chr13_107822042_107823753_F	receptor activity	0.000499048	Cd200r4; Folr1; Grid2; Grik3; Lrp2; Plxna4	receptor activity	Function
42	chr13_15947521_15999246_R	mitochondrial inner membrane	0.000194494	Abcb8; Atp5h; Cox15; Cpt2; Etfdh; Gatm; Idh2; Micu1; Slc25a1; Slc25a5	mitochondrial inner membrane	Component
43	chr13_34872696_34906407_R	regulation of body fluid levels	0.000371315	Apln; Aplnr	regulation of body fluid levels	Process
44	chr13_66381200_66410200_F	brush border membrane	0.000369523	Lrp2; Pdzk1; Shank2; Slc34a3	brush border membrane	Component
45	chr13_74642700_74701900_F	PDZ domain binding	0.000286675	Grid2; Kirrel3; Lrp2; Pdzk1; Ushbp1	PDZ domain binding	Function
46	chr13_74642700_74701900_F	receptor activity	0.000308692	Cd200r4; Folr1; Grid2; Grik3; Lrp2; Plxna4	receptor activity	Function
47	chr13_76326228_76332632_F	transport	0.000303198	Abcb8; Abcg3; Apoc1; Aqp4; Atp5h; Chmp4c; Cpt2; Etfdh; Fabp3; Folr1; Grid2; Grik3; Kcnk4; Kcnq4; Mcoln3; Micu1; Pdzk1; Pex14; Pitpnc1; Rab17; Slc10a5; Slc17a9; Slc22a23; Slc25a5; Slc39a4; Slc6a7; Trpm3	transport	Process
48	chr13_98281158_98285757_F	apical plasma membrane	0.000498152	Gp2; Lrp2; P2ry1; Pdzk1; Podxl; Rab17; Slc39a4	apical plasma membrane	Component
49	chr14_105497388_105511063_F	brush border membrane	0.000266031	Lrp2; Pdzk1; Shank2; Slc34a3	brush border membrane	Component
50	chr14_14688472_14747272_F	regulation of body fluid levels	0.000431674	Apln; Aplnr	regulation of body fluid levels	Process
51	chr14_21621875_21638625_R	PDZ domain binding	0.000415591	Frzb; Grid2; Kirrel3; Lrp2; Pdzk1; Ushbp1	PDZ domain binding	Function
52	chr14_25923295_25923893_F	PDZ domain binding	0.000351966	Frzb; Grid2; Kirrel3; Lrp2; Pdzk1; Ushbp1	PDZ domain binding	Function

53	chr14_25923295_25923893_F	transport	0.000466309	Abcb8; Abcg3; Apoc1; Aqp4; Atp5h; Chmp4c; Cpt2; Etfdh; Folr1; Grid2; Grik3; Kcnk4; Kcnq4; Mcoln3; Micu1; Pdzk1; Pex14; Pitpnc1; Rab17; Slc10a5; Slc17a9; Slc22a23; Slc25a1; Slc25a5; Slc34a3; Slc39a4; Slc6a7; Trpm3	transport	Process
54	chr14_25923295_25923893_F	mitochondrial inner membrane	0.000474944	Abcb8; Atp5h; Cox15; Cpt2; Etfdh; Gatm; Idh2; Micu1; Slc25a1; Slc25a5	mitochondrial inner membrane	Component
55	chr14_73729977_73751877_R	regulation of body fluid levels	0.000447463	Apln; Aplnr	regulation of body fluid levels	Process
56	chr14_77511189_77522852_F	negative regulation of Wnt signaling pathway	0.000147037	Dkk2; Frzb; Six3	negative regulation of Wnt signaling pathway	Process
57	chr15_100908323_100908645_F	apical plasma membrane	0.000298978	Gp2; Lrp2; Pdzk1; Podxl; Rab17; Shank2; Slc34a3; Slc39a4	apical plasma membrane	Component
58	chr15_32167516_32174417_R	apical plasma membrane	0.000362234	Gp2; Lrp2; P2ry1; Pdzk1; Podxl; Rab17; Slc39a4	apical plasma membrane	Component
59	chr15_32167516_32174417_R	PDZ domain binding	0.0004494	Grid2; Kirrel3; Lrp2; Pdzk1; Ushbp1	PDZ domain binding	Function
60	chr15_78403377_78412545_F	apical plasma membrane	0.000245075	Gp2; Lrp2; P2ry1; Pdzk1; Podxl; Rab17; Slc39a4	apical plasma membrane	Component
61	chr15_78403377_78412545_F	PDZ domain binding	0.000334867	Grid2; Kirrel3; Lrp2; Pdzk1; Ushbp1	PDZ domain binding	Function
62	chr15_78403377_78412545_F	receptor activity	0.000369777	Cd200r4; Folr1; Grid2; Grik3; Lrp2; Plxna4	receptor activity	Function
63	chr16_44720170_44723911_R	regulation of body fluid levels	0.000264062	Apln; Aplnr	regulation of body fluid levels	Process
64	chr16_4871449_4874336_R	PDZ domain binding	0.000361146	Grid2; Kirrel3; Lrp2; Pdzk1; Ushbp1	PDZ domain binding	Function
65	chr17_29374758_29387733_R	brush border membrane	0.000314733	Folr1; Lrp2; Pdzk1; Shank2	brush border membrane	Component
66	chr18_5813291_5813708_R	brush border membrane	0.000499073	Folr1; Lrp2; Pdzk1; Shank2	brush border membrane	Component
67	chr18_67435075_67447150_F	brush border membrane	0.000420139	Folr1; Lrp2; Pdzk1; Shank2	brush border membrane	Component
68	chr18_69923410_69965360_R	transport	0.000305466	Abcb8; Abcg3; Apoc1; Aqp4; Atp5h; Chmp4c; Cpt2; Etfdh; Folr1; Grid2; Grik3; Kcnk4; Kcnq4; Mcoln3; Micu1; Pdzk1; Pex14; Rab17; Slc10a5; Slc17a9; Slc22a23; Slc25a5; Slc34a3; Slc39a4; Slc6a7; Trpm3	transport	Process
69	chr18_69923410_69965360_R	apical plasma membrane	0.000311404	Gp2; Lrp2; Pdzk1; Podxl; Rab17; Shank2; Slc34a3; Slc39a4	apical plasma membrane	Component
70	chr18_70400082_70404236_R	receptor binding	0.000115976	Apln; Gdf5; Kng2; Pex14; S100b; Six3; Tg; Wnt8a	receptor binding	Function

71	chr18_70400082_70404236_R	regulation of body fluid levels	0.000378615	Apln; Aplnr	regulation of body fluid levels	Process
72	chr19_23068397_23190256_R	brush border membrane	0.000379277	Folr1; Lrp2; Pdzk1; Shank2	brush border membrane	Component
73	chr19_33049479_33054553_R	extracellular space	0.000124513	Angptl4; Apln; Cx3cl1; Dkk2; Frzb; Kng2; Lgals3bp; Npy; Pla2g7; S100b; Selp; Spp1; Vcam1	extracellular space	Component
74	chr19_33049479_33054553_R	amine metabolic process	0.000180356	Cyp1a1; Vcam1	amine metabolic process	Process
75	chr19_33049479_33054553_R	IPAF inflammasome complex	0.000180356	Casp4; Naip2	IPAF inflammasome complex	Component
76	chr19_33049479_33054553_R	regulation of body fluid levels	0.000251922	Apln; Aplnr	regulation of body fluid levels	Process
77	chr19_33049479_33054553_R	pyroptosis	0.000335131	Casp4; Naip2	pyroptosis	Process
78	chr19_33049479_33054553_R	cell adhesion	0.000426019	Cx3cl1; Lgals3bp; Nrp2; Sdc3; Sdk2; Selp; Spp1; Vcam1	cell adhesion	Process
79	chr19_33049479_33054553_R	negative regulation of vasoconstriction	0.000429902	Apln; Cx3cl1	negative regulation of vasoconstriction	Process
80	chr19_33049479_33054553_R	negative regulation of interleukin-1 beta production	0.000429902	Acp5; Aqp4	negative regulation of interleukin-1 beta production	Process
81	chr19_33049479_33054553_R	blood microparticle	0.000459199	Angptl4; Cpn2; Kng2; Lgals3bp	blood microparticle	Component
82	chr2_108456668_108532418_R	apical plasma membrane	0.000329441	Gp2; Lrp2; P2ry1; Pdzk1; Podxl; Rab17; Slc39a4	apical plasma membrane	Component
83	chr2_108456668_108532418_R	PDZ domain binding	0.000418354	Grid2; Kirrel3; Lrp2; Pdzk1; Ushbp1	PDZ domain binding	Function
84	chr2_108456668_108532418_R	receptor activity	0.000478696	Cd200r4; Folr1; Grid2; Grik3; Lrp2; Plxna4	receptor activity	Function
85	chr2_11454222_11456321_R	apical plasma membrane	0.000477409	Gp2; Lrp2; P2ry1; Pdzk1; Rab17; Slc39a4	apical plasma membrane	Component
86	chr2_132722530_132726802_F	regulation of body fluid levels	0.000423885	Apln; Aplnr	regulation of body fluid levels	Process
87	chr2_157236070_157249445_R	PDZ domain binding	0.000199205	Frzb; Grid2; Kirrel3; Lrp2; Pdzk1; Ushbp1	PDZ domain binding	Function
88	chr2_157236070_157249445_R	apical plasma membrane	0.0003654	Gp2; Lrp2; Pdzk1; Podxl; Rab17; Shank2; Slc34a3; Slc39a4	apical plasma membrane	Component
89	chr2_157838320_157853495_F	brush border membrane	0.000306205	Folr1; Lrp2; Pdzk1; Shank2	brush border membrane	Component
90	chr2_158169020_158191295_F	transport	0.000409825	Abcb8; Abcg3; Apoc1; Aqp4; Atp5h; Chmp4c; Cpt2; Etfdh; Folr1; Grid2; Grik3; Kcnk4; Mcoln3; Micu1; Pex14; Pitpnc1; Rab17; Slc10a5; Slc17a9; Slc22a23; Slc25a5; Slc34a3; Slc39a4; Slc6a7; Trpm3	transport	Process
91	chr2_71576218_71603818_R	brush border membrane	0.000108964	Folr1; Lrp2; Pdzk1; Shank2; Slc34a3	brush border membrane	Component

92	chr2_71576218_71603818_R	PDZ domain binding	0.00050081	Frzb; Grid2; Kirrel3; Lrp2; Pdzk1; Ushbp1	PDZ domain binding	Function
93	chr2_72818943_72826893_R	apical plasma membrane	0.000253252	Gp2; Lrp2; Pdzk1; Podxl; Rab17; Shank2; Slc34a3; Slc39a4	apical plasma membrane	Component
94	chr3_21964899_21974524_F	fatty acid beta-oxidation using acyl-CoA dehydrogenase	0.000189057	Acad11; Etfdh	fatty acid beta-oxidation using acyl-CoA dehydrogenase	Process
95	chr3_21964899_21974524_F	receptor binding	0.000231604	Apln; Gdf5; Kng2; Pex14; S100b; Six3; Wnt8a	receptor binding	Function
96	chr3_21964899_21974885_F	mitochondrial inner membrane	0.000230201	Abcb8; Atp5h; Cox15; Cpt2; Etfdh; Gatm; Idh2; Micu1; Slc25a5	mitochondrial inner membrane	Component
97	chr3_21964899_21974885_F	transport	0.00036677	Abcb8; Abcg3; Atp5h; Chmp4c; Cpt2; Etfdh; Fabp3; Folr1; Grid2; Grik3; Kcnk4; Mcoln3; Micu1; Mup20; Pdzk1; Pex14; Pitpnc1; Rab17; Slc17a9; Slc25a5; Slc39a4; Slc6a7; Trpm3	transport	Process
98	chr3_35782698_35789932_R	transport	0.000113097	Abcb8; Abcg3; Atp5h; Chmp4c; Cpt2; Etfdh; Fabp3; Folr1; Grid2; Kcnk4; Kcnq4; Mcoln3; Micu1; Mup20; Pdzk1; Pex14; Pitpnc1; Rab17; Slc10a5; Slc17a9; Slc25a42; Slc39a4; Slc6a7; Trpm3	transport	Process
99	chr3_35782698_35789932_R	mitochondrial inner membrane	0.000206825	Abcb8; Atp5h; Cox15; Cpt2; Etfdh; Gatm; Idh2; Micu1; Slc25a42	mitochondrial inner membrane	Component
100	chr3_40439525_40509975_R	brush border membrane	0.000475526	Folr1; Lrp2; Pdzk1; Shank2	brush border membrane	Component
101	chr4_123572090_123581365_F	pyroptosis	0.000133164	Casp4; Naip2	pyroptosis	Process
102	chr4_123572090_123581365_F	extracellular space	0.000138914	Angptl4; Apln; Cx3cl1; Dkk2; Frzb; Gdf5; Lrg1; Selp; Timp1; Vcam1	extracellular space	Component
103	chr4_123572090_123581365_F	negative regulation of vasoconstriction	0.000170968	Apln; Cx3cl1	negative regulation of vasoconstriction	Process
104	chr4_123572090_123581365_F	negative regulation of Wnt signaling pathway	0.000174984	Dkk2; Frzb; Six3	negative regulation of Wnt signaling pathway	Process
105	chr4_123572090_123581365_F	response to virus	0.000351989	Bcl3; Dhx58; Ifi27l2a	response to virus	Process
106	chr4_35106125_35127700_F	brush border membrane	0.000147259	Folr1; Pdzk1; Shank2; Slc34a3	brush border membrane	Component
107	chr4_35106125_35127700_F	regulation of body fluid levels	0.00046353	Apln; Aplnr	regulation of body fluid levels	Process
108	chr4_88745771_88776323_F	brush border membrane	0.000332299	Folr1; Lrp2; Pdzk1; Shank2	brush border membrane	Component

109	chr5_110182844_110228345_R	apical plasma membrane	0.000204999	Gp2; Lrp2; P2ry1; Pdzk1; Podxl; Rab17; Shank2; Slc34a3; Slc39a4	apical plasma membrane	Component
110	chr5_137392195_137426421_F	extracellular space	0.000175445	Angptl4; Anxa13; Apln; Ctsz; Cx3cl1; Dkk2; Fabp3; Frzb; Il1rn; Kng2; Lgals3bp; Lrg1; Npy; Pla2g7; Prtn3; S100b; Selp; Spp1; Vcam1	extracellular space	Component
111	chr5_149751979_149808240_F	apical plasma membrane	0.000213417	Gp2; Lrp2; Pdzk1; Podxl; Rab17; Shank2; Slc34a3; Slc39a4	apical plasma membrane	Component
112	chr5_31883451_31884082_F	brush border membrane	0.000350557	Folr1; Lrp2; Pdzk1; Shank2	brush border membrane	Component
113	chr5_35893908_35896613_R	apical plasma membrane	0.000113297	Gp2; Lrp2; P2ry1; Pdzk1; Podxl; Rab17; Shank2; Slc34a3; Slc39a4	apical plasma membrane	Component
114	chr5_35893908_35896613_R	transport	0.000272756	Abcb8; Abcg3; Apoc1; Aqp4; Atp5h; Chmp4c; Cpt2; Etfdh; Folr1; Grid2; Grik3; Kcnk4; Kcnq4; Mcoln3; Micu1; Pdzk1; Pex14; Pitpnc1; Rab17; Slc10a5; Slc17a9; Slc22a23; Slc25a1; Slc25a5; Slc34a3; Slc39a4; Slc6a7; Trpm3	transport	Process
115	chr5_35893908_35896613_R	mitochondrial inner membrane	0.000368306	Abcb8; Atp5h; Cox15; Cpt2; Etfdh; Gatm; Idh2; Micu1; Slc25a1; Slc25a5	mitochondrial inner membrane	Component
116	chr5_54053882_54054174_F	transport	0.000182655	Abcb8; Abcg3; Apoc1; Aqp4; Atp5h; Chmp4c; Cpt2; Etfdh; Grid2; Kcnk4; Kcnq4; Mcoln3; Pdzk1; Pex14; Pitpnc1; Rab17; Slc10a5; Slc17a9; Slc22a23; Slc25a42; Slc25a5; Slc34a3; Slc39a4; Slc6a7; Trpm3	transport	Process
117	chr6_37630192_37682692_R	receptor binding	0.000299096	Apln; Galp; Gdf5; Kng2; Pex14; S100b; Six3; Tg; Wnt8a	receptor binding	Function
118	chr6_47959825_47960502_R	transport	0.000138404	Abcb8; Abcg3; Aqp4; Atp5h; Chmp4c; Cpt2; Etfdh; Fabp3; Folr1; Grid2; Grik3; Kcnk4; Kcnq4; Mcoln3; Micu1; Mup20; Pdzk1; Pex14; Pitpnc1; Rab17; Slc10a5; Slc17a9; Slc22a23; Slc25a5; Slc39a4; Slc6a7; Trpm3	transport	Process
119	chr6_47959825_47960502_R	transporter activity	0.000267239	Aqp4; Fabp3; Grid2; Mup20; Slc17a9; Slc22a23; Slc25a5	transporter activity	Function
120	chr6_86425510_86450160_F	extracellular space	0.000187294	Angptl4; Ctsz; Cxcl1; Dkk2; Gdf5; Lrg1; Selp; Timp1; Vcam1	extracellular space	Component

121	chr6_86425510_86450160_F	response to virus	0.000216375	Bcl3; Dhx58; Ifi27l2a	response to virus	Process
122	chr7_133402766_133415516_R	mitochondrial inner membrane	0.000224283	Abcb8; Atp5h; Cox15; Cpt2; Etfdh; Gatm; Idh2; Micu1; Slc25a1; Slc25a5	mitochondrial inner membrane	Component
123	chr7_134372392_134372943_R	mitochondrial inner membrane	0.000175483	Abcb8; Atp5h; Cox15; Cpt2; Gatm; Idh2; Micu1; Slc25a1; Slc25a5	mitochondrial inner membrane	Component
124	chr7_134372392_134372943_R	apical plasma membrane	0.000498152	Gp2; Lrp2; P2ry1; Pdzk1; Podxl; Rab17; Slc39a4	apical plasma membrane	Component
125	chr7_26212709_26230775_F	receptor binding	0.000381473	Apln; Galp; Gdf5; Kng2; Pex14; S100b; Six3; Tg; Wnt8a	receptor binding	Function
126	chr7_31709550_31723025_F	transport	0.000187142	Abcb8; Abcg3; Apoc1; Aqp4; Atp5h; Chmp4c; Cpt2; Etfdh; Fabp3; Folr1; Grid2; Grik3; Kcnk4; Kcnq4; Mcoln3; Micu1; Pdzk1; Pex14; Pitpnc1; Rab17; Slc10a5; Slc17a9; Slc22a23; Slc25a1; Slc25a5; Slc39a4; Slc6a7; Trpm3	transport	Process
127	chr7_31709550_31723025_F	mitochondrial inner membrane	0.000308952	Abcb8; Atp5h; Cox15; Cpt2; Etfdh; Gatm; Idh2; Micu1; Slc25a1; Slc25a5	mitochondrial inner membrane	Component
128	chr7_82902248_82955248_F	mitochondrial inner membrane	0.000106263	Abcb8; Atp5h; Cpt2; Etfdh; Gatm; Idh2; Micu1; Slc25a1; Slc25a42; Slc25a5	mitochondrial inner membrane	Component
129	chr7_82902248_82955248_F	transport	0.000122237	Abcb8; Abcg3; Atp5h; Chmp4c; Cpt2; Etfdh; Fabp3; Folr1; Grik3; Kcnk4; Kcnq4; Mcoln3; Micu1; Mup20; Pdzk1; Pex14; Pitpnc1; Rab17; Slc10a5; Slc17a9; Slc25a1; Slc25a42; Slc25a5; Slc39a4; Slc6a7; Trpm3	transport	Process
130	chr7_96640618_96649018_F	mitochondrial inner membrane	0.000185336	Abcb8; Atp5h; Cox15; Cpt2; Etfdh; Gatm; Idh2; Micu1; Prkar2b; Slc25a5	mitochondrial inner membrane	Component
131	chr7_96640618_96649018_F	transport	0.000369785	Abcb8; Abcg3; Apoc1; Aqp4; Atp5h; Chmp4c; Cpt2; Etfdh; Grid2; Grik3; Kcnk4; Kcnq4; Mcoln3; Micu1; Pdzk1; Pex14; Pitpnc1; Rab17; Slc10a5; Slc17a9; Slc22a23; Slc25a5; Slc34a3; Slc39a4; Slc6a7; Trpm3	transport	Process
132	chr8_108153471_108159672_F	PDZ domain binding	0.000418354	Grid2; Kirrel3; Lrp2; Pdzk1; Ushbp1	PDZ domain binding	Function
133	chr8_108153471_108159672_F	receptor activity	0.000478696	Cd200r4; Folr1; Grid2; Grik3; Lrp2; Plxna4	receptor activity	Function

134	chr8_119732348_119749198_R	brush border membrane	0.000379277	Folr1; Lrp2; Pdzk1; Shank2	brush border membrane	Component
135	chr8_123901815_123902167_R	transport	0.000220669	Abcb8; Aqp4; Atp5h; Chmp4c; Cpt2; Etfdh; Fabp3; Grid2; Grik3; Kcnk4; Kcnq4; Mcoln3; Micu1; Mup20; Pdzk1; Pitpnc1; Rab17; Slc10a5; Slc25a1; Slc25a42; Slc25a5; Slc39a4; Slc6a7; Trpm3	transport	Process
136	chr8_126396600_126407275_R	receptor activity	0.000458996	Cd200r4; Folr1; Grid2; Grik3; Lrp2; Plxna4	receptor activity	Function
137	chr8_129102060_129109035_F	transport	0.000406931	Abcb8; Abcg3; Atp5h; Chmp4c; Cpt2; Etfdh; Fabp3; Folr1; Grid2; Grik3; Kcnk4; Kcnq4; Mcoln3; Micu1; Pdzk1; Pex14; Pitpnc1; Rab17; Slc10a5; Slc17a9; Slc39a4; Slc6a7; Trpm3	transport	Process
138	chr8_26345571_26346095_R	apical plasma membrane	0.000213417	Gp2; Lrp2; Pdzk1; Podxl; Rab17; Shank2; Slc34a3; Slc39a4	apical plasma membrane	Component
139	chr8_48476637_48492537_F	extracellular space	0.000191639	Anxa13; Apln; Cx3cl1; Cxcl1; Dkk2; Frzb; Ggt1; Il1rn; Lgals3bp; Lrg1; Npy; Prtn3; S100b; Selp; Spp1; Tg; Timp1; Vcam1	extracellular space	Component
140	chr8_48476637_48492537_R	IPAF inflammasome complex	0.000121578	Casp4; Naip2	IPAF inflammasome complex	Component
141	chr8_48476637_48492537_R	pyroptosis	0.000226102	Casp4; Naip2	pyroptosis	Process
142	chr8_48476637_48492537_R	extracellular space	0.000311429	Angptl4; Ctsz; Cx3cl1; Dkk2; Frzb; Lgals3bp; Lrg1; Pla2g7; Selp; Spp1; Vcam1	extracellular space	Component
143	chr8_96983590_96994419_F	IPAF inflammasome complex	0.000230723	Casp4; Naip2	IPAF inflammasome complex	Component
144	chr8_96983590_96994419_F	amine metabolic process	0.000230723	Cyp1a1; Vcam1	amine metabolic process	Process
145	chr8_96983590_96994419_F	dipeptidyl-peptidase activity	0.000322178	Dpep2; Dpp10	dipeptidyl-peptidase activity	Function
146	chr8_96983590_96994419_F	immune system process	0.00035114	Cd180; Cd300ld; Clec7a; Dhx58; Mefv; Naip2; Themis2	immune system process	Process
147	chr8_96983590_96994419_F	pyroptosis	0.000428461	Casp4; Naip2	pyroptosis	Process
148	chr8_96983590_96994419_F	neutrophil chemotaxis	0.000496914	Cx3cl1; Cxcl1; Spp1	neutrophil chemotaxis	Process

149	chr9_121845920_121858120_F	transport	0.000153241	Abcb8; Abcg3; Apoc1; Aqp4; Atp5h; Chmp4c; Cpt2; Etfdh; Grid2; Grik3; Kcnk4; Kcnq4; Mcoln3; Micu1; Pdzk1; Pex14; Pitpnc1; Rab17; Slc10a5; Slc17a9; Slc22a23; Slc25a1; Slc25a5; Slc34a3; Slc39a4; Slc6a7; Trpm3	transport	Process
150	chr9_121845920_121858120_F	mitochondrial inner membrane	0.000185336	Abcb8; Atp5h; Cox15; Cpt2; Etfdh; Gatm; Idh2; Micu1; Slc25a1; Slc25a5	mitochondrial inner membrane	Component
151	chr9_121845920_121858120_F	apical plasma membrane	0.000337522	Gp2; Lrp2; Pdzk1; Podxl; Rab17; Shank2; Slc34a3; Slc39a4	apical plasma membrane	Component
152	chr9_14480441_14483362_F	apical plasma membrane	0.000222857	Gp2; Lrp2; Pdzk1; Podxl; Rab17; Shank2; Slc34a3; Slc39a4	apical plasma membrane	Component
153	chr9_27148989_27155714_R	fatty acid beta-oxidation using acyl-CoA dehydrogenase	0.000207069	Acad11; Etfdh	fatty acid beta-oxidation using acyl-CoA dehydrogenase	Process
154	chr9_27148989_27155714_R	mitochondrion	0.000292908	Abcb8; Acad11; Adhfe1; Atp5h; Chchd10; Cox15; Cpt2; Etfdh; Gatm; Hspe1; Idh2; P2ry1; Slc25a1; Slc25a42; Slc25a5; Xaf1	mitochondrion	Component
155	chr9_27148989_27155714_R	transport	0.000367971	Abcb8; Atp5h; Chmp4c; Cpt2; Etfdh; Fabp3; Grid2; Kcnk4; Mcoln3; Mup20; Pdzk1; Pitpnc1; Rab17; Slc25a1; Slc25a42; Slc25a5; Trpm3	transport	Process
156	chr9_27155685_27156100_F	transport	0.000316186	Chmp4c; Cpt2; Etfdh; Fabp3; Grid2; Kcnk4; Mup20; Pitpnc1; Rab17; Slc25a1; Slc25a42; Slc25a5; Trpm3	transport	Process
157	chr9_4260788_4261404_F	receptor activity	0.000190134	Cd200r4; Folr1; Grid2; Grik3; Lrp2; Plxna4	receptor activity	Function
158	chr9_56646768_56649068_R	transport	0.000185805	Abcb8; Abcg3; Apoc1; Atp5h; Chmp4c; Cpt2; Folr1; Grid2; Grik3; Kcnk4; Kcnq4; Mcoln3; Micu1; Mup20; Pdzk1; Pex14; Pitpnc1; Rab17; Slc10a5; Slc17a9; Slc22a23; Slc25a1; Slc25a5; Slc39a4; Slc6a7; Trpm3	transport	Process
159	chr9_68349343_68411643_R	PDZ domain binding	0.000465558	Grid2; Kirrel3; Lrp2; Pdzk1; Ushbp1	PDZ domain binding	Function

160	chrX_102568332_102587093_R	brush border membrane	0.000314733	Lrp2; Pdzk1; Shank2; Slc34a3	brush border membrane	Component
161	chrX_165792277_165807952_R	brush border membrane	0.000106455	Folr1; Lrp2; Pdzk1; Shank2; Slc34a3	brush border membrane	Component
162	chrX_165792277_165807952_R	transport	0.000113935	Abcb8; Abcg3; Apoc1; Aqp4; Atp5h; Chmp4c; Cpt2; Etfdh; Fabp3; Folr1; Grid2; Grik3; Kcnk4; Kcnq4; Mcoln3; Micu1; Mup20; Pdzk1; Pex14; Pitpnc1; Rab17; Slc10a5; Slc17a9; Slc22a23; Slc25a1; Slc25a42; Slc25a5; Slc34a3; Slc39a4; Slc6a7; Trpm3	transport	Process
163	chrX_165792277_165807952_R	mitochondrial inner membrane	0.000187108	Abcb8; Atp5h; Cox15; Cpt2; Etfdh; Gatm; Idh2; Micu1; Slc25a1; Slc25a42; Slc25a5	mitochondrial inner membrane	Component
164	chrX_165792277_165807952_R	apical plasma membrane	0.000229265	Gp2; Lrp2; P2ry1; Pdzk1; Podxl; Rab17; Shank2; Slc34a3; Slc39a4	apical plasma membrane	Component
165	chrX_18734106_18744367_R	brush border membrane	0.000359951	Lrp2; Pdzk1; Shank2; Slc34a3	brush border membrane	Component
166	ENSMUST00000125309	brush border membrane	0.000111518	Folr1; Lrp2; Pdzk1; Shank2; Slc34a3	brush border membrane	Component
167	ENSMUST00000125309	transport	0.00013708	Abcb8; Abcg3; Apoc1; Aqp4; Atp5h; Chmp4c; Cpt2; Etfdh; Fabp3; Folr1; Grid2; Grik3; Kcnk4; Kcnq4; Mcoln3; Micu1; Mup20; Pdzk1; Pex14; Pitpnc1; Rab17; Slc10a5; Slc17a9; Slc22a23; Slc25a1; Slc25a42; Slc25a5; Slc34a3; Slc39a4; Slc6a7; Trpm3	transport	Process
168	ENSMUST00000125309	mitochondrial inner membrane	0.000203919	Abcb8; Atp5h; Cox15; Cpt2; Etfdh; Gatm; Idh2; Micu1; Slc25a1; Slc25a42; Slc25a5	mitochondrial inner membrane	Component
169	ENSMUST00000125309	apical plasma membrane	0.000246731	Gp2; Lrp2; P2ry1; Pdzk1; Podxl; Rab17; Shank2; Slc34a3; Slc39a4	apical plasma membrane	Component
170	ENSMUST00000130677	PDZ domain binding	0.000450599	Frzb; Grid2; Kirrel3; Lrp2; Pdzk1; Ushbp1	PDZ domain binding	Function
171	ENSMUST00000141575	transport	0.000154079	Abcb8; Abcg3; Apoc1; Aqp4; Atp5h; Chmp4c; Cpt2; Etfdh; Folr1; Grid2; Grik3; Kcnk4; Kcnq4; Mcoln3; Micu1; Pdzk1; Pex14; Pitpnc1; Rab17; Slc10a5; Slc17a9; Slc22a23; Slc25a1; Slc25a5; Slc34a3; Slc39a4; Slc6a7; Trpm3	transport	Process

172	ENSMUST00000141575	mitochondrial inner membrane	0.000282414	Abcb8; Atp5h; Cox15; Cpt2; Etfdh; Gatm; Idh2; Micu1; Slc25a1; Slc25a5	mitochondrial inner membrane	Component
173	ENSMUST00000141575	apical plasma membrane	0.000478122	Gp2; Lrp2; Pdzk1; Podxl; Rab17; Shank2; Slc34a3; Slc39a4	apical plasma membrane	Component
174	ENSMUST00000149707	amine metabolic process	0.000211698	Cyp1a1; Vcam1	amine metabolic process	Process
175	ENSMUST00000149707	IPAF inflammasome complex	0.000211698	Casp4; Naip2	IPAF inflammasome complex	Component
176	ENSMUST00000149707	pyroptosis	0.000393218	Casp4; Naip2	pyroptosis	Process
177	ENSMUST00000149707	negative regulation of interleukin-1 beta production	0.000504317	Acp5; Mefv	negative regulation of interleukin-1 beta production	Process
178	ENSMUST00000150216	brush border membrane	0.000487198	Folr1; Lrp2; Pdzk1; Shank2	brush border membrane	Component
179	ENSMUST00000155173	brush border membrane	0.000306205	Lrp2; Pdzk1; Shank2; Slc34a3	brush border membrane	Component
180	ENSMUST00000157205	apical plasma membrane	0.000275339	Gp2; Lrp2; Pdzk1; Podxl; Rab17; Shank2; Slc34a3; Slc39a4	apical plasma membrane	Component
181	ENSMUST00000158863	brush border membrane	0.000289647	Folr1; Lrp2; Pdzk1; Shank2	brush border membrane	Component
182	ENSMUST00000163396	brush border membrane	0.000441707	Folr1; Lrp2; Pdzk1; Shank2	brush border membrane	Component
183	ENSMUST00000174110	transport	0.000185805	Abcb8; Abcg3; Apoc1; Aqp4; Atp5h; Chmp4c; Cpt2; Etfdh; Fabp3; Grid2; Grik3; Kcnk4; Kcnq4; Mcoln3; Micu1; Pdzk1; Pitpnc1; Rab17; Slc10a5; Slc17a9; Slc25a1; Slc25a42; Slc25a5; Slc39a4; Slc6a7; Trpm3	transport	Process
184	ENSMUST00000180590	regulation of body fluid levels	0.000400935	Apln; Aplnr	regulation of body fluid levels	Process
185	ENSMUST00000181887	brush border membrane	0.000389213	Folr1; Lrp2; Shank2; Slc34a3	brush border membrane	Component
186	FR000724	regulation of body fluid levels	0.000431674	Apln; Aplnr	regulation of body fluid levels	Process
187	FR000826	IPAF inflammasome complex	0.000107841	Casp4; Naip2	IPAF inflammasome complex	Component
188	FR000826	pyroptosis	0.000200599	Casp4; Naip2	pyroptosis	Process
189	FR000826	negative regulation of interleukin-1 beta production	0.000257462	Acp5; Mefv	negative regulation of interleukin-1 beta production	Process
190	FR000826	immune system process	0.000275826	Cd300ld; Clec7a; Dhx58; Mefv; Naip2; Themis2	immune system process	Process

191	FR000826	extracellular vesicular exosome	0.000397457	Chmp4c; Cpn2; Ctsz; Fgl1; Ftl1; Gatm; Il1rn; Lgals3bp; Lrg1; Naip2; Rap2c; Rnf149; Sirpb1a; Slc25a5; Spp1; Vcam1	extracellular vesicular exosome	Component
192	FR000826	cell adhesion	0.00045945	Cx3cl1; Lgals3bp; Nrp2; Sdc3; Selp; Spp1; Vcam1	cell adhesion	Process
193	FR000826	negative regulation of interleukin-12 production	0.00046954	Acp5; Mefv	negative regulation of interleukin-12 production	Process
194	FR001084	brush border membrane	0.00017373	Lrp2; Pdzk1; Shank2; Slc34a3	brush border membrane	Component
195	FR001084	regulation of body fluid levels	0.000504919	Apln; Aplnr	regulation of body fluid levels	Process
196	FR001172	mitochondrial inner membrane	0.000230201	Abcb8; Atp5h; Cox15; Cpt2; Gatm; Idh2; Micu1; Slc25a1; Slc25a5	mitochondrial inner membrane	Component
197	FR003529	apical plasma membrane	0.000162837	Gp2; Lrp2; P2ry1; Pdzk1; Podxl; Rab17; Shank2; Slc34a3; Slc39a4	apical plasma membrane	Component
198	FR003529	transport	0.000268021	Abcb8; Abcg3; Apoc1; Aqp4; Atp5h; Chmp4c; Cpt2; Etfdh; Fabp3; Grid2; Grik3; Kcnk4; Kcnq4; Mcoln3; Micu1; Pdzk1; Pex14; Pitpnc1; Rab17; Slc10a5; Slc17a9; Slc22a23; Slc25a1; Slc25a42; Slc25a5; Slc34a3; Slc39a4; Slc6a7; Trpm3	transport	Process
199	FR003529	PDZ domain binding	0.000382745	Frzb; Grid2; Kirrel3; Lrp2; Pdzk1; Ushbp1	PDZ domain binding	Function
200	FR007551	brush border membrane	0.00013071	Folr1; Lrp2; Pdzk1; Shank2; Slc34a3	brush border membrane	Component
201	FR007551	apical plasma membrane	0.000316794	Gp2; Lrp2; P2ry1; Pdzk1; Podxl; Rab17; Shank2; Slc34a3; Slc39a4	apical plasma membrane	Component
202	FR007551	extracellular space	0.000318464	Angptl4; Apln; Bpifa1; Ctsz; Cxcl1; Dkk2; Gdf5; Ggt1; Il1rn; Kng2; Lgals3bp; Lrg1; Lrp2; Npy; Pigr; Pla2g7; Prtn3; S100b; Selp; Tg; Timp1; Vcam1	extracellular space	Component
203	FR010306	mitochondrial inner membrane	0.000298388	Abcb8; Atp5h; Cox15; Cpt2; Gatm; Idh2; Micu1; Slc25a1; Slc25a5	mitochondrial inner membrane	Component

204	FR016598	transport	0.000138404	Abcb8; Abcg3; Apoc1; Aqp4; Atp5h; Chmp4c; Cpt2; Etfdh; Fabp3; Folr1; Grid2; Grik3; Kcnk4; Kcnq4; Mcoln3; Micu1; Pdzk1; Pex14; Pitpnc1; Rab17; Slc10a5; Slc17a9; Slc22a23; Slc25a5; Slc39a4; Slc6a7; Trpm3	transport	Process
205	FR017535	transporter activity	0.000257636	Aqp4; Fabp3; Grid2; Mup20; Slc17a9; Slc22a23; Slc25a5	transporter activity	Function
206	FR028133	mitochondrion	0.000226421	Adhfe1; Cox15; Cpt2; Cyp1a1; Etfdh; Gatm; Hspe1; Idh2; Micu1; P2ry1; Slc25a1; Slc25a42; Slc25a5; Snph	mitochondrion	Component
207	FR028809	mitochondrial inner membrane	0.000463055	Abcb8; Atp5h; Cox15; Cpt2; Gatm; Idh2; Micu1; Slc25a1; Slc25a5	mitochondrial inner membrane	Component
208	FR029539	IPAF inflammasome complex	0.000189057	Casp4; Naip2	IPAF inflammasome complex	Component
209	FR029539	pyroptosis	0.000351261	Casp4; Naip2	pyroptosis	Process
210	FR029539	external side of plasma membrane	0.000364487	Aqp4; Clec7a; Ly6g; Selp; Slamf7; Vcam1	external side of plasma membrane	Component
211	FR029539	negative regulation of interleukin-1 beta production	0.000450568	Acp5; Aqp4	negative regulation of interleukin-1 beta production	Process
212	FR032503	PDZ domain binding	0.000310062	Grid2; Kirrel3; Lrp2; Pdzk1; Ushbp1	PDZ domain binding	Function
213	FR032503	receptor activity	0.000338156	Cd200r4; Folr1; Grid2; Grik3; Lrp2; Plxna4	receptor activity	Function
214	FR032503	regulation of body fluid levels	0.000504919	Apln; Aplnr	regulation of body fluid levels	Process
215	FR034857	apical plasma membrane	0.000284755	Gp2; Lrp2; P2ry1; Pdzk1; Podxl; Rab17; Slc39a4	apical plasma membrane	Component
216	FR034857	PDZ domain binding	0.000374856	Grid2; Kirrel3; Lrp2; Pdzk1; Ushbp1	PDZ domain binding	Function
217	FR034857	receptor activity	0.000421496	Cd200r4; Folr1; Grid2; Grik3; Lrp2; Plxna4	receptor activity	Function
218	FR034857	mitochondrial inner membrane	0.000474306	Abcb8; Cox15; Cpt2; Gatm; Idh2; Micu1; Slc25a1; Slc25a5	mitochondrial inner membrane	Component
219	FR042390	apical plasma membrane	0.000455748	Gp2; Lrp2; P2ry1; Pdzk1; Podxl; Rab17; Slc39a4	apical plasma membrane	Component
220	FR044110	IPAF inflammasome complex	0.000132417	Casp4; Naip2	IPAF inflammasome complex	Component
221	FR044110	pyroptosis	0.000246218	Casp4; Naip2	pyroptosis	Process
222	FR044110	extracellular space	0.000454788	Angptl4; Ctsz; Cx3cl1; Dkk2; Frzb; Lgals3bp; Lrg1; Pla2g7; Selp; Spp1; Vcam1	extracellular space	Component

223	FR046015	negative regulation of interleukin-1 beta production	0.00034282	Acp5; Mefv	negative regulation of interleukin-1 beta production	Process
224	FR047247	PDZ domain binding	0.00016942	Frzb; Grid2; Kirrel3; Lrp2; Pdzk1; Ushbp1	PDZ domain binding	Function
225	FR047247	apical plasma membrane	0.000298978	Gp2; Lrp2; Pdzk1; Podxl; Rab17; Shank2; Slc34a3; Slc39a4	apical plasma membrane	Component
226	FR049065	mitochondrial inner membrane	0.000255729	Abcb8; Atp5h; Cox15; Cpt2; Etfdh; Gatm; Idh2; Micu1; Slc25a42	mitochondrial inner membrane	Component
227	FR052210	PDZ domain binding	0.000347818	Grid2; Kirrel3; Lrp2; Pdzk1; Ushbp1	PDZ domain binding	Function
228	FR052210	receptor activity	0.000386432	Cd200r4; Folr1; Grid2; Grik3; Lrp2; Plxna4	receptor activity	Function
229	FR054246	apical plasma membrane	0.000496305	Lrp2; P2ry1; Pdzk1; Podxl; Rab17; Shank2; Slc34a3; Slc39a4	apical plasma membrane	Component
230	FR055065	PDZ domain binding	0.000279291	Frzb; Grid2; Kirrel3; Lrp2; Pdzk1; Ushbp1	PDZ domain binding	Function
231	FR055102	transport	0.000491914	Abcb8; Abcg3; Apoc1; Aqp4; Atp5h; Chmp4c; Cpt2; Etfdh; Fabp3; Folr1; Grid2; Grik3; Kcnk4; Kcnq4; Mcoln3; Micu1; Mup20; Pdzk1; Pex14; Pitpnc1; Rab17; Slc10a5; Slc17a9; Slc22a23; Slc25a1; Slc25a5; Slc39a4; Slc6a7; Trpm3	transport	Process
232	FR057053	brush border membrane	0.000399334	Folr1; Lrp2; Pdzk1; Shank2	brush border membrane	Component
233	FR057875	regulation of body fluid levels	0.000328987	Apln; Aplnr	regulation of body fluid levels	Process
234	FR060087	extracellular space	0.000123162	Angptl4; Apln; Ctsz; Cx3cl1; Dkk2; Frzb; Il1rn; Lgals3bp; Lrg1; Npy; Pla2g7; S100b; Selp; Spp1; Vcam1	extracellular space	Component
235	FR060087	fatty acid beta-oxidation using acyl-CoA dehydrogenase	0.00029265	Acad11; Etfdh	fatty acid beta-oxidation using acyl-CoA dehydrogenase	Process
236	FR060087	IPAF inflammasome complex	0.00029265	Casp4; Naip2	IPAF inflammasome complex	Component

237	FR061730	transport	0.000185805	Abcb8; Abcg3; Apoc1; Aqp4; Atp5h; Chmp4c; Cpt2; Etfdh; Fabp3; Folr1; Grid2; Grik3; Kcnk4; Kcnq4; Mcoln3; Micu1; Pdzk1; Pex14; Pitpnc1; Rab17; Slc10a5; Slc17a9; Slc22a23; Slc39a4; Slc6a7; Trpm3	transport	Process
238	FR064590	brush border membrane	0.000323431	Lrp2; Pdzk1; Shank2; Slc34a3	brush border membrane	Component
239	FR064953	mitochondrial inner membrane	0.000100922	Atp5h; Cpt2; Etfdh; Gatm; Prkar2b; Slc25a1; Slc25a42; Slc25a5	mitochondrial inner membrane	Component
240	FR064953	fatty acid beta-oxidation using acyl-CoA dehydrogenase	0.000250557	Acad11; Etfdh	fatty acid beta-oxidation using acyl-CoA dehydrogenase	Process
241	FR064953	IPAF inflammasome complex	0.000250557	Casp4; Naip2	IPAF inflammasome complex	Component
242	FR064953	immune system process	0.00045141	Cd180; Cd300ld; Clec7a; Dhx58; Mefv; Naip2; Themis2	immune system process	Process
243	FR064953	pyroptosis	0.000465191	Casp4; Naip2	pyroptosis	Process
244	FR065159	regulation of body fluid levels	0.000504919	Apln; Aplnr	regulation of body fluid levels	Process
245	FR071800	fatty acid beta-oxidation using acyl-CoA dehydrogenase	0.000111199	Acad11; Etfdh	fatty acid beta-oxidation using acyl-CoA dehydrogenase	Process
246	FR074898	PDZ domain binding	0.000388956	Grid2; Kirrel3; Lrp2; Pdzk1; Ushbp1	PDZ domain binding	Function
247	FR074898	receptor activity	0.000439935	Cd200r4; Folr1; Grid2; Grik3; Lrp2; Plxna4	receptor activity	Function
248	FR082156	extracellular space	0.000115289	Angptl4; Anxa13; Apln; Ctsz; Cx3cl1; Dkk2; Frzb; Ggt1; Il1rn; Kng2; Lgals3bp; Npy; Pigr; Pla2g7; Prtn3; S100b; Selp; Spp1; Vcam1; Wnt8a	extracellular space	Component
249	FR082156	mitochondrial inner membrane	0.000194494	Abcb8; Atp5h; Cox15; Cpt2; Etfdh; Gatm; Idh2; Prkar2b; Slc25a42; Slc25a5	mitochondrial inner membrane	Component
250	FR085857	transcytosis	0.000290161	Rab17; Tg	transcytosis	Process
251	FR086793	mitochondrial inner membrane	0.000380605	Abcb8; Cox15; Cpt2; Gatm; Idh2; Micu1; Slc25a1; Slc25a5	mitochondrial inner membrane	Component
252	FR087537	mitochondrial inner membrane	0.000337542	Abcb8; Atp5h; Cox15; Cpt2; Etfdh; Gatm; Idh2; Micu1; Slc25a42; Slc25a5	mitochondrial inner membrane	Component

253	FR087537	transporter activity	0.000434649	Aqp4; Fabp3; Grid2; Mup20; Slc17a9; Slc22a23; Slc25a5	transporter activity	Function
254	FR087735	brush border membrane	0.000464055	Folr1; Lrp2; Pdzk1; Shank2	brush border membrane	Component
255	FR087925	mitochondrial inner membrane	0.000117411	Abcb8; Cox15; Cpt2; Etfdh; Gatm; Idh2; Micu1; Slc25a1; Slc25a5	mitochondrial inner membrane	Component
256	FR087925	apical plasma membrane	0.000362234	Gp2; Lrp2; P2ry1; Pdzk1; Podxl; Rab17; Slc39a4	apical plasma membrane	Component
257	FR087925	PDZ domain binding	0.0004494	Grid2; Kirrel3; Lrp2; Pdzk1; Ushbp1	PDZ domain binding	Function
258	FR090778	amine metabolic process	0.000143714	Cyp1a1; Vcam1	amine metabolic process	Process
259	FR090778	IPAF inflammasome complex	0.000143714	Casp4; Naip2	IPAF inflammasome complex	Component
260	FR090778	pyroptosis	0.00026718	Casp4; Naip2	pyroptosis	Process
261	FR090778	negative regulation of interleukin-1 beta production	0.00034282	Acp5; Mefv	negative regulation of interleukin-1 beta production	Process
262	FR090859	PDZ domain binding	0.000334867	Grid2; Kirrel3; Lrp2; Pdzk1; Ushbp1	PDZ domain binding	Function
263	FR090859	receptor activity	0.000369777	Cd200r4; Folr1; Grid2; Grik3; Lrp2; Plxna4	receptor activity	Function
264	FR099681	receptor activity	0.000458996	Cd200r4; Folr1; Grid2; Grik3; Lrp2; Plxna4	receptor activity	Function
265	FR100205	fatty acid beta-oxidation using acyl-CoA dehydrogenase	0.000132417	Acad11; Etfdh	fatty acid beta-oxidation using acyl-CoA dehydrogenase	Process
266	FR106089	IPAF inflammasome complex	0.000114608	Casp4; Naip2	IPAF inflammasome complex	Component
267	FR106089	extracellular vesicular exosome	0.000178643	Chmp4c; Cpn2; Ctsz; Fgl1; Ftl1; Il1rn; Lgals3bp; Lrg1; Naip2; Rab17; Rap2c; Rnf149; Sirpb1a; Slc25a5; Spp1; Timp1; Vcam1	extracellular vesicular exosome	Component
268	FR106089	pyroptosis	0.000213162	Casp4; Naip2	pyroptosis	Process
269	FR106089	extracellular space	0.000238752	Angptl4; Ctsz; Cxcl1; Il1rn; Lgals3bp; Lrg1; Pla2g7; Selp; Spp1; Timp1; Vcam1	extracellular space	Component
270	FR112324	brush border membrane	0.000399334	Folr1; Lrp2; Pdzk1; Shank2	brush border membrane	Component
271	FR114000	PDZ domain binding	0.000347818	Grid2; Kirrel3; Lrp2; Pdzk1; Ushbp1	PDZ domain binding	Function
272	FR114000	receptor activity	0.000386432	Cd200r4; Folr1; Grid2; Grik3; Lrp2; Plxna4	receptor activity	Function
273	FR117744	apical plasma membrane	0.000435655	Gp2; Lrp2; P2ry1; Pdzk1; Podxl; Rab17; Slc39a4	apical plasma membrane	Component

274	FR117799	transport	0.000266348	Abcb8; Abcg3; Atp5h; Chmp4c; Cpt2; Fabp3; Folr1; Grid2; Grik3; Kcnk4; Kcnq4; Mcoln3; Micu1; Mup20; Pdzk1; Pitpnc1; Rab17; Slc10a5; Slc17a9; Slc25a5; Slc39a4; Slc6a7; Trpm3	transport	Process
275	FR120704	mitochondrial inner membrane	0.000230201	Abcb8; Atp5h; Cox15; Cpt2; Gatm; Idh2; Micu1; Slc25a1; Slc25a5	mitochondrial inner membrane	Component
276	FR120704	transport	0.00036677	Abcb8; Abcg3; Apoc1; Atp5h; Chmp4c; Cpt2; Folr1; Grid2; Grik3; Kcnk4; Kcnq4; Mcoln3; Micu1; Pdzk1; Pitpnc1; Rab17; Slc10a5; Slc17a9; Slc25a1; Slc25a5; Slc39a4; Slc6a7; Trpm3	transport	Process
277	FR124060	regulation of body fluid levels	0.000289187	Apln; Aplnr	regulation of body fluid levels	Process
278	FR124060	receptor binding	0.000306791	Apln; Galp; Kng2; Pex14; S100b; Six3; Wnt8a	receptor binding	Function
279	FR125080	receptor binding	0.000439296	Apln; Galp; Gdf5; Kng2; Pex14; S100b; Six3; Tg; Wnt8a	receptor binding	Function
280	FR129526	brush border membrane	0.000266031	Lrp2; Pdzk1; Shank2; Slc34a3	brush border membrane	Component
281	FR139436	apical plasma membrane	0.000498152	Gp2; Lrp2; P2ry1; Pdzk1; Podxl; Rab17; Slc39a4	apical plasma membrane	Component
282	FR142459	fatty acid beta-oxidation using acyl-CoA dehydrogenase	0.000147582	Acad11; Etfdh	fatty acid beta-oxidation using acyl-CoA dehydrogenase	Process
283	FR142459	transport	0.000147794	Abcb8; Atp5h; Chmp4c; Cpt2; Etfdh; Fabp3; Grid2; Kcnk4; Mcoln3; Mup20; Pitpnc1; Rab17; Slc25a1; Slc25a42; Slc25a5; Trpm3	transport	Process
284	FR142459	semaphorin receptor activity	0.000439117	Nrp2; Plxna4	semaphorin receptor activity	Function
285	FR149067	PDZ domain binding	0.000361146	Grid2; Kirrel3; Lrp2; Pdzk1; Ushbp1	PDZ domain binding	Function
286	FR149067	receptor activity	0.000403666	Cd200r4; Folr1; Grid2; Grik3; Lrp2; Plxna4	receptor activity	Function
287	FR150621	receptor binding	0.000127303	Apln; Galp; Gdf5; Kng2; Pex14; S100b; Six3; Tg; Wnt8a	receptor binding	Function
288	FR152607	brush border membrane	0.000420139	Folr1; Lrp2; Shank2; Slc34a3	brush border membrane	Component
289	FR155648	fatty acid beta-oxidation using acyl-CoA dehydrogenase	0.000216379	Acad11; Etfdh	fatty acid beta-oxidation using acyl-CoA dehydrogenase	Process

290	FR155648	regulation of body fluid levels	0.000302172	Apln; Aplnr	regulation of body fluid levels	Process
291	FR160504	apical plasma membrane	0.000410708	Gp2; Lrp2; P2ry1; Pdzk1; Podxl; Rab17; Shank2; Slc39a4	apical plasma membrane	Component
292	FR167447	IPAF inflammasome complex	0.00029265	Casp4; Naip2	IPAF inflammasome complex	Component
293	FR167447	fatty acid beta-oxidation using acyl-CoA dehydrogenase	0.00029265	Acad11; Etfdh	fatty acid beta-oxidation using acyl-CoA dehydrogenase	Process
294	FR168142	PDZ domain binding	0.000347818	Grid2; Kirrel3; Lrp2; Pdzk1; Ushbp1	PDZ domain binding	Function
295	FR168142	receptor activity	0.000386432	Cd200r4; Folr1; Grid2; Grik3; Lrp2; Plxna4	receptor activity	Function
296	FR168758	regulation of body fluid levels	0.000423885	Apln; Aplnr	regulation of body fluid levels	Process
297	FR177295	PDZ domain binding	0.000138449	Frzb; Grid2; Kirrel3; Lrp2; Pdzk1; Ushbp1	PDZ domain binding	Function
298	FR180118	IPAF inflammasome complex	0.000101279	Casp4; Naip2	IPAF inflammasome complex	Component
299	FR180118	extracellular space	0.000135465	Angptl4; Ctsz; Cx3cl1; Dkk2; Frzb; Lgals3bp; Lrg1; Pla2g7; Selp; Spp1; Vcam1	extracellular space	Component
300	FR180118	pyroptosis	0.000188414	Casp4; Naip2	pyroptosis	Process
301	FR180118	cell adhesion	0.000378716	Cx3cl1; Lgals3bp; Nrp2; Sdc3; Selp; Spp1; Vcam1	cell adhesion	Process
302	FR184042	extracellular region	0.000462451	1700029I15Rik; Angptl4; Apoc1; Bpifa1; Cma1; Cpn2; Cx3cl1; Cxcl1; Defb28; Dkk2; Fgl1; Frzb; Galp; Gdf5; Il1rn; Lgals3bp; Lipf; Mup20; Npy; Pla2g10; Pla2g7; S100b; Spp1; Tg; Timp1; Wnt8a	extracellular region	Component
303	FR184042	inflammatory response	0.000503796	Cd180; Clec7a; Cxcl1; Mefv; Naip2; Pla2g7; Selp; Themis2	inflammatory response	Process
304	FR187810	transport	0.000335348	Abcb8; Abcg3; Atp5h; Chmp4c; Cpt2; Etfdh; Fabp3; Folr1; Grid2; Grik3; Kcnk4; Mcoln3; Micu1; Mup20; Pdzk1; Pitpnc1; Rab17; Slc10a5; Slc17a9; Slc25a1; Slc25a5; Slc39a4; Slc6a7; Trpm3	transport	Process

305	FR194887	extracellular vesicular exosome	0.000254564	Chmp4c; Cnksr2; Cpn2; Dpp6; Fgl1; Ftl1; Gatm; Il1rn; Lgals3bp; Lrg1; Naip2; Pdzk1; Phgdh; Prtn3; Rab17; Rap2c; Rnf149; Serinc2; Sirpb1a; Slc25a5; Spp1; Timp1; Vcam1	extracellular vesicular exosome	Component
306	FR194887	IPAF inflammasome complex	0.000298138	Casp4; Naip2	IPAF inflammasome complex	Component
307	FR194887	amine metabolic process	0.000298138	Cyp1a1; Vcam1	amine metabolic process	Process
308	FR194887	regulation of body fluid levels	0.000416165	Apln; Aplnr	regulation of body fluid levels	Process
309	FR199374	brush border membrane	0.000350557	Folr1; Lrp2; Pdzk1; Shank2	brush border membrane	Component
310	FR202182	PDZ domain binding	0.000334867	Grid2; Kirrel3; Lrp2; Pdzk1; Ushbp1	PDZ domain binding	Function
311	FR202182	receptor activity	0.000369777	Cd200r4; Folr1; Grid2; Grik3; Lrp2; Plxna4	receptor activity	Function
312	FR203009	apical plasma membrane	0.000232636	Gp2; Lrp2; P2ry1; Pdzk1; Podxl; Rab17; Shank2; Slc39a4	apical plasma membrane	Component
313	FR204953	apical plasma membrane	0.000380026	Gp2; Lrp2; P2ry1; Pdzk1; Podxl; Rab17; Shank2; Slc39a4	apical plasma membrane	Component
314	FR215148	regulation of body fluid levels	0.000496502	Apln; Aplnr	regulation of body fluid levels	Process
315	FR215734	mitochondrial inner membrane	0.000124272	Abcb8; Atp5h; Cox15; Cpt2; Etfdh; Gatm; Idh2; Micu1; Slc25a1; Slc25a5	mitochondrial inner membrane	Component
316	FR215734	transport	0.000167639	Abcb8; Abcg3; Apoc1; Atp5h; Chmp4c; Cpt2; Etfdh; Fabp3; Folr1; Grid2; Grik3; Kcnk4; Kcnq4; Mcoln3; Micu1; Mup20; Pdzk1; Pitpnc1; Rab17; Slc10a5; Slc17a9; Slc25a1; Slc25a5; Slc39a4; Slc6a7; Trpm3	transport	Process
317	FR219193	apical plasma membrane	0.000237865	Gp2; Lrp2; P2ry1; Pdzk1; Podxl; Rab17; Shank2; Slc34a3; Slc39a4	apical plasma membrane	Component
318	FR230183	transport	0.000245488	Abcb8; Abcg3; Aqp4; Atp5h; Chmp4c; Cpt2; Etfdh; Fabp3; Folr1; Grid2; Grik3; Kcnk4; Kcnq4; Mcoln3; Micu1; Pdzk1; Pex14; Pitpnc1; Rab17; Slc10a5; Slc17a9; Slc39a4; Slc6a7; Trpm3	transport	Process
319	FR234548	brush border membrane	0.000289647	Folr1; Lrp2; Pdzk1; Shank2	brush border membrane	Component

320	FR237950	PDZ domain binding	0.00012919	Frzb; Grid2; Kirrel3; Lrp2; Pdzk1; Ushbp1	PDZ domain binding	Function
321	FR238898	mitochondrial inner membrane	0.000128161	Abcb8; Atp5h; Cox15; Cpt2; Etfdh; Gatm; Idh2; Micu1; Prkar2b; Slc25a1; Slc25a42; Slc25a5	mitochondrial inner membrane	Component
322	FR238898	brush border membrane	0.000184056	Folr1; Lrp2; Pdzk1; Shank2; Slc34a3	brush border membrane	Component
323	FR238898	extracellular region	0.000435703	Abhd15; Angptl4; Apoc1; Bpifa1; Cma1; Cpn2; Cxcl1; Defb28; Dkk2; Fgl1; Folr1; Frzb; Galp; Gdf5; Gp2; Il1rn; Kirrel3; Lao1; Lgals3bp; Lipf; Mup20; Npy; Pigr; Pla2g10; Pla2g7; S100b; Spp1; Tg; Timp1; Wnt8a	extracellular region	Component
324	FR247760	PDZ domain binding	0.000296238	Frzb; Grid2; Kirrel3; Lrp2; Pdzk1; Ushbp1	PDZ domain binding	Function
325	FR249432	brush border membrane	0.000341341	Folr1; Lrp2; Pdzk1; Shank2	brush border membrane	Component
326	FR263105	IPAF inflammasome complex	0.000465309	Casp4; Naip2	IPAF inflammasome complex	Component
327	FR263105	amine metabolic process	0.000465309	Cyp1a1; Vcam1	amine metabolic process	Process
328	FR266999	mitochondrial inner membrane	0.00028356	Abcb8; Atp5h; Cox15; Cpt2; Gatm; Idh2; Micu1; Slc25a1; Slc25a5	mitochondrial inner membrane	Component
329	FR269524	fatty acid beta-oxidation using acyl-CoA dehydrogenase	0.000118067	Acad11; Etfdh	fatty acid beta-oxidation using acyl-CoA dehydrogenase	Process
330	FR269524	transport	0.00013597	Abcb8; Atp5h; Chmp4c; Cpt2; Etfdh; Fabp3; Grid2; Kcnk4; Mup20; Pdzk1; Pex14; Pitpnc1; Rab17; Slc25a42; Trpm3	transport	Process
331	FR273612	fatty acid beta-oxidation using acyl-CoA dehydrogenase	0.000431597	Acad11; Etfdh	fatty acid beta-oxidation using acyl-CoA dehydrogenase	Process
332	FR278883	extracellular space	0.000246213	Angptl4; Apln; Cx3cl1; Cxcl1; Dkk2; Frzb; Gdf5; Il1rn; Kng2; Lgals3bp; Lrg1; Npy; Prtn3; S100b; Selp; Tg; Timp1; Vcam1	extracellular space	Component
333	FR278883	extracellular region	0.000307397	1700029I15Rik; Angptl4; Apoc1; Cma1; Cpn2; Cx3cl1; Cxcl1; Defb28; Dkk2; Fgl1; Folr1; Frzb; Galp; Gdf5; Il1rn; Lao1; Lgals3bp; Lipf; Npy; Pla2g10; S100b; Tg; Timp1	extracellular region	Component

334	FR278883	brush border membrane	0.000441707	Folr1; Pdzk1; Shank2; Slc34a3	brush border membrane	Component
335	FR279378	mitochondrial inner membrane	0.000168103	Abcb8; Atp5h; Cox15; Cpt2; Etfdh; Gatm; Idh2; Micu1; Slc25a42; Slc25a5	mitochondrial inner membrane	Component
336	FR279378	transporter activity	0.000257636	Aqp4; Fabp3; Grid2; Mup20; Slc17a9; Slc22a23; Slc25a5	transporter activity	Function
337	FR279905	brush border membrane	0.000399334	Lrp2; Pdzk1; Shank2; Slc34a3	brush border membrane	Component
338	FR283082	mitochondrial inner membrane	0.000364271	Abcb8; Atp5h; Cox15; Cpt2; Etfdh; Gatm; Idh2; Micu1; Slc25a42	mitochondrial inner membrane	Component
339	FR283503	pyroptosis	0.000182463	Casp4; Naip2	pyroptosis	Process
340	FR283503	inflammatory response	0.000349412	Clec7a; Cxcl1; Naip2; Pla2g7; Selp	inflammatory response	Process
341	FR283504	mitochondrial inner membrane	0.000361352	Abcb8; Atp5h; Cox15; Cpt2; Etfdh; Gatm; Idh2; Micu1; Slc25a1; Slc25a42; Slc25a5	mitochondrial inner membrane	Component
342	FR286933	brush border membrane	0.000289647	Folr1; Lrp2; Pdzk1; Shank2	brush border membrane	Component
343	FR287008	brush border membrane	0.000369523	Folr1; Lrp2; Pdzk1; Shank2	brush border membrane	Component
344	FR296035	mitochondrial inner membrane	0.000298388	Abcb8; Atp5h; Cox15; Cpt2; Etfdh; Gatm; Idh2; Micu1; Slc25a5	mitochondrial inner membrane	Component
345	FR301533	brush border membrane	0.000332299	Folr1; Lrp2; Pdzk1; Shank2	brush border membrane	Component
346	FR301567	PDZ domain binding	0.000254135	Grid2; Kirrel3; Lrp2; Pdzk1; Ushbp1	PDZ domain binding	Function
347	FR301567	receptor activity	0.000268312	Cd200r4; Folr1; Grid2; Grik3; Lrp2; Plxna4	receptor activity	Function
348	FR301809	PDZ domain binding	0.0004494	Grid2; Kirrel3; Lrp2; Pdzk1; Ushbp1	PDZ domain binding	Function
349	FR302003	brush border membrane	0.000332299	Folr1; Lrp2; Pdzk1; Shank2	brush border membrane	Component
350	FR302411	apical plasma membrane	0.000212838	Gp2; Lrp2; P2ry1; Pdzk1; Podxl; Rab17; Shank2; Slc34a3; Slc39a4	apical plasma membrane	Component
351	FR302411	PDZ domain binding	0.000462766	Frzb; Grid2; Kirrel3; Lrp2; Pdzk1; Ushbp1	PDZ domain binding	Function
352	FR312018	extracellular vesicular exosome	0.000117906	Chmp4c; Cpn2; Ctsz; Dpp6; Fgl1; Ftl1; Gatm; Il1rn; Lgals3bp; Lrg1; Naip2; Rap2c; Rnf149; Sirpb1a; Slc25a5; Spp1; Timp1; Vcam1	extracellular vesicular exosome	Component
353	FR312018	IPAF inflammasome complex	0.000128753	Casp4; Naip2	IPAF inflammasome complex	Component
354	FR312018	pyroptosis	0.000239418	Casp4; Naip2	pyroptosis	Process
355	FR312018	negative regulation of interleukin-1 beta production	0.000307233	Acp5; Mefv	negative regulation of interleukin-1 beta production	Process

356	FR312018	extracellular space	0.000401884	Ctsz; Frzb; Il1rn; Lgals3bp; Lrg1; Npy; Pla2g7; Selp; Spp1; Timp1; Vcam1	extracellular space	Component
357	FR312820	apical plasma membrane	0.000264107	Gp2; Lrp2; Pdzk1; Podxl; Rab17; Shank2; Slc34a3; Slc39a4	apical plasma membrane	Component
358	FR325017	mitochondrial inner membrane	0.000144794	Abcb8; Atp5h; Cox15; Cpt2; Etfdh; Gatm; Idh2; Micu1; Slc25a1; Slc25a5	mitochondrial inner membrane	Component
359	FR325017	transport	0.000227457	Abcb8; Abcg3; Apoc1; Aqp4; Atp5h; Chmp4c; Cpt2; Etfdh; Fabp3; Folr1; Grid2; Grik3; Kcnk4; Kcnq4; Mcoln3; Micu1; Pdzk1; Pitpnc1; Rab17; Slc10a5; Slc17a9; Slc25a1; Slc25a5; Slc39a4; Slc6a7; Trpm3	transport	Process
360	FR326589	mitochondrial inner membrane	0.000269346	Abcb8; Atp5h; Cox15; Cpt2; Gatm; Idh2; Micu1; Slc25a1; Slc25a5	mitochondrial inner membrane	Component
361	FR330268	apical plasma membrane	0.000443375	Gp2; Lrp2; P2ry1; Pdzk1; Podxl; Rab17; Shank2; Slc39a4	apical plasma membrane	Component
362	FR333763	fatty acid beta-oxidation using acyl-CoA dehydrogenase	0.000332126	Acad11; Etfdh	fatty acid beta-oxidation using acyl-CoA dehydrogenase	Process
363	FR333763	dipeptidyl-peptidase activity	0.00046353	Dpep2; Dpp10	dipeptidyl-peptidase activity	Function
364	FR334236	transport	0.000135974	Abcg3; Apoc1; Aqp4; Chmp4c; Cpt2; Etfdh; Fabp3; Folr1; Grid2; Kcnk4; Kcnq4; Mcoln3; Micu1; Pdzk1; Pex14; Pitpnc1; Rab17; Slc10a5; Slc17a9; Slc22a23; Slc25a42; Slc25a5; Slc34a3; Slc39a4; Slc6a7; Trpm3	transport	Process
365	FR337786	mitochondrial inner membrane	0.000206825	Abcb8; Atp5h; Cox15; Cpt2; Gatm; Idh2; Micu1; Slc25a1; Slc25a5	mitochondrial inner membrane	Component
366	FR341317	extracellular space	0.000233693	Angptl4; Anxa13; Apln; Bpifa1; Ctsz; Cx3cl1; Dkk2; Fabp3; Frzb; Il1rn; Kng2; Lgals3bp; Npy; Pigr; Pla2g7; Prtn3; S100b; Selp; Vcam1; Wnt8a	extracellular space	Component
367	FR341317	apical plasma membrane	0.000496305	Gp2; P2ry1; Pdzk1; Podxl; Rab17; Shank2; Slc34a3; Slc39a4	apical plasma membrane	Component

368	FR347914	mitochondrial inner membrane	0.00042119	Abcb8; Atp5h; Cox15; Cpt2; Gatm; Idh2; Micu1; Slc25a1; Slc25a5	mitochondrial inner membrane	Component
369	FR365932	transport	0.00013217	Abcb8; Abcg3; Atp5h; Chmp4c; Cpt2; Etfdh; Fabp3; Folr1; Grid2; Grik3; Kcnk4; Mcoln3; Micu1; Mup20; Pdzk1; Pitpnc1; Rab17; Slc10a5; Slc17a9; Slc25a1; Slc25a42; Slc25a5; Slc39a4; Slc6a7; Trpm3	transport	Process
370	FR366845	PDZ domain binding	0.000433668	Grid2; Kirrel3; Lrp2; Pdzk1; Ushbp1	PDZ domain binding	Function
371	FR366845	receptor activity	0.000499048	Cd200r4; Folr1; Grid2; Grik3; Lrp2; Plxna4	receptor activity	Function
372	FR374009	PDZ domain binding	0.000465558	Grid2; Kirrel3; Lrp2; Pdzk1; Ushbp1	PDZ domain binding	Function
373	FR375498	brush border membrane	0.000389213	Folr1; Lrp2; Pdzk1; Shank2	brush border membrane	Component
374	FR375933	mitochondrion	0.000169909	Abcb8; Adhfe1; Atp5h; Chchd10; Cox15; Cpt2; Etfdh; Gatm; Hspe1; Idh2; Micu1; Nme4; P2ry1; Slc25a1; Slc25a42; Slc25a5	mitochondrion	Component
375	FR375933	apical plasma membrane	0.000249226	Gp2; Lrp2; P2ry1; Pdzk1; Rab17; Slc39a4	apical plasma membrane	Component
376	FR375933	dipeptidyl-peptidase activity	0.000264062	Dpep2; Dpp10	dipeptidyl-peptidase activity	Function
377	FR375933	phospholipase activity	0.000268559	Abhd15; Nceh1; Pla2g10	phospholipase activity	Function
378	FR375933	transcytosis	0.000450568	Lrp2; Rab17	transcytosis	Process
379	FR380314	fatty acid beta-oxidation using acyl-CoA dehydrogenase	0.000314906	Acad11; Etfdh	fatty acid beta-oxidation using acyl-CoA dehydrogenase	Process
380	FR382833	mitochondrial inner membrane	0.000194494	Abcb8; Atp5h; Cox15; Cpt2; Etfdh; Gatm; Idh2; Micu1; Slc25a1; Slc25a5	mitochondrial inner membrane	Component
381	FR382845	mitochondrial inner membrane	0.000195182	Cox15; Cpt2; Etfdh; Idh2; Prkar2b; Slc25a42	mitochondrial inner membrane	Component
382	FR382845	semaphorin receptor activity	0.00025571	Nrp2; Plxna4	semaphorin receptor activity	Function
383	FR387804	brush border membrane	0.000350557	Folr1; Lrp2; Pdzk1; Shank2	brush border membrane	Component
384	FR388349	IPAF inflammasome complex	0.000139898	Casp4; Naip2	IPAF inflammasome complex	Component
385	FR388349	fatty acid beta-oxidation using acyl-CoA dehydrogenase	0.000139898	Acad11; Etfdh	fatty acid beta-oxidation using acyl-CoA dehydrogenase	Process
386	FR388349	pyroptosis	0.000260098	Casp4; Naip2	pyroptosis	Process
387	FR394282	PDZ domain binding	0.000243938	Grid2; Kirrel3; Lrp2; Pdzk1; Ushbp1	PDZ domain binding	Function

388	FR394282	recycling endosome membrane	0.000306628	Pigr; Rab17; Rap2c	recycling endosome membrane	Component
389	FR396644	IPAF inflammasome complex	0.000392792	Casp4; Naip2	IPAF inflammasome complex	Component
390	FR398266	fatty acid beta-oxidation using acyl-CoA dehydrogenase	0.000132417	Acad11; Etfdh	fatty acid beta-oxidation using acyl-CoA dehydrogenase	Process
391	FR398266	heterophilic cell-cell adhesion	0.00014542	Grid2; Selp; Vcam1	heterophilic cell-cell adhesion	Process
392	FR398266	blood microparticle	0.00025458	Angptl4; Cpn2; Kng2; Lgals3bp	blood microparticle	Component
393	FR398266	semaphorin receptor activity	0.00039417	Nrp2; Plxna4	semaphorin receptor activity	Function
394	FR402142	dipeptidyl-peptidase activity	0.00014155	Dpep2; Dpp10	dipeptidyl-peptidase activity	Function
395	Gomafu	brush border membrane	0.000369523	Folr1; Lrp2; Pdzk1; Shank2	brush border membrane	Component
396	n263541	transport	0.000276235	Abcb8; Abcg3; Aqp4; Atp5h; Chmp4c; Cpt2; Etfdh; Fabp3; Folr1; Grid2; Grik3; Kcnk4; Kcnq4; Mcoln3; Micu1; Pdzk1; Pex14; Pitpnc1; Rab17; Slc10a5; Slc17a9; Slc22a23; Slc39a4; Slc6a7; Trpm3	transport	Process
397	n263575	fatty acid beta-oxidation using acyl-CoA dehydrogenase	0.000458467	Acad11; Etfdh	fatty acid beta-oxidation using acyl-CoA dehydrogenase	Process
398	n263685	apical plasma membrane	0.000270994	Gp2; Lrp2; P2ry1; Pdzk1; Podxl; Rab17; Slc39a4	apical plasma membrane	Component
399	n263685	PDZ domain binding	0.000361146	Grid2; Kirrel3; Lrp2; Pdzk1; Ushbp1	PDZ domain binding	Function
400	n263685	receptor binding	0.000392917	Apln; Gdf5; Kng2; Pex14; S100b; Six3; Tg; Wnt8a	receptor binding	Function
401	n266159	transport	0.000131492	Abcb8; Apoc1; Aqp4; Atp5h; Chmp4c; Cpt2; Etfdh; Fabp3; Grid2; Kcnk4; Kcnq4; Mcoln3; Pdzk1; Pex14; Pitpnc1; Rab17; Slc10a5; Slc25a42; Slc25a5; Slc34a3; Trpm3	transport	Process
402	n266159	regulation of body fluid levels	0.000471669	Apln; Aplnr	regulation of body fluid levels	Process

403	n271825	transport	0.000161014	Abcb8; Abcg3; Atp5h; Chmp4c; Cpt2; Fabp3; Grid2; Grik3; Kcnk4; Kcnq4; Mcoln3; Micu1; Pdzk1; Pitpnc1; Rab17; Slc10a5; Slc17a9; Slc25a1; Slc25a5; Slc39a4; Slc6a7; Trpm3	transport	Process
404	n271825	apical plasma membrane	0.000299072	Gp2; Lrp2; P2ry1; Pdzk1; Podxl; Rab17; Slc39a4	apical plasma membrane	Component
405	n271825	PDZ domain binding	0.000388956	Grid2; Kirrel3; Lrp2; Pdzk1; Ushbp1	PDZ domain binding	Function
406	n272641	mitochondrial inner membrane	0.000160006	Atp5h; Cox15; Cpt2; Etfdh; Gatm; Idh2; Micu1; Prkar2b; Slc25a42; Slc25a5	mitochondrial inner membrane	Component
407	n272641	transporter activity	0.000248313	Aqp4; Fabp3; Grid2; Mup20; Slc17a9; Slc22a23; Slc25a5	transporter activity	Function
408	n273954	transport	0.000118347	Abcb8; Abcg3; Apoc1; Aqp4; Atp5h; Chmp4c; Cpt2; Etfdh; Grid2; Grik3; Kcnk4; Kcnq4; Mcoln3; Micu1; Pdzk1; Pex14; Pitpnc1; Rab17; Slc10a5; Slc17a9; Slc22a23; Slc25a5; Slc39a4; Slc6a7; Trpm3	transport	Process
409	n273954	mitochondrial inner membrane	0.000329973	Abcb8; Atp5h; Cox15; Cpt2; Etfdh; Gatm; Idh2; Micu1; Slc25a5	mitochondrial inner membrane	Component
410	n280804	brush border membrane	0.000369523	Folr1; Lrp2; Pdzk1; Shank2	brush border membrane	Component
411	n280963	IPAF inflammasome complex	0.000132417	Casp4; Naip2	IPAF inflammasome complex	Component
412	n280963	pyroptosis	0.000246218	Casp4; Naip2	pyroptosis	Process
413	n280963	negative regulation of interleukin-1 beta production	0.00031595	Acp5; Mefv	negative regulation of interleukin-1 beta production	Process
414	n280963	immune system process	0.000481291	Cd180; Cd300ld; Clec7a; Mefv; Naip2; Themis2	immune system process	Process
415	n283487	fatty acid beta-oxidation using acyl-CoA dehydrogenase	0.00015547	Acad11; Etfdh	fatty acid beta-oxidation using acyl-CoA dehydrogenase	Process
416	n283487	regulation of body fluid levels	0.000217199	Apln; Aplnr	regulation of body fluid levels	Process
417	n283487	blood microparticle	0.000346157	Angptl4; Cpn2; Kng2; Lgals3bp	blood microparticle	Component
418	n283487	negative regulation of interleukin-1 beta production	0.00037077	Acp5; Aqp4	negative regulation of interleukin-1 beta production	Process
419	n283487	negative regulation of vasoconstriction	0.00037077	Apln; Cx3cl1	negative regulation of vasoconstriction	Process

420	n283793	brush border membrane	0.000297843	Folr1; Lrp2; Pdzk1; Shank2	brush border membrane	Component
421	n289317	PDZ domain binding	0.000418354	Grid2; Kirrel3; Lrp2; Pdzk1; Ushbp1	PDZ domain binding	Function
422	n289317	receptor activity	0.000478696	Cd200r4; Folr1; Grid2; Grik3; Lrp2; Plxna4	receptor activity	Function
423	n289590	fatty acid beta-oxidation using acyl-CoA dehydrogenase	0.00012514	Acad11; Etfdh	fatty acid beta-oxidation using acyl-CoA dehydrogenase	Process
424	n289590	blood microparticle	0.000228372	Angptl4; Cpn2; Kng2; Lgals3bp	blood microparticle	Component
425	n289590	negative regulation of interleukin-1 beta production	0.000298637	Acp5; Aqp4	negative regulation of interleukin-1 beta production	Process
426	n289590	extracellular space	0.000354239	Angptl4; Bpifa1; Ctsz; Fabp3; Frzb; Kng2; Lgals3bp; Pla2g7; Prtn3; S100b; Wnt8a	extracellular space	Component
427	n289590	semaphorin receptor activity	0.000372591	Nrp2; Plxna4	semaphorin receptor activity	Function
428	n290758	mitochondrial inner membrane	0.000118003	Abcb8; Atp5h; Cox15; Cpt2; Etfdh; Gatm; Idh2; Micu1; Slc25a42; Slc25a5	mitochondrial inner membrane	Component
429	n290758	transporter activity	0.000197915	Aqp4; Fabp3; Grid2; Mup20; Slc17a9; Slc22a23; Slc25a5	transporter activity	Function
430	n291764	phospholipase activity	0.000406291	Abhd15; Nceh1; Pla2g10	phospholipase activity	Function
431	n292619	apical plasma membrane	0.000379583	Gp2; Lrp2; P2ry1; Pdzk1; Rab17; Slc34a3; Slc39a4	apical plasma membrane	Component
432	n292619	fatty acid beta-oxidation using acyl-CoA dehydrogenase	0.000431597	Acad11; Etfdh	fatty acid beta-oxidation using acyl-CoA dehydrogenase	Process
433	n292619	recycling endosome membrane	0.00046235	Pigr; Rab17; Rap2c	recycling endosome membrane	Component
434	n294011	brush border membrane	0.000162761	Folr1; Pdzk1; Shank2; Slc34a3	brush border membrane	Component
435	n294011	regulation of body fluid levels	0.000488155	Apln; Aplnr	regulation of body fluid levels	Process
436	n294791	brush border membrane	0.000314733	Folr1; Lrp2; Pdzk1; Shank2	brush border membrane	Component
437	n295927	fatty acid beta-oxidation using acyl-CoA dehydrogenase	0.000104535	Acad11; Etfdh	fatty acid beta-oxidation using acyl-CoA dehydrogenase	Process
438	n295927	semaphorin receptor activity	0.000311448	Nrp2; Plxna4	semaphorin receptor activity	Function
439	n296710	brush border membrane	0.000341341	Folr1; Lrp2; Pdzk1; Shank2	brush border membrane	Component
440	n297428	fatty acid beta-oxidation using acyl-CoA dehydrogenase	0.000276487	Acad11; Etfdh	fatty acid beta-oxidation using acyl-CoA dehydrogenase	Process

441	n297428	extracellular space	0.000315261	Anxa13; Apln; Bpifa1; Ctsz; Fabp3; Frzb; Kng2; Lgals3bp; Npy; Pigr; Pla2g7; Prtn3; S100b; Wnt8a	extracellular space	Component
442	n343177	PDZ domain binding	0.000482151	Grid2; Kirrel3; Lrp2; Pdzk1; Ushbp1	PDZ domain binding	Function
443	n343233	extracellular space	0.000206719	Angptl4; Ctsz; Cxcl1; Dkk2; Frzb; Il1rn; Lgals3bp; Lrg1; Npy; Pla2g7; Selp; Spp1; Tg; Timp1; Vcam1	extracellular space	Component
444	n343233	amine metabolic process	0.000320596	Cyp1a1; Vcam1	amine metabolic process	Process
445	n343233	IPAF inflammasome complex	0.000320596	Casp4; Naip2	IPAF inflammasome complex	Component
446	n343233	dipeptidyl-peptidase activity	0.000447463	Dpep2; Dpp10	dipeptidyl-peptidase activity	Function
447	n414277	mitochondrial inner membrane	0.000281316	Cox15; Cpt2; Etfdh; Idh2; Micu1; Slc25a1; Slc25a5	mitochondrial inner membrane	Component
448	n414277	transcytosis	0.00046108	Rab17; Tg	transcytosis	Process
449	n414371	brush border membrane	0.000350557	Folr1; Lrp2; Pdzk1; Shank2	brush border membrane	Component
450	n415160	neutrophil chemotaxis	0.000115476	Cx3cl1; Cxcl1; Spp1	neutrophil chemotaxis	Process
451	n415160	pyroptosis	0.000159605	Casp4; Naip2	pyroptosis	Process
452	n415160	negative regulation of interleukin-1 beta production	0.000204887	Acp5; Mefv	negative regulation of interleukin-1 beta production	Process
453	n415160	cell adhesion	0.000225519	Cx3cl1; Lgals3bp; Nrp2; Sdc3; Selp; Spp1; Vcam1	cell adhesion	Process
454	n415160	extracellular vesicular exosome	0.000337096	Chmp4c; Ctsz; Fgl1; Ftl1; Il1rn; Lgals3bp; Lrg1; Naip2; Rap2c; Rnf149; Sirpb1a; Slc25a5; Spp1; Timp1; Vcam1	extracellular vesicular exosome	Component
455	n415160	negative regulation of interleukin-12 production	0.000373876	Acp5; Mefv	negative regulation of interleukin-12 production	Process
456	n415160	response to virus	0.000459386	Bcl3; Dhx58; Ifi27l2a	response to virus	Process
457	n415206	IPAF inflammasome complex	0.000221109	Casp4; Naip2	IPAF inflammasome complex	Component
458	n415206	pyroptosis	0.000410654	Casp4; Naip2	pyroptosis	Process
459	n415282	receptor binding	0.000127303	Apln; Galp; Gdf5; Kng2; Pex14; S100b; Six3; Tg; Wnt8a	receptor binding	Function

460	n415323	transport	0.000115589	Abcb8; Aqp4; Atp5h; Chmp4c; Cpt2; Etfdh; Fabp3; Grid2; Kcnk4; Kcnq4; Mcoln3; Mup20; Pdzk1; Pex14; Pitpnc1; Rab17; Slc10a5; Slc25a42; Slc25a5; Slc39a4; Trpm3	transport	Process
461	n415323	fatty acid beta-oxidation using acyl-CoA dehydrogenase	0.000332126	Acad11; Etfdh	fatty acid beta-oxidation using acyl-CoA dehydrogenase	Process
462	n415372	brush border membrane	0.000281614	Folr1; Lrp2; Pdzk1; Shank2	brush border membrane	Component
463	n415399	external side of plasma membrane	0.000127242	Aqp4; Clec7a; Ly6g; Selp; Slamf7; Vcam1	external side of plasma membrane	Component
464	n415399	IPAF inflammasome complex	0.000128753	Casp4; Naip2	IPAF inflammasome complex	Component
465	n415399	pyroptosis	0.000239418	Casp4; Naip2	pyroptosis	Process
466	n415399	extracellular region	0.000304469	Angptl4; Apoc1; Cma1; Cpn2; Cx3cl1; Defb28; Dkk2; Frzb; Galp; Il1rn; Lgals3bp; Npy; Pla2g7; Spp1	extracellular region	Component
467	n415399	negative regulation of interleukin-1 beta production	0.000307233	Acp5; Aqp4	negative regulation of interleukin-1 beta production	Process
468	n416957	apical plasma membrane	0.000329441	Gp2; Lrp2; P2ry1; Pdzk1; Podxl; Rab17; Slc39a4	apical plasma membrane	Component
469	n416957	PDZ domain binding	0.000418354	Grid2; Kirrel3; Lrp2; Pdzk1; Ushbp1	PDZ domain binding	Function
470	n416957	receptor activity	0.000478696	Cd200r4; Folr1; Grid2; Grik3; Lrp2; Plxna4	receptor activity	Function
471	n418309	apical plasma membrane	0.000395125	Gp2; Lrp2; Pdzk1; Podxl; Rab17; Shank2; Slc34a3; Slc39a4	apical plasma membrane	Component
472	n420390	brush border membrane	0.000441707	Folr1; Lrp2; Shank2; Slc34a3	brush border membrane	Component
473	NR_033641.2	PDZ domain binding	0.0004494	Grid2; Kirrel3; Lrp2; Pdzk1; Ushbp1	PDZ domain binding	Function
474	NR_033764.1	transport	0.000305466	Abcb8; Abcg3; Apoc1; Aqp4; Atp5h; Chmp4c; Cpt2; Etfdh; Fabp3; Folr1; Grid2; Grik3; Kcnk4; Kcnq4; Mcoln3; Micu1; Pdzk1; Pex14; Pitpnc1; Rab17; Slc10a5; Slc17a9; Slc25a5; Slc39a4; Slc6a7; Trpm3	transport	Process
475	NR_045776.1	brush border membrane	0.000297843	Folr1; Lrp2; Pdzk1; Shank2	brush border membrane	Component
476	NR_045899.1	apical plasma membrane	0.000416275	Gp2; Lrp2; P2ry1; Pdzk1; Podxl; Rab17; Slc39a4	apical plasma membrane	Component
477	NR_045899.1	PDZ domain binding	0.000499185	Grid2; Kirrel3; Lrp2; Pdzk1; Ushbp1	PDZ domain binding	Function

478	NR_073368.1	transport	0.000124861	Abcb8; Abcg3; Apoc1; Aqp4; Atp5h; Chmp4c; Cpt2; Etfdh; Fabp3; Folr1; Grid2; Grik3; Kcnk4; Kcnq4; Mcoln3; Micu1; Pdzk1; Pex14; Pitpnc1; Rab17; Slc10a5; Slc17a9; Slc22a23; Slc25a5; Slc39a4; Slc6a7; Trpm3	transport	Process
479	NR_076393.1	extracellular space	0.000199818	Angptl4; Anxa13; Apln; Cx3cl1; Dkk2; Frzb; Il1rn; Lgals3bp; Npy; Pigr; Pla2g7; Prtn3; S100b; Selp; Spp1; Vcam1	extracellular space	Component
480	NR_076393.1	inflammatory response	0.000220827	Cd180; Clec7a; Mefv; Naip2; Pla2g7; Selp; Themis2	inflammatory response	Process
481	NR_076393.1	IPAF inflammasome complex	0.000392792	Casp4; Naip2	IPAF inflammasome complex	Component
482	NR_102339.1	IPAF inflammasome complex	0.000255642	Casp4; Naip2	IPAF inflammasome complex	Component
483	NR_102339.1	regulation of body fluid levels	0.000356925	Apln; Aplnr	regulation of body fluid levels	Process
484	NR_102339.1	inflammatory response	0.000450466	Cd180; Clec7a; Mefv; Naip2; Selp; Themis2	inflammatory response	Process
485	NR_102339.1	pyroptosis	0.000474605	Casp4; Naip2	pyroptosis	Process
486	NR_102339.1	immune system process	0.000479736	Cd180; Cd300ld; Clec7a; Dhx58; Mefv; Naip2; Themis2	immune system process	Process
487	uc.31+	apical plasma membrane	0.000245075	Gp2; Lrp2; P2ry1; Pdzk1; Podxl; Rab17; Slc39a4	apical plasma membrane	Component
488	uc.31+	PDZ domain binding	0.000334867	Grid2; Kirrel3; Lrp2; Pdzk1; Ushbp1	PDZ domain binding	Function
489	uc.31+	receptor activity	0.000369777	Cd200r4; Folr1; Grid2; Grik3; Lrp2; Plxna4	receptor activity	Function
490	uc.427+	PDZ domain binding	0.000393459	Frzb; Grid2; Kirrel3; Lrp2; Pdzk1; Ushbp1	PDZ domain binding	Function
491	uc.83+	regulation of body fluid levels	0.000328987	Apln; Aplnr	regulation of body fluid levels	Process
492	XR_104983.1	receptor activity	0.000268312	Cd200r4; Folr1; Grid2; Grik3; Lrp2; Plxna4	receptor activity	Function
493	XR_140871.1	transport	0.000103137	Abcb8; Abcg3; Apoc1; Aqp4; Atp5h; Chmp4c; Cpt2; Etfdh; Fabp3; Folr1; Grid2; Grik3; Kcnk4; Kcnq4; Mcoln3; Micu1; Mup20; Pdzk1; Pex14; Pitpnc1; Rab17; Slc10a5; Slc17a9; Slc25a1; Slc25a5; Slc39a4; Slc6a7; Trpm3	transport	Process
494	XR_140871.1	mitochondrial inner membrane	0.000235026	Abcb8; Atp5h; Cox15; Cpt2; Etfdh; Gatm; Idh2; Micu1; Slc25a1; Slc25a5	mitochondrial inner membrane	Component

495	XR_141342.2	transporter activity	0.000100669	Aqp4; Fabp3; Grid2; Mup20; Slc25a5	transporter activity	Function
496	XR_141342.2	negative regulation of interleukin-1 beta production	0.000234204	Acp5; Aqp4	negative regulation of interleukin-1 beta production	Process
497	XR_141342.2	semaphorin receptor activity	0.000292268	Nrp2; Plxna4	semaphorin receptor activity	Function
498	XR_168932.1	regulation of body fluid levels	0.000155401	Apln; Aplnr	regulation of body fluid levels	Process
499	XR_168932.1	negative regulation of vasoconstriction	0.000265456	Apln; Cx3cl1	negative regulation of vasoconstriction	Process
500	XR_168932.1	negative regulation of Wnt signaling pathway	0.000335493	Dkk2; Frzb; Six3	negative regulation of Wnt signaling pathway	Process

Table-S5 KEGG pathway analysis of top 500 lncRNA-correlated mRNAs

No	groups	enrichmentTerm	p.value	enrichmentIDs	pathway_desc
1	chr1_134167181_134181386_R	Tryptophan metabolism	0.000287955	Cyp1a1; Lao1; Tph1	Tryptophan metabolism
2	chr11_33414450_33450750_F	Tryptophan metabolism	0.000424673	Cyp1a1; Lao1; Tph1	Tryptophan metabolism
3	n415160	TNF signaling pathway	0.000826565	Bcl3; Cx3cl1; Cxcl1; Vcam1	TNF signaling pathway
4	FR168758	Tryptophan metabolism	0.000889281	Cyp1a1; Lao1; Tph1	Tryptophan metabolism
5	chr13_34872696_34906407_R	Tryptophan metabolism	0.000889281	Cyp1a1; Lao1; Tph1	Tryptophan metabolism
6	chr16_44720170_44723911_R	Tryptophan metabolism	0.000889281	Cyp1a1; Lao1; Tph1	Tryptophan metabolism
7	chr9_4260788_4261404_F	Tryptophan metabolism	0.000889281	Cyp1a1; Lao1; Tph1	Tryptophan metabolism
8	FR065159	Tryptophan metabolism	0.000889281	Cyp1a1; Lao1; Tph1	Tryptophan metabolism
9	chr8_108153471_108159672_F	Tryptophan metabolism	0.000974112	Cyp1a1; Lao1; Tph1	Tryptophan metabolism
10	FR396644	Tryptophan metabolism	0.000974112	Cyp1a1; Lao1; Tph1	Tryptophan metabolism
11	FR114000	Tryptophan metabolism	0.000974112	Cyp1a1; Lao1; Tph1	Tryptophan metabolism
12	chr14_73729977_73751877_R	Tryptophan metabolism	0.000974112	Cyp1a1; Lao1; Tph1	Tryptophan metabolism
13	n343177	Tryptophan metabolism	0.001063907	Cyp1a1; Lao1; Tph1	Tryptophan metabolism
14	FR134333	Tryptophan metabolism	0.001063907	Cyp1a1; Lao1; Tph1	Tryptophan metabolism
15	XR_104983.1	Tryptophan metabolism	0.001063907	Cyp1a1; Lao1; Tph1	Tryptophan metabolism
16	chr13_74642700_74701900_F	Tryptophan metabolism	0.001158777	Cyp1a1; Lao1; Tph1	Tryptophan metabolism
17	FR032503	Tryptophan metabolism	0.001158777	Cyp1a1; Lao1; Tph1	Tryptophan metabolism
18	FR090859	Tryptophan metabolism	0.001158777	Cyp1a1; Lao1; Tph1	Tryptophan metabolism
19	FR215148	Tryptophan metabolism	0.001158777	Cyp1a1; Lao1; Tph1	Tryptophan metabolism
20	chr2_35828611_35838083_F	Tryptophan metabolism	0.001158777	Cyp1a1; Lao1; Tph1	Tryptophan metabolism
21	n415206	NOD-like receptor signaling pathway	0.001179041	Cxcl1; Mefv; Naip2	NOD-like receptor signaling pathway
22	XR_141936.2	Tryptophan metabolism	0.001258828	Cyp1a1; Lao1; Tph1	Tryptophan metabolism
23	n289317	Tryptophan metabolism	0.001258828	Cyp1a1; Lao1; Tph1	Tryptophan metabolism
24	chr1_183142656_183163206_R	Tryptophan metabolism	0.001258828	Cyp1a1; Lao1; Tph1	Tryptophan metabolism
25	chr8_126396600_126407275_R	Tryptophan metabolism	0.001258828	Cyp1a1; Lao1; Tph1	Tryptophan metabolism
26	FR289473	Tryptophan metabolism	0.001258828	Cyp1a1; Lao1; Tph1	Tryptophan metabolism
27	FR366845	Tryptophan metabolism	0.001258828	Cyp1a1; Lao1; Tph1	Tryptophan metabolism
28	FR052210	Tryptophan metabolism	0.001258828	Cyp1a1; Lao1; Tph1	Tryptophan metabolism
29	FR279620	Tryptophan metabolism	0.001258828	Cyp1a1; Lao1; Tph1	Tryptophan metabolism
30	FR149067	Tryptophan metabolism	0.001258828	Cyp1a1; Lao1; Tph1	Tryptophan metabolism
31	chr15_78403377_78412545_F	Tryptophan metabolism	0.001258828	Cyp1a1; Lao1; Tph1	Tryptophan metabolism
32	n416957	Tryptophan metabolism	0.001364168	Cyp1a1; Lao1; Tph1	Tryptophan metabolism
33	chr18_70400082_70404236_R	Tryptophan metabolism	0.001364168	Cyp1a1; Lao1; Tph1	Tryptophan metabolism
34	ENSMUST00000139056	Tryptophan metabolism	0.001364168	Cyp1a1; Lao1; Tph1	Tryptophan metabolism

35	NR_033641.2	Tryptophan metabolism	0.001364168	Cyp1a1; Lao1; Tph1	Tryptophan metabolism
36	FR186619	Tryptophan metabolism	0.001364168	Cyp1a1; Lao1; Tph1	Tryptophan metabolism
37	FR202182	Tryptophan metabolism	0.001364168	Cyp1a1; Lao1; Tph1	Tryptophan metabolism
38	n415160	NOD-like receptor signaling pathway	0.001445613	Cxcl1; Mefv; Naip2	NOD-like receptor signaling pathway
39	FR115704	Tryptophan metabolism	0.001474899	Cyp1a1; Lao1; Tph1	Tryptophan metabolism
40	NR_045776.1	Tryptophan metabolism	0.001474899	Cyp1a1; Lao1; Tph1	Tryptophan metabolism
41	FR268824	Tryptophan metabolism	0.001474899	Cyp1a1; Lao1; Tph1	Tryptophan metabolism
42	FR100940	Tryptophan metabolism	0.001474899	Cyp1a1; Lao1; Tph1	Tryptophan metabolism
43	FR301809	Tryptophan metabolism	0.001474899	Cyp1a1; Lao1; Tph1	Tryptophan metabolism
44	FR003993	Tryptophan metabolism	0.001474899	Cyp1a1; Lao1; Tph1	Tryptophan metabolism
45	FR168142	Tryptophan metabolism	0.001474899	Cyp1a1; Lao1; Tph1	Tryptophan metabolism
46	FR066795	Tryptophan metabolism	0.001474899	Cyp1a1; Lao1; Tph1	Tryptophan metabolism
47	chr13_1078220 42_107823753_ F	Tryptophan metabolism	0.001474899	Cyp1a1; Lao1; Tph1	Tryptophan metabolism
48	chr12_5527169 1_55276492_R	Tryptophan metabolism	0.001474899	Cyp1a1; Lao1; Tph1	Tryptophan metabolism
49	FR382845	PPAR signaling pathway	0.001571141	Angptl4; Cpt2; Fabp3	PPAR signaling pathway
50	ENSMUST000 00134627	Tryptophan metabolism	0.001591123	Cyp1a1; Lao1; Tph1	Tryptophan metabolism
51	n263685	Tryptophan metabolism	0.001591123	Cyp1a1; Lao1; Tph1	Tryptophan metabolism
52	NR_045899.1	Tryptophan metabolism	0.001591123	Cyp1a1; Lao1; Tph1	Tryptophan metabolism
53	FR099681	Tryptophan metabolism	0.001591123	Cyp1a1; Lao1; Tph1	Tryptophan metabolism
54	FR097477	Tryptophan metabolism	0.001591123	Cyp1a1; Lao1; Tph1	Tryptophan metabolism
55	FR074898	Tryptophan metabolism	0.001591123	Cyp1a1; Lao1; Tph1	Tryptophan metabolism
56	chr7_13437239 2_134372943_ R	Tryptophan metabolism	0.001591123	Cyp1a1; Lao1; Tph1	Tryptophan metabolism
57	FR374009	Tryptophan metabolism	0.001591123	Cyp1a1; Lao1; Tph1	Tryptophan metabolism
58	chr1_75075500 _75102200_R	Tryptophan metabolism	0.001591123	Cyp1a1; Lao1; Tph1	Tryptophan metabolism
59	FR150621	Tryptophan metabolism	0.001591123	Cyp1a1; Lao1; Tph1	Tryptophan metabolism
60	FR042390	Tryptophan metabolism	0.001712938	Cyp1a1; Lao1; Tph1	Tryptophan metabolism
61	FR155040	Tryptophan metabolism	0.001712938	Cyp1a1; Lao1; Tph1	Tryptophan metabolism
62	FR285140	Tryptophan metabolism	0.001712938	Cyp1a1; Lao1; Tph1	Tryptophan metabolism
63	chr15_9659733 0_96663683_F	Tryptophan metabolism	0.001712938	Cyp1a1; Lao1; Tph1	Tryptophan metabolism
64	FR254927	Tryptophan metabolism	0.001712938	Cyp1a1; Lao1; Tph1	Tryptophan metabolism
65	chr12_1687341 0_16873872_F	Tryptophan metabolism	0.001712938	Cyp1a1; Lao1; Tph1	Tryptophan metabolism
66	FR171433	Tryptophan metabolism	0.001712938	Cyp1a1; Lao1; Tph1	Tryptophan metabolism
67	FR117744	Tryptophan metabolism	0.001712938	Cyp1a1; Lao1; Tph1	Tryptophan metabolism
68	chr7_91558475 _91733625_R	Tryptophan metabolism	0.001712938	Cyp1a1; Lao1; Tph1	Tryptophan metabolism
69	FR120704	Tryptophan metabolism	0.001712938	Cyp1a1; Lao1; Tph1	Tryptophan metabolism
70	n415282	Tryptophan metabolism	0.001712938	Cyp1a1; Lao1; Tph1	Tryptophan metabolism
71	FR090778	NOD-like receptor signaling pathway	0.001747235	Cxcl1; Mefv; Naip2	NOD-like receptor signaling pathway
72	FR162485	Tryptophan metabolism	0.001840441	Cyp1a1; Lao1; Tph1	Tryptophan metabolism
73	chr13_9828115 8_98285757_F	Tryptophan metabolism	0.001840441	Cyp1a1; Lao1; Tph1	Tryptophan metabolism
74	FR078628	Tryptophan metabolism	0.001840441	Cyp1a1; Lao1; Tph1	Tryptophan metabolism
75	FR054476	Tryptophan metabolism	0.001840441	Cyp1a1; Lao1; Tph1	Tryptophan metabolism
76	FR019981	Tryptophan metabolism	0.001840441	Cyp1a1; Lao1; Tph1	Tryptophan metabolism

77	FR385633	Tryptophan metabolism	0.001840441	Cyp1a1; Lao1; Tph1	Tryptophan metabolism
78	FR139436	Tryptophan metabolism	0.001840441	Cyp1a1; Lao1; Tph1	Tryptophan metabolism
79	FR233225	Tryptophan metabolism	0.001840441	Cyp1a1; Lao1; Tph1	Tryptophan metabolism
80	n280804	Tryptophan metabolism	0.001840441	Cyp1a1; Lao1; Tph1	Tryptophan metabolism
81	FR286933	Tryptophan metabolism	0.001840441	Cyp1a1; Lao1; Tph1	Tryptophan metabolism
82	FR396644	NOD-like receptor signaling pathway	0.001911686	Cxcl1; Mefv; Naip2	NOD-like receptor signaling pathway
83	FR007873	Tryptophan metabolism	0.001973726	Cyp1a1; Lao1; Tph1	Tryptophan metabolism
84	chr3_86827945_86886695_R	Tryptophan metabolism	0.001973726	Cyp1a1; Lao1; Tph1	Tryptophan metabolism
85	chr17_29374758_29387733_R	Tryptophan metabolism	0.001973726	Cyp1a1; Lao1; Tph1	Tryptophan metabolism
86	n265955	Tryptophan metabolism	0.001973726	Cyp1a1; Lao1; Tph1	Tryptophan metabolism
87	FR117799	Tryptophan metabolism	0.001973726	Cyp1a1; Lao1; Tph1	Tryptophan metabolism
88	n265736	Tryptophan metabolism	0.001973726	Cyp1a1; Lao1; Tph1	Tryptophan metabolism
89	FR046015	Thyroid hormone synthesis	0.001979627	Lrp2; Tg; Tshr	Thyroid hormone synthesis
90	FR028133	Thyroid hormone synthesis	0.001979627	Lrp2; Tg; Tshr	Thyroid hormone synthesis
91	n292619	Cell adhesion molecules (CAMs)	0.002098733	Icos; Nrxn2; Sdc3; Selp; Vcam1	Cell adhesion molecules (CAMs)
92	FR302003	Tryptophan metabolism	0.002112886	Cyp1a1; Lao1; Tph1	Tryptophan metabolism
93	FR249432	Tryptophan metabolism	0.002112886	Cyp1a1; Lao1; Tph1	Tryptophan metabolism
94	Gomafu	Tryptophan metabolism	0.002112886	Cyp1a1; Lao1; Tph1	Tryptophan metabolism
95	FR301533	Tryptophan metabolism	0.002112886	Cyp1a1; Lao1; Tph1	Tryptophan metabolism
96	ENSMUST00000132130	Tryptophan metabolism	0.002112886	Cyp1a1; Lao1; Tph1	Tryptophan metabolism
97	FR298560	Tryptophan metabolism	0.002112886	Cyp1a1; Lao1; Tph1	Tryptophan metabolism
98	chr2_157838320_157853495_F	Tryptophan metabolism	0.002112886	Cyp1a1; Lao1; Tph1	Tryptophan metabolism
99	FR266999	Tryptophan metabolism	0.002112886	Cyp1a1; Lao1; Tph1	Tryptophan metabolism
100	FR326589	Tryptophan metabolism	0.002112886	Cyp1a1; Lao1; Tph1	Tryptophan metabolism
101	FR199374	Tryptophan metabolism	0.002112886	Cyp1a1; Lao1; Tph1	Tryptophan metabolism
102	chr3_35782698_35789932_R	Tryptophan metabolism	0.002112886	Cyp1a1; Lao1; Tph1	Tryptophan metabolism
103	FR337786	Tryptophan metabolism	0.002112886	Cyp1a1; Lao1; Tph1	Tryptophan metabolism
104	chr12_110003268_110030953_F	Tryptophan metabolism	0.002258011	Cyp1a1; Lao1; Tph1	Tryptophan metabolism
105	FR019091	Tryptophan metabolism	0.002258011	Cyp1a1; Lao1; Tph1	Tryptophan metabolism
106	chr19_23068397_23190256_R	Tryptophan metabolism	0.002258011	Cyp1a1; Lao1; Tph1	Tryptophan metabolism
107	chr11_6796679_6797670_F	Tryptophan metabolism	0.002258011	Cyp1a1; Lao1; Tph1	Tryptophan metabolism
108	chr5_31883451_31884082_F	Tryptophan metabolism	0.002258011	Cyp1a1; Lao1; Tph1	Tryptophan metabolism
109	n273521	Tryptophan metabolism	0.002258011	Cyp1a1; Lao1; Tph1	Tryptophan metabolism
110	chrX_18734106_18744367_R	Tryptophan metabolism	0.002258011	Cyp1a1; Lao1; Tph1	Tryptophan metabolism
111	chr1_138475514_138476054_R	Tryptophan metabolism	0.002258011	Cyp1a1; Lao1; Tph1	Tryptophan metabolism
112	n296710	Tryptophan metabolism	0.002258011	Cyp1a1; Lao1; Tph1	Tryptophan metabolism
113	chr13_66381200_66410200_F	Tryptophan metabolism	0.002258011	Cyp1a1; Lao1; Tph1	Tryptophan metabolism

114	FR185165	Tryptophan metabolism	0.002258011	Cyp1a1; Lao1; Tph1	Tryptophan metabolism
115	chr15_32167516_32174417_R	Tryptophan metabolism	0.002258011	Cyp1a1; Lao1; Tph1	Tryptophan metabolism
116	chr1_162965001_162966376_F	Tryptophan metabolism	0.002258011	Cyp1a1; Lao1; Tph1	Tryptophan metabolism
117	FR389545	Tryptophan metabolism	0.002258011	Cyp1a1; Lao1; Tph1	Tryptophan metabolism
118	FR375498	Tryptophan metabolism	0.002409188	Cyp1a1; Lao1; Tph1	Tryptophan metabolism
119	FR358023	Tryptophan metabolism	0.002409188	Cyp1a1; Lao1; Tph1	Tryptophan metabolism
120	chr4_88745771_88776323_F	Tryptophan metabolism	0.002409188	Cyp1a1; Lao1; Tph1	Tryptophan metabolism
121	FR215734	Tryptophan metabolism	0.002409188	Cyp1a1; Lao1; Tph1	Tryptophan metabolism
122	chr8_119732348_119749198_R	Tryptophan metabolism	0.002409188	Cyp1a1; Lao1; Tph1	Tryptophan metabolism
123	FR391590	Tryptophan metabolism	0.002409188	Cyp1a1; Lao1; Tph1	Tryptophan metabolism
124	n414371	Tryptophan metabolism	0.002409188	Cyp1a1; Lao1; Tph1	Tryptophan metabolism
125	FR057053	Tryptophan metabolism	0.002409188	Cyp1a1; Lao1; Tph1	Tryptophan metabolism
126	chr10_8606683_8665783_R	Tryptophan metabolism	0.002409188	Cyp1a1; Lao1; Tph1	Tryptophan metabolism
127	FR136228	Tryptophan metabolism	0.002409188	Cyp1a1; Lao1; Tph1	Tryptophan metabolism
128	FR347914	Tryptophan metabolism	0.002409188	Cyp1a1; Lao1; Tph1	Tryptophan metabolism
129	ENSMUST00000149707	NOD-like receptor signaling pathway	0.002461855	Cxcl1; Mefv; Naip2	NOD-like receptor signaling pathway
130	ENSMUST00000150216	Tryptophan metabolism	0.002566503	Cyp1a1; Lao1; Tph1	Tryptophan metabolism
131	chr1_196445304_196445818_R	Tryptophan metabolism	0.002566503	Cyp1a1; Lao1; Tph1	Tryptophan metabolism
132	FR028809	Tryptophan metabolism	0.002566503	Cyp1a1; Lao1; Tph1	Tryptophan metabolism
133	chr12_89519675_89533975_F	Tryptophan metabolism	0.002566503	Cyp1a1; Lao1; Tph1	Tryptophan metabolism
134	ENSMUST00000181563	Tryptophan metabolism	0.002566503	Cyp1a1; Lao1; Tph1	Tryptophan metabolism
135	FR087516	Tryptophan metabolism	0.002566503	Cyp1a1; Lao1; Tph1	Tryptophan metabolism
136	FR087735	Tryptophan metabolism	0.002566503	Cyp1a1; Lao1; Tph1	Tryptophan metabolism
137	chr1_138570107_138632310_R	Tryptophan metabolism	0.002566503	Cyp1a1; Lao1; Tph1	Tryptophan metabolism
138	XR_140433.1	Tryptophan metabolism	0.002566503	Cyp1a1; Lao1; Tph1	Tryptophan metabolism
139	FR177295	Tryptophan metabolism	0.002566503	Cyp1a1; Lao1; Tph1	Tryptophan metabolism
140	n290305	Tryptophan metabolism	0.002566503	Cyp1a1; Lao1; Tph1	Tryptophan metabolism
141	chr6_37630192_37682692_R	Tryptophan metabolism	0.002566503	Cyp1a1; Lao1; Tph1	Tryptophan metabolism
142	n289590	PPAR signaling pathway	0.002572143	Angptl4; Cpt2; Fabp3	PPAR signaling pathway
143	FR326589	Metabolic pathways	0.002601753	Atp5h; Cox15; Cyp1a1; Fut2; Gatm; Ggt1; Idh2; Impdh1; Lao1; Lipf; Nme4; Phgdh; Pigx; Pla2g10; Tph1	Metabolic pathways
144	FR298560	Metabolic pathways	0.002601753	Atp5h; Cox15; Cyp1a1; Fut2; Gatm; Ggt1; Idh2; Impdh1; Lao1; Lipf; Nme4; Phgdh; Pigx; Pla2g10; Tph1	Metabolic pathways

145	FR090859	Metabolic pathways	0.002625848	Atp5h; Cox15; Cyp1a1; Fut2; Gatm; Ggt1; Idh2; Lao1; Lipf; Nme4; Pigx; Pla2g10; Tph1	Metabolic pathways
146	FR374009	Metabolic pathways	0.002639623	Atp5h; Cox15; Cyp1a1; Fut2; Gatm; Ggt1; Idh2; Lao1; Lipf; Nme4; Phgdh; Pigx; Pla2g10; Tph1	Metabolic pathways
147	FR097477	Metabolic pathways	0.002639623	Atp5h; Cox15; Cyp1a1; Fut2; Gatm; Ggt1; Idh2; Lao1; Lipf; Nme4; Phgdh; Pigx; Pla2g10; Tph1	Metabolic pathways
148	n283487	Renin-angiotensin system	0.002695293	Cma1; Nln	Renin-angiotensin system
149	chr7_82902248_82955248_F	Tryptophan metabolism	0.00273004	Cyp1a1; Lao1; Tph1	Tryptophan metabolism
150	n412060	Tryptophan metabolism	0.00273004	Cyp1a1; Lao1; Tph1	Tryptophan metabolism
151	FR101946	Tryptophan metabolism	0.00273004	Cyp1a1; Lao1; Tph1	Tryptophan metabolism
152	chr3_21964899_21974885_F	Tryptophan metabolism	0.00273004	Cyp1a1; Lao1; Tph1	Tryptophan metabolism
153	FR125080	Tryptophan metabolism	0.00273004	Cyp1a1; Lao1; Tph1	Tryptophan metabolism
154	chr18_67435075_67447150_F	Tryptophan metabolism	0.00273004	Cyp1a1; Lao1; Tph1	Tryptophan metabolism
155	FR325017	Tryptophan metabolism	0.00273004	Cyp1a1; Lao1; Tph1	Tryptophan metabolism
156	ENSMUST00000163396	Tryptophan metabolism	0.00273004	Cyp1a1; Lao1; Tph1	Tryptophan metabolism
157	chr9_67414463_67483185_R	Tryptophan metabolism	0.00273004	Cyp1a1; Lao1; Tph1	Tryptophan metabolism
158	n296070	Tryptophan metabolism	0.00273004	Cyp1a1; Lao1; Tph1	Tryptophan metabolism
159	n413982	Tryptophan metabolism	0.00273004	Cyp1a1; Lao1; Tph1	Tryptophan metabolism
160	FR238565	Tryptophan metabolism	0.00273004	Cyp1a1; Lao1; Tph1	Tryptophan metabolism
161	chr4_123572090_123581365_F	TNF signaling pathway	0.002747311	Bcl3; Cx3cl1; Vcam1	TNF signaling pathway
162	FR398266	Cell adhesion molecules (CAMs)	0.0028949	Nrxn2; Sdc3; Selp; Vcam1	Cell adhesion molecules (CAMs)
163	FR100205	Cell adhesion molecules (CAMs)	0.0028949	Nrxn2; Sdc3; Selp; Vcam1	Cell adhesion molecules (CAMs)
164	chr18_5813291_5813708_R	Tryptophan metabolism	0.002899882	Cyp1a1; Lao1; Tph1	Tryptophan metabolism
165	chr8_26345571_26346095_R	Tryptophan metabolism	0.002899882	Cyp1a1; Lao1; Tph1	Tryptophan metabolism
166	chr9_14480441_14483362_F	Tryptophan metabolism	0.002899882	Cyp1a1; Lao1; Tph1	Tryptophan metabolism
167	chr2_151992806_152025456_F	Tryptophan metabolism	0.002899882	Cyp1a1; Lao1; Tph1	Tryptophan metabolism
168	n263541	Tryptophan metabolism	0.002899882	Cyp1a1; Lao1; Tph1	Tryptophan metabolism
169	chr10_18036490_18059965_F	Tryptophan metabolism	0.002899882	Cyp1a1; Lao1; Tph1	Tryptophan metabolism
170	chr13_98513868_98517366_R	Tryptophan metabolism	0.002899882	Cyp1a1; Lao1; Tph1	Tryptophan metabolism

171	chr3_40439525_40509975_R	Tryptophan metabolism	0.002899882	Cyp1a1; Lao1; Tph1	Tryptophan metabolism
172	chr12_111287772_111333890_F	Tryptophan metabolism	0.002899882	Cyp1a1; Lao1; Tph1	Tryptophan metabolism
173	n419646	Tryptophan metabolism	0.002899882	Cyp1a1; Lao1; Tph1	Tryptophan metabolism
174	chr1_69531191_69570566_F	Thyroid hormone synthesis	0.002961545	Lrp2; Tg; Tshr	Thyroid hormone synthesis
175	FR237950	Tryptophan metabolism	0.003076106	Cyp1a1; Lao1; Tph1	Tryptophan metabolism
176	chr7_80762708_80763274_R	Tryptophan metabolism	0.003076106	Cyp1a1; Lao1; Tph1	Tryptophan metabolism
177	XR_141927.1	Tryptophan metabolism	0.003076106	Cyp1a1; Lao1; Tph1	Tryptophan metabolism
178	uc.411+	Tryptophan metabolism	0.003076106	Cyp1a1; Lao1; Tph1	Tryptophan metabolism
179	chr15_38205154_38206196_F	Tryptophan metabolism	0.003076106	Cyp1a1; Lao1; Tph1	Tryptophan metabolism
180	FR395103	Tryptophan metabolism	0.003076106	Cyp1a1; Lao1; Tph1	Tryptophan metabolism
181	chr4_116790373_116797823_F	Tryptophan metabolism	0.003076106	Cyp1a1; Lao1; Tph1	Tryptophan metabolism
182	ENSMUST00000157205	Tryptophan metabolism	0.003076106	Cyp1a1; Lao1; Tph1	Tryptophan metabolism
183	FR061730	Tryptophan metabolism	0.003076106	Cyp1a1; Lao1; Tph1	Tryptophan metabolism
184	chr8_48476637_48492537_F	Tryptophan metabolism	0.003076106	Cyp1a1; Lao1; Tph1	Tryptophan metabolism
185	chr8_96983590_96994419_F	TNF signaling pathway	0.003226253	Bcl3; Cx3cl1; Cxcl1; Vcam1	TNF signaling pathway
186	NR_033764.1	Tryptophan metabolism	0.003258792	Cyp1a1; Lao1; Tph1	Tryptophan metabolism
187	FR283931	Tryptophan metabolism	0.003258792	Cyp1a1; Lao1; Tph1	Tryptophan metabolism
188	chr9_121845920_121858120_F	Tryptophan metabolism	0.003258792	Cyp1a1; Lao1; Tph1	Tryptophan metabolism
189	FR065962	Tryptophan metabolism	0.003258792	Cyp1a1; Lao1; Tph1	Tryptophan metabolism
190	FR187810	Tryptophan metabolism	0.003258792	Cyp1a1; Lao1; Tph1	Tryptophan metabolism
191	chr9_56646768_56649068_R	Tryptophan metabolism	0.003258792	Cyp1a1; Lao1; Tph1	Tryptophan metabolism
192	chr15_100908323_100908645_F	Tryptophan metabolism	0.003258792	Cyp1a1; Lao1; Tph1	Tryptophan metabolism
193	FR312820	Tryptophan metabolism	0.003258792	Cyp1a1; Lao1; Tph1	Tryptophan metabolism
194	chr13_15947521_15999246_R	Tryptophan metabolism	0.003258792	Cyp1a1; Lao1; Tph1	Tryptophan metabolism
195	FR017535	Tryptophan metabolism	0.003258792	Cyp1a1; Lao1; Tph1	Tryptophan metabolism
196	FR030947	Tryptophan metabolism	0.003258792	Cyp1a1; Lao1; Tph1	Tryptophan metabolism
197	n272641	Tryptophan metabolism	0.003258792	Cyp1a1; Lao1; Tph1	Tryptophan metabolism
198	chr14_55317199_55317670_R	Tryptophan metabolism	0.003448015	Cyp1a1; Lao1; Tph1	Tryptophan metabolism
199	chr9_58301467_58323783_F	Tryptophan metabolism	0.003448015	Cyp1a1; Lao1; Tph1	Tryptophan metabolism
200	chr2_72818943_72826893_R	Tryptophan metabolism	0.003448015	Cyp1a1; Lao1; Tph1	Tryptophan metabolism
201	FR382833	Tryptophan metabolism	0.003448015	Cyp1a1; Lao1; Tph1	Tryptophan metabolism
202	FR279378	Tryptophan metabolism	0.003448015	Cyp1a1; Lao1; Tph1	Tryptophan metabolism
203	n290758	Tryptophan metabolism	0.003448015	Cyp1a1; Lao1; Tph1	Tryptophan metabolism
204	chr7_133402766_133415516_R	Tryptophan metabolism	0.003448015	Cyp1a1; Lao1; Tph1	Tryptophan metabolism
205	chr11_120079199_120130099_R	Tryptophan metabolism	0.003448015	Cyp1a1; Lao1; Tph1	Tryptophan metabolism

206	chr14_33923944_34003394_F	Tryptophan metabolism	0.003448015	Cyp1a1; Lao1; Tph1	Tryptophan metabolism
207	chr10_39666719_39679959_R	Tryptophan metabolism	0.003448015	Cyp1a1; Lao1; Tph1	Tryptophan metabolism
208	chr7_26212709_26230775_F	Tryptophan metabolism	0.003448015	Cyp1a1; Lao1; Tph1	Tryptophan metabolism
209	FR016598	Tryptophan metabolism	0.003448015	Cyp1a1; Lao1; Tph1	Tryptophan metabolism
210	n418283	Tryptophan metabolism	0.003448015	Cyp1a1; Lao1; Tph1	Tryptophan metabolism
211	FR171433	Metabolic pathways	0.003455252	Atp5h; Cox15; Cyp1a1; Fut2; Gatm; Ggt1; Idh2; Lao1; Lipf; Nme4; Phgdh; Pigx; Pla2g10; Tph1	Metabolic pathways
212	chr12_16873410_16873872_F	Metabolic pathways	0.003455252	Atp5h; Cox15; Cyp1a1; Fut2; Gatm; Ggt1; Idh2; Lao1; Lipf; Nme4; Phgdh; Pigx; Pla2g10; Tph1	Metabolic pathways
213	chr15_96597330_96663683_F	Metabolic pathways	0.003455252	Atp5h; Cox15; Cyp1a1; Fut2; Gatm; Ggt1; Idh2; Lao1; Lipf; Nme4; Phgdh; Pigx; Pla2g10; Tph1	Metabolic pathways
214	FR052210	Metabolic pathways	0.00349966	Atp5h; Cox15; Cyp1a1; Fut2; Gatm; Ggt1; Idh2; Lao1; Lipf; Nme4; Pigx; Pla2g10; Tph1	Metabolic pathways
215	chr1_183142656_183163206_R	Metabolic pathways	0.00349966	Atp5h; Cox15; Cyp1a1; Fut2; Gatm; Ggt1; Idh2; Lao1; Lipf; Nme4; Pigx; Pla2g10; Tph1	Metabolic pathways
216	FR366845	Metabolic pathways	0.00349966	Atp5h; Cox15; Cyp1a1; Fut2; Gatm; Ggt1; Idh2; Lao1; Lipf; Nme4; Pigx; Pla2g10; Tph1	Metabolic pathways
217	FR279620	Metabolic pathways	0.00349966	Atp5h; Cox15; Cyp1a1; Fut2; Gatm; Ggt1; Idh2; Lao1; Lipf; Nme4; Pigx; Pla2g10; Tph1	Metabolic pathways
218	XR_141936.2	Metabolic pathways	0.00349966	Atp5h; Cox15; Cyp1a1; Fut2; Gatm; Ggt1; Idh2; Lao1; Lipf; Nme4; Pigx; Pla2g10; Tph1	Metabolic pathways
219	n289317	Metabolic pathways	0.00349966	Atp5h; Cox15; Cyp1a1; Fut2; Gatm; Ggt1; Idh2; Lao1; Lipf; Nme4; Pigx; Pla2g10; Tph1	Metabolic pathways
220	FR194887	TNF signaling pathway	0.003510169	Bcl3; Cx3cl1; Cxcl1; Vcam1	TNF signaling pathway
221	FR398266	PPAR signaling pathway	0.003532455	Angptl4; Cpt2; Fabp3	PPAR signaling pathway
222	FR100205	PPAR signaling pathway	0.003532455	Angptl4; Cpt2; Fabp3	PPAR signaling pathway
223	FR040835	Thyroid hormone synthesis	0.003546787	Lrp2; Tg; Tshr	Thyroid hormone synthesis

224	chr6_86425510_86450160_F	TNF signaling pathway	0.003634747	Bcl3; Cxcl1; Vcam1	TNF signaling pathway
225	XR_140871.1	Tryptophan metabolism	0.003643849	Cyp1a1; Lao1; Tph1	Tryptophan metabolism
226	chr18_69923410_69965360_R	Tryptophan metabolism	0.003643849	Cyp1a1; Lao1; Tph1	Tryptophan metabolism
227	NR_073368.1	Tryptophan metabolism	0.003846365	Cyp1a1; Lao1; Tph1	Tryptophan metabolism
228	chr13_34693404_34717987_F	Tryptophan metabolism	0.003846365	Cyp1a1; Lao1; Tph1	Tryptophan metabolism
229	chr2_157236070_157249445_R	Tryptophan metabolism	0.003846365	Cyp1a1; Lao1; Tph1	Tryptophan metabolism
230	chr9_66976750_67014625_F	Tryptophan metabolism	0.003846365	Cyp1a1; Lao1; Tph1	Tryptophan metabolism
231	FR134333	Thyroid hormone synthesis	0.003864027	Lrp2; Tg; Tshr	Thyroid hormone synthesis
232	n291764	Thyroid hormone synthesis	0.003864027	Lrp2; Tg; Tshr	Thyroid hormone synthesis
233	n415399	Phagosome	0.003915636	Cd209a; Cd209f; Clec7a; Ncf4	Phagosome
234	FR180118	Phagosome	0.003915636	Cd209a; Cd209f; Clec7a; Ncf4	Phagosome
235	FR047247	Tryptophan metabolism	0.004055633	Cyp1a1; Lao1; Tph1	Tryptophan metabolism
236	chr6_47959825_47960502_R	Tryptophan metabolism	0.004055633	Cyp1a1; Lao1; Tph1	Tryptophan metabolism
237	chr13_76326228_76332632_F	Tryptophan metabolism	0.004055633	Cyp1a1; Lao1; Tph1	Tryptophan metabolism
238	ENSMUST00000141575	Tryptophan metabolism	0.004055633	Cyp1a1; Lao1; Tph1	Tryptophan metabolism
239	chr8_96983590_96994419_F	NOD-like receptor signaling pathway	0.00409879	Cxcl1; Mefv; Naip2	NOD-like receptor signaling pathway
240	chr9_27148989_27155714_R	Cell adhesion molecules (CAMs)	0.004175518	Icos; Nrxn2; Sdc3; Selp	Cell adhesion molecules (CAMs)
241	chr7_31709550_31723025_F	Tryptophan metabolism	0.004271722	Cyp1a1; Lao1; Tph1	Tryptophan metabolism
242	FR204953	Tryptophan metabolism	0.004271722	Cyp1a1; Lao1; Tph1	Tryptophan metabolism
243	n418309	Tryptophan metabolism	0.004271722	Cyp1a1; Lao1; Tph1	Tryptophan metabolism
244	chr15_60732209_60732822_R	Tryptophan metabolism	0.004271722	Cyp1a1; Lao1; Tph1	Tryptophan metabolism
245	chr4_123572090_123581365_F	Cytokine-cytokine receptor interaction	0.004315273	Csf2rb; Cx3cl1; Gdf5; Il20ra	Cytokine-cytokine receptor interaction
246	FR194887	NOD-like receptor signaling pathway	0.004375265	Cxcl1; Mefv; Naip2	NOD-like receptor signaling pathway
247	FR078628	Metabolic pathways	0.004465267	Atp5h; Cox15; Cyp1a1; Fut2; Gatm; Ggt1; Idh2; Impdh1; Lao1; Lipf; Nme4; Pigx; Pla2g10; Tph1	Metabolic pathways
248	XR_105914.2	Tryptophan metabolism	0.004494698	Cyp1a1; Lao1; Tph1	Tryptophan metabolism
249	chr6_115468025_115489250_R	Tryptophan metabolism	0.004494698	Cyp1a1; Lao1; Tph1	Tryptophan metabolism
250	FR330268	Tryptophan metabolism	0.004494698	Cyp1a1; Lao1; Tph1	Tryptophan metabolism
251	FR160504	Tryptophan metabolism	0.004494698	Cyp1a1; Lao1; Tph1	Tryptophan metabolism
252	n290544	Tryptophan metabolism	0.004494698	Cyp1a1; Lao1; Tph1	Tryptophan metabolism

253	chr19_33049479_33054553_R	Renin-angiotensin system	0.004527167	Cma1; Nln	Renin-angiotensin system
254	FR186619	Metabolic pathways	0.00459519	Atp5h; Cox15; Cyp1a1; Fut2; Gatm; Ggt1; Idh2; Lao1; Lipf; Nme4; Pigx; Pla2g10; Tph1	Metabolic pathways
255	NR_033641.2	Metabolic pathways	0.00459519	Atp5h; Cox15; Cyp1a1; Fut2; Gatm; Ggt1; Idh2; Lao1; Lipf; Nme4; Pigx; Pla2g10; Tph1	Metabolic pathways
256	FR114000	Metabolic pathways	0.004645024	Cox15; Cyp1a1; Fut2; Gatm; Ggt1; Idh2; Lao1; Lipf; Nme4; Pigx; Pla2g10; Tph1	Metabolic pathways
257	ENSMUST00000125250	Tryptophan metabolism	0.004724625	Cyp1a1; Lao1; Tph1	Tryptophan metabolism
258	FR278883	Tryptophan metabolism	0.004724625	Cyp1a1; Lao1; Tph1	Tryptophan metabolism
259	chr5_35893908_35896613_R	Tryptophan metabolism	0.004724625	Cyp1a1; Lao1; Tph1	Tryptophan metabolism
260	n343233	NOD-like receptor signaling pathway	0.004961566	Cxcl1; Mefv; Naip2	NOD-like receptor signaling pathway
261	FR055065	Tryptophan metabolism	0.004961566	Cyp1a1; Lao1; Tph1	Tryptophan metabolism
262	chr1_183783306_183834531_F	Tryptophan metabolism	0.004961566	Cyp1a1; Lao1; Tph1	Tryptophan metabolism
263	FR247760	Tryptophan metabolism	0.004961566	Cyp1a1; Lao1; Tph1	Tryptophan metabolism
264	FR251112	Tryptophan metabolism	0.004961566	Cyp1a1; Lao1; Tph1	Tryptophan metabolism
265	chr8_48476637_48492537_F	TNF signaling pathway	0.005186589	Bcl3; Cx3cl1; Cxcl1; Vcam1	TNF signaling pathway
266	FR082156	Tryptophan metabolism	0.005205581	Cyp1a1; Lao1; Tph1	Tryptophan metabolism
267	chr14_25923295_25923893_F	Tryptophan metabolism	0.005205581	Cyp1a1; Lao1; Tph1	Tryptophan metabolism
268	FR351980	Tryptophan metabolism	0.005205581	Cyp1a1; Lao1; Tph1	Tryptophan metabolism
269	chr10_62262341_62269593_F	Thyroid hormone synthesis	0.005303312	Lrp2; Tg; Tshr	Thyroid hormone synthesis
270	n290305	Metabolic pathways	0.005404269	Atp5h; Cox15; Cyp1a1; Fut2; Gatm; Ggt1; Idh2; Impdh1; Lao1; Lipf; Nme4; Phgdh; Pigx; Pla2g10; Tph1	Metabolic pathways
271	FR028809	Metabolic pathways	0.005404269	Atp5h; Cox15; Cyp1a1; Fut2; Gatm; Ggt1; Idh2; Impdh1; Lao1; Lipf; Nme4; Phgdh; Pigx; Pla2g10; Tph1	Metabolic pathways
272	chr3_21964899_21974524_F	PPAR signaling pathway	0.005567243	Angptl4; Cpt2; Fabp3	PPAR signaling pathway
273	FR007873	Metabolic pathways	0.005701445	Atp5h; Cox15; Cyp1a1; Fut2; Gatm; Ggt1; Idh2; Lao1; Lipf; Nme4; Phgdh; Pigx; Pla2g10; Tph1	Metabolic pathways

274	n265955	Metabolic pathways	0.005701445	Atp5h; Cox15; Cyp1a1; Fut2; Gatm; Ggt1; Idh2; Lao1; Lipf; Nme4; Phgdh; Pigx; Pla2g10; Tph1	Metabolic pathways
275	n265736	Metabolic pathways	0.005701445	Atp5h; Cox15; Cyp1a1; Fut2; Gatm; Ggt1; Idh2; Lao1; Lipf; Nme4; Phgdh; Pigx; Pla2g10; Tph1	Metabolic pathways
276	chr17_29374758_29387733_R	Metabolic pathways	0.005701445	Atp5h; Cox15; Cyp1a1; Fut2; Gatm; Ggt1; Idh2; Lao1; Lipf; Nme4; Phgdh; Pigx; Pla2g10; Tph1	Metabolic pathways
277	FR333763	Bile secretion	0.005706931	Aqp4; Nceh1; Sctr	Bile secretion
278	FR087925	Thyroid hormone synthesis	0.005706931	Lrp2; Tg; Tshr	Thyroid hormone synthesis
279	chr2_116796887_116797441_F	Thyroid hormone synthesis	0.005706931	Lrp2; Tg; Tshr	Thyroid hormone synthesis
280	FR099681	Thyroid hormone synthesis	0.005706931	Lrp2; Tg; Tshr	Thyroid hormone synthesis
281	FR086793	Thyroid hormone synthesis	0.005706931	Lrp2; Tg; Tshr	Thyroid hormone synthesis
282	uc.427+	Tryptophan metabolism	0.005715068	Cyp1a1; Lao1; Tph1	Tryptophan metabolism
283	FR167447	Renin-angiotensin system	0.00573467	Cma1; Nln	Renin-angiotensin system
284	chr8_48476637_48492537_F	NOD-like receptor signaling pathway	0.005926231	Cxcl1; Mefv; Naip2	NOD-like receptor signaling pathway
285	FR301809	Metabolic pathways	0.005950349	Atp5h; Cox15; Cyp1a1; Fut2; Gatm; Ggt1; Idh2; Lao1; Lipf; Nme4; Pigx; Pla2g10; Tph1	Metabolic pathways
286	FR168142	Metabolic pathways	0.005950349	Atp5h; Cox15; Cyp1a1; Fut2; Gatm; Ggt1; Idh2; Lao1; Lipf; Nme4; Pigx; Pla2g10; Tph1	Metabolic pathways
287	chr13_107822042_107823753_F	Metabolic pathways	0.005950349	Atp5h; Cox15; Cyp1a1; Fut2; Gatm; Ggt1; Idh2; Lao1; Lipf; Nme4; Pigx; Pla2g10; Tph1	Metabolic pathways
288	FR268824	Metabolic pathways	0.005950349	Atp5h; Cox15; Cyp1a1; Fut2; Gatm; Ggt1; Idh2; Lao1; Lipf; Nme4; Pigx; Pla2g10; Tph1	Metabolic pathways
289	chr8_41475940_41476460_F	Metabolic pathways	0.005950349	Atp5h; Cox15; Cyp1a1; Fut2; Gatm; Ggt1; Idh2; Lipf; Nme4; Phgdh; Pigx; Pla2g10; Tph1	Metabolic pathways
290	FR087537	Tryptophan metabolism	0.005980654	Cyp1a1; Lao1; Tph1	Tryptophan metabolism
291	FR155648	PPAR signaling pathway	0.006042699	Angptl4; Cpt2; Fabp3	PPAR signaling pathway
292	chr11_120041124_120052299_F	Thyroid hormone synthesis	0.006128517	Lrp2; Tg; Tshr	Thyroid hormone synthesis

293	chr6_86425510_86450160_F	Cytokine-cytokine receptor interaction	0.006172435	Csf2rb; Cxcl1; Gdf5; Il20ra	Cytokine-cytokine receptor interaction
294	chr17_64063362_64066067_R	Tryptophan metabolism	0.006253539	Cyp1a1; Lao1; Tph1	Tryptophan metabolism
295	FR263105	NOD-like receptor signaling pathway	0.006270969	Cxcl1; Mefv; Naip2	NOD-like receptor signaling pathway
296	chr14_21621875_21638625_R	Tryptophan metabolism	0.006533777	Cyp1a1; Lao1; Tph1	Tryptophan metabolism
297	chrX_165792277_165807952_R	Tryptophan metabolism	0.006533777	Cyp1a1; Lao1; Tph1	Tryptophan metabolism
298	FR064953	Bile secretion	0.006568273	Aqp4; Nceh1; Sctr	Bile secretion
299	FR019981	Thyroid hormone synthesis	0.006568273	Lrp2; Tg; Tshr	Thyroid hormone synthesis
300	FR395572	Thyroid hormone synthesis	0.006568273	Lrp2; Tg; Tshr	Thyroid hormone synthesis
301	FR375933	Neuroactive ligand-receptor interaction	0.006637251	Grid2; Grik3; P2ry1; Sctr; Tshr	Neuroactive ligand-receptor interaction
302	chr4_123572090_123581365_F	Malaria	0.006795397	Selp; Vcam1	Malaria
303	FR003529	Tryptophan metabolism	0.006821418	Cyp1a1; Lao1; Tph1	Tryptophan metabolism
304	FR219193	Tryptophan metabolism	0.006821418	Cyp1a1; Lao1; Tph1	Tryptophan metabolism
305	ENSMUST00000125309	Tryptophan metabolism	0.00711651	Cyp1a1; Lao1; Tph1	Tryptophan metabolism
306	ENSMUST00000130677	Tryptophan metabolism	0.00711651	Cyp1a1; Lao1; Tph1	Tryptophan metabolism
307	n295927	Lysosome	0.007175044	Acp5; Cd68; Ctsz	Lysosome
308	n280963	Lysosome	0.007175044	Acp5; Cd68; Ctsz	Lysosome
309	FR337786	Metabolic pathways	0.007197849	Atp5h; Cox15; Cyp1a1; Fut2; Gatm; Ggt1; Idh2; Impdh1; Lao1; Lipf; Nme4; Pigx; Pla2g10; Tph1	Metabolic pathways
310	Gomafu	Metabolic pathways	0.007197849	Atp5h; Cox15; Cyp1a1; Fut2; Gatm; Ggt1; Idh2; Lao1; Lipf; Nme4; Phgdh; Pigx; Pla2g10; Tph1	Metabolic pathways
311	FR199374	Metabolic pathways	0.007197849	Atp5h; Cox15; Cyp1a1; Fut2; Gatm; Ggt1; Idh2; Lao1; Lipf; Nme4; Phgdh; Pigx; Pla2g10; Tph1	Metabolic pathways
312	FR302003	Metabolic pathways	0.007197849	Atp5h; Cox15; Cyp1a1; Fut2; Gatm; Ggt1; Idh2; Lao1; Lipf; Nme4; Phgdh; Pigx; Pla2g10; Tph1	Metabolic pathways
313	FR266999	Metabolic pathways	0.007197849	Atp5h; Cox15; Cyp1a1; Fut2; Gatm; Ggt1; Idh2; Lao1; Lipf; Nme4; Phgdh; Pigx; Pla2g10; Tph1	Metabolic pathways

314	FR249432	Metabolic pathways	0.007197849	Atp5h; Cox15; Cyp1a1; Fut2; Gatm; Ggt1; Idh2; Lao1; Lipf; Nme4; Phgdh; Pigx; Pla2g10; Tph1	Metabolic pathways
315	FR071800	Ether lipid metabolism	0.007500921	Pla2g10; Pla2g7	Ether lipid metabolism
316	chr9_27155685_27156100_F	Ether lipid metabolism	0.007500921	Pla2g10; Pla2g7	Ether lipid metabolism
317	chr6_129151512_129208737_R	Thyroid hormone synthesis	0.007503069	Lrp2; Tg; Tshr	Thyroid hormone synthesis
318	FR341317	Cell adhesion molecules (CAMs)	0.007533614	Icos; Nrxn2; Sdc3; Selp; Vcam1	Cell adhesion molecules (CAMs)
319	n291764	Neuroactive ligand-receptor interaction	0.007552673	Grid2; Grik3; P2ry1; Sctr; Tshr	Neuroactive ligand-receptor interaction
320	ENSMUST00000134627	Metabolic pathways	0.007605712	Atp5h; Cox15; Cyp1a1; Fut2; Gatm; Ggt1; Idh2; Lao1; Lipf; Nme4; Pigx; Pla2g10; Tph1	Metabolic pathways
321	FR208702	Metabolic pathways	0.007605712	Atp5h; Cox15; Cyp1a1; Fut2; Gatm; Ggt1; Idh2; Lipf; Nme4; Phgdh; Pigx; Pla2g10; Tph1	Metabolic pathways
322	chr1_75075500_75102200_R	Metabolic pathways	0.007605712	Atp5h; Cox15; Cyp1a1; Fut2; Gatm; Ggt1; Idh2; Lao1; Lipf; Nme4; Pigx; Pla2g10; Tph1	Metabolic pathways
323	chr13_98038568_98068079_F	Metabolic pathways	0.007605712	Atp5h; Cox15; Cyp1a1; Fut2; Gatm; Ggt1; Idh2; Lipf; Nme4; Phgdh; Pigx; Pla2g10; Tph1	Metabolic pathways
324	NR_045899.1	Metabolic pathways	0.007605712	Cox15; Cyp1a1; Fut2; Gatm; Ggt1; Idh2; Impdh1; Lao1; Lipf; Nme4; Pigx; Pla2g10; Tph1	Metabolic pathways
325	n280963	Osteoclast differentiation	0.007668009	Acp5; Ncf4; Sirpb1a	Osteoclast differentiation
326	chr2_71576218_71603818_R	Tryptophan metabolism	0.007729237	Cyp1a1; Lao1; Tph1	Tryptophan metabolism
327	FR055102	Tryptophan metabolism	0.007729237	Cyp1a1; Lao1; Tph1	Tryptophan metabolism
328	NR_102339.1	Phagosome	0.007768222	Cd209a; Cd209f; Clec7a; Ncf4	Phagosome
329	chr2_35828611_35838083_F	Metabolic pathways	0.007944732	Cox15; Cyp1a1; Fut2; Gatm; Ggt1; Idh2; Lao1; Lipf; Nme4; Pigx; Pla2g10; Tph1	Metabolic pathways
330	FR215148	Metabolic pathways	0.007944732	Cox15; Cyp1a1; Fut2; Gatm; Ggt1; Idh2; Lao1; Lipf; Nme4; Pigx; Pla2g10; Tph1	Metabolic pathways

331	FR032503	Metabolic pathways	0.007944732	Cox15; Cyp1a1; Fut2; Gatm; Ggt1; Idh2; Lao1; Lipf; Nme4; Pigx; Pla2g10; Tph1	Metabolic pathways
332	ENSMUST00000174110	Bile secretion	0.007998466	Aqp4; Nceh1; Sctr	Bile secretion
333	FR010306	Thyroid hormone synthesis	0.007998466	Lrp2; Tg; Tshr	Thyroid hormone synthesis
334	n292619	Bile secretion	0.007998466	Aqp4; Nceh1; Sctr	Bile secretion
335	n415399	TNF signaling pathway	0.008019399	Bcl3; Cx3cl1; Vcam1	TNF signaling pathway
336	FR180118	TNF signaling pathway	0.008019399	Bcl3; Cx3cl1; Vcam1	TNF signaling pathway
337	FR000826	TNF signaling pathway	0.008019399	Bcl3; Cx3cl1; Vcam1	TNF signaling pathway
338	FR323263	Cell adhesion molecules (CAMs)	0.008083092	Icos; Nrxn2; Sdc3; Selp; Vcam1	Cell adhesion molecules (CAMs)
339	FR204953	NOD-like receptor signaling pathway	0.008173144	Cxcl1; Mefv; Naip2	NOD-like receptor signaling pathway
340	chr6_86425510_86450160_F	Malaria	0.008196253	Selp; Vcam1	Malaria
341	n263541	Metabolic pathways	0.00835905	Atp5h; Cox15; Cyp1a1; Fut2; Gatm; Ggt1; Idh2; Impdh1; Lao1; Lipf; Nme4; Phgdh; Pigx; Pla2g10; Tph1	Metabolic pathways
342	chr12_111287772_111333890_F	Metabolic pathways	0.00835905	Atp5h; Cox15; Cyp1a1; Fut2; Gatm; Ggt1; Idh2; Impdh1; Lao1; Lipf; Nme4; Phgdh; Pigx; Pla2g10; Tph1	Metabolic pathways
343	FR358023	Thyroid hormone synthesis	0.008512754	Lrp2; Tg; Tshr	Thyroid hormone synthesis
344	FR392828	Thyroid hormone synthesis	0.008512754	Lrp2; Tg; Tshr	Thyroid hormone synthesis
345	FR391590	Thyroid hormone synthesis	0.008512754	Lrp2; Tg; Tshr	Thyroid hormone synthesis
346	FR060087	Bile secretion	0.008512754	Aqp4; Nceh1; Sctr	Bile secretion
347	FR333763	Cell adhesion molecules (CAMs)	0.008532116	Nrxn2; Sdc3; Selp; Vcam1	Cell adhesion molecules (CAMs)
348	FR029539	Phagosome	0.008587395	Cd209a; Cd209f; Clec7a; Ncf4	Phagosome
349	ENSMUST00000149707	Phagosome	0.008587395	Cd209a; Cd209f; Clec7a; Ncf4	Phagosome
350	FR160504	NOD-like receptor signaling pathway	0.008589923	Cxcl1; Mefv; Naip2	NOD-like receptor signaling pathway
351	FR330268	NOD-like receptor signaling pathway	0.008589923	Cxcl1; Mefv; Naip2	NOD-like receptor signaling pathway
352	n290544	NOD-like receptor signaling pathway	0.008589923	Cxcl1; Mefv; Naip2	NOD-like receptor signaling pathway
353	XR_105914.2	NOD-like receptor signaling pathway	0.008589923	Cxcl1; Mefv; Naip2	NOD-like receptor signaling pathway
354	FR269524	Intestinal immune network for IgA production	0.008771691	Icos; Pigr	Intestinal immune network for IgA production
355	FR269524	Ether lipid metabolism	0.008771691	Pla2g10; Pla2g7	Ether lipid metabolism

356	FR283503	TNF signaling pathway	0.008818603	Bcl3; Cxcl1; Vcam1	TNF signaling pathway
357	n283487	TNF signaling pathway	0.008818603	Bcl3; Cx3cl1; Vcam1	TNF signaling pathway
358	FR278883	TNF signaling pathway	0.008860512	Bcl3; Cx3cl1; Cxcl1; Vcam1	TNF signaling pathway
359	uc.83+	Tryptophan metabolism	0.008948342	Lao1; Tph1	Tryptophan metabolism
360	n273954	Metabolic pathways	0.008990521	Atp5h; Cox15; Fut2; Gatm; Ggt1; Idh2; Impdh1; Lao1; Lipf; Nme4; Phgdh; Pigx; Pla2g10; Tph1	Metabolic pathways
361	FR019091	Metabolic pathways	0.008990521	Atp5h; Cox15; Cyp1a1; Fut2; Gatm; Ggt1; Idh2; Lao1; Lipf; Nme4; Phgdh; Pigx; Pla2g10; Tph1	Metabolic pathways
362	n296710	Metabolic pathways	0.008990521	Atp5h; Cox15; Cyp1a1; Fut2; Gatm; Ggt1; Idh2; Lao1; Lipf; Nme4; Phgdh; Pigx; Pla2g10; Tph1	Metabolic pathways
363	chr1_162965001_162966376_F	Metabolic pathways	0.008990521	Atp5h; Cox15; Cyp1a1; Fut2; Gatm; Ggt1; Idh2; Lao1; Lipf; Nme4; Phgdh; Pigx; Pla2g10; Tph1	Metabolic pathways
364	FR185165	Metabolic pathways	0.008990521	Atp5h; Cox15; Cyp1a1; Fut2; Gatm; Ggt1; Idh2; Lao1; Lipf; Nme4; Phgdh; Pigx; Pla2g10; Tph1	Metabolic pathways
365	chr12_110003268_110030953_F	Metabolic pathways	0.008990521	Atp5h; Cox15; Cyp1a1; Fut2; Gatm; Ggt1; Idh2; Lao1; Lipf; Nme4; Phgdh; Pigx; Pla2g10; Tph1	Metabolic pathways
366	chr1_138473514_138476054_R	Metabolic pathways	0.008990521	Atp5h; Cox15; Cyp1a1; Fut2; Gatm; Ggt1; Idh2; Lao1; Lipf; Nme4; Phgdh; Pigx; Pla2g10; Tph1	Metabolic pathways
367	chr5_31883451_31884082_F	Metabolic pathways	0.008990521	Atp5h; Cox15; Cyp1a1; Fut2; Gatm; Ggt1; Idh2; Lao1; Lipf; Nme4; Phgdh; Pigx; Pla2g10; Tph1	Metabolic pathways
368	FR278883	NOD-like receptor signaling pathway	0.009019012	Cxcl1; Mefv; Naip2	NOD-like receptor signaling pathway
369	XR_140433.1	Thyroid hormone synthesis	0.009046086	Lrp2; Tg; Tshr	Thyroid hormone synthesis
370	ENSMUST00000181563	Thyroid hormone synthesis	0.009046086	Lrp2; Tg; Tshr	Thyroid hormone synthesis
371	FR365932	Thyroid hormone synthesis	0.009046086	Lrp2; Tg; Tshr	Thyroid hormone synthesis

372	FR085857	Ether lipid metabolism	0.009440719	Pla2g10; Pla2g7	Ether lipid metabolism
373	FR278883	Renin-angiotensin system	0.009480729	Cma1; Nln	Renin-angiotensin system
374	FR238565	Thyroid hormone synthesis	0.009598609	Lrp2; Tg; Tshr	Thyroid hormone synthesis
375	chr7_82902248_82955248_F	Thyroid hormone synthesis	0.009598609	Lrp2; Tg; Tshr	Thyroid hormone synthesis
376	chr3_21964899_21974885_F	Thyroid hormone synthesis	0.009598609	Lrp2; Tg; Tshr	Thyroid hormone synthesis
377	FR294279	Metabolic pathways	0.009604081	Atp5h; Cox15; Cyp1a1; Fut2; Gatm; Ggt1; Idh2; Lipf; Nme4; Phgdh; Pigx; Pla2g10; Tph1	Metabolic pathways
378	n294791	Metabolic pathways	0.009604081	Atp5h; Cox15; Cyp1a1; Fut2; Gatm; Ggt1; Idh2; Lipf; Nme4; Phgdh; Pigx; Pla2g10; Tph1	Metabolic pathways
379	chr7_91558475_91733625_R	Metabolic pathways	0.009604081	Atp5h; Cox15; Cyp1a1; Fut2; Gatm; Ggt1; Idh2; Impdh1; Lao1; Lipf; Nme4; Pla2g10; Tph1	Metabolic pathways
380	FR106089	TNF signaling pathway	0.009662888	Bcl3; Cxcl1; Vcam1	TNF signaling pathway
381	FR388349	TNF signaling pathway	0.009662888	Bcl3; Cx3cl1; Vcam1	TNF signaling pathway
382	n415206	Glycine, serine and threonine metabolism	0.009671092	Gatm; Phgdh	Glycine, serine and threonine metabolism
383	FR057875	Glycine, serine and threonine metabolism	0.009671092	Gatm; Phgdh	Glycine, serine and threonine metabolism
384	FR007551	Tryptophan metabolism	0.009750852	Cyp1a1; Lao1; Tph1	Tryptophan metabolism
385	FR054246	NOD-like receptor signaling pathway	0.009914424	Cxcl1; Mefv; Naip2	NOD-like receptor signaling pathway
386	FR384764	Renin-angiotensin system	0.010132224	Cma1; Nln	Renin-angiotensin system
387	FR149067	Metabolic pathways	0.010167268	Cox15; Cyp1a1; Fut2; Gatm; Ggt1; Idh2; Lao1; Lipf; Nme4; Pigx; Pla2g10; Tph1	Metabolic pathways
388	chr8_126396600_126407275_R	Metabolic pathways	0.010167268	Cox15; Cyp1a1; Fut2; Gatm; Ggt1; Idh2; Lao1; Lipf; Nme4; Pigx; Pla2g10; Tph1	Metabolic pathways
389	chr15_78403377_78412545_F	Metabolic pathways	0.010167268	Cox15; Cyp1a1; Fut2; Gatm; Ggt1; Idh2; Lao1; Lipf; Nme4; Pigx; Pla2g10; Tph1	Metabolic pathways
390	chr10_18036490_18059965_F	Thyroid hormone synthesis	0.01017046	Lrp2; Tg; Tshr	Thyroid hormone synthesis
391	chr4_116790373_116797823_F	Metabolic pathways	0.010253936	Atp5h; Cox15; Cyp1a1; Fut2; Gatm; Ggt1; Idh2; Impdh1; Lao1; Lipf; Nme4; Phgdh; Pigx; Pla2g10; Tph1	Metabolic pathways

392	ENSMUST00000157205	Metabolic pathways	0.010253936	Atp5h; Cox15; Cyp1a1; Fut2; Gatm; Ggt1; Idh2; Impdh1; Lao1; Lipf; Nme4; Phgdh; Pigx; Pla2g10; Tph1	Metabolic pathways
393	chr7_80762708_80763274_R	Metabolic pathways	0.010253936	Atp5h; Cox15; Cyp1a1; Fut2; Gatm; Ggt1; Idh2; Impdh1; Lao1; Lipf; Nme4; Phgdh; Pigx; Pla2g10; Tph1	Metabolic pathways
394	FR061730	Metabolic pathways	0.010253936	Atp5h; Cox15; Cyp1a1; Fut2; Gatm; Ggt1; Idh2; Impdh1; Lao1; Lipf; Nme4; Phgdh; Pigx; Pla2g10; Tph1	Metabolic pathways
395	uc.411+	Metabolic pathways	0.010253936	Atp5h; Cox15; Cyp1a1; Fut2; Gatm; Ggt1; Idh2; Impdh1; Lao1; Lipf; Nme4; Phgdh; Pigx; Pla2g10; Tph1	Metabolic pathways
396	chr10_116814196_116920696_R	Glycine, serine and threonine metabolism	0.010352518	Gatm; Phgdh	Glycine, serine and threonine metabolism
397	FR323263	NOD-like receptor signaling pathway	0.010380889	Cxcl1; Mefv; Naip2	NOD-like receptor signaling pathway
398	n280963	Malaria	0.010518244	Selp; Vcam1	Malaria
399	FR090778	TNF signaling pathway	0.010552744	Bcl3; Cxcl1; Vcam1	TNF signaling pathway
400	chr8_48476637_48492537_R	TNF signaling pathway	0.010552744	Bcl3; Cx3cl1; Vcam1	TNF signaling pathway
401	FR302411	Cell adhesion molecules (CAMs)	0.010559807	Icos; Nrxn2; Sdc3; Selp; Vcam1	Cell adhesion molecules (CAMs)
402	chr5_110182844_110228345_R	Cell adhesion molecules (CAMs)	0.010559807	Icos; Nrxn2; Sdc3; Selp; Vcam1	Cell adhesion molecules (CAMs)
403	chr9_27155685_27156100_F	Neuroactive ligand-receptor interaction	0.010666207	Grid2; P2ry1; Sctr; Tshr	Neuroactive ligand-receptor interaction
404	FR203009	Thyroid hormone synthesis	0.010761766	Lrp2; Tg; Tshr	Thyroid hormone synthesis
405	FR203009	Bile secretion	0.010761766	Aqp4; Nceh1; Sctr	Bile secretion
406	FR142459	Ether lipid metabolism	0.010844801	Pla2g10; Pla2g7	Ether lipid metabolism
407	chr10_24269165_24276690_F	Tryptophan metabolism	0.010866744	Cyp1a1; Lao1; Tph1	Tryptophan metabolism
408	FR180118	Lysosome	0.010881252	Acp5; Cd68; Ctsz	Lysosome
409	FR000826	Lysosome	0.010881252	Acp5; Cd68; Ctsz	Lysosome
410	FR312018	Lysosome	0.010881252	Acp5; Cd68; Ctsz	Lysosome
411	FR398266	Lysosome	0.010881252	Acp5; Cd68; Ctsz	Lysosome
412	chr2_132722530_132726802_F	Glycine, serine and threonine metabolism	0.011054674	Gatm; Phgdh	Glycine, serine and threonine metabolism
413	chr4_88745771_88776323_F	Metabolic pathways	0.011117121	Atp5h; Cox15; Cyp1a1; Fut2; Gatm; Ggt1; Idh2; Lao1; Lipf; Nme4; Phgdh; Pigx; Pla2g10; Tph1	Metabolic pathways

414	chr10_8606683_8665783_R	Metabolic pathways	0.011117121	Atp5h; Cox15; Cyp1a1; Fut2; Gatm; Ggt1; Idh2; Lao1; Lipf; Nme4; Phgdh; Pigx; Pla2g10; Tph1	Metabolic pathways
415	FR057053	Metabolic pathways	0.011117121	Atp5h; Cox15; Cyp1a1; Fut2; Gatm; Ggt1; Idh2; Lao1; Lipf; Nme4; Phgdh; Pigx; Pla2g10; Tph1	Metabolic pathways
416	FR375498	Metabolic pathways	0.011117121	Atp5h; Cox15; Cyp1a1; Fut2; Gatm; Ggt1; Idh2; Lao1; Lipf; Nme4; Phgdh; Pigx; Pla2g10; Tph1	Metabolic pathways
417	FR215734	Metabolic pathways	0.011117121	Atp5h; Cox15; Cyp1a1; Fut2; Gatm; Ggt1; Idh2; Impdh1; Lao1; Lipf; Nme4; Pigx; Pla2g10; Tph1	Metabolic pathways
418	FR347914	Metabolic pathways	0.011117121	Atp5h; Cox15; Cyp1a1; Fut2; Gatm; Ggt1; Idh2; Impdh1; Lao1; Lipf; Nme4; Pigx; Pla2g10; Tph1	Metabolic pathways
419	n414371	Metabolic pathways	0.011117121	Atp5h; Cox15; Cyp1a1; Fut2; Gatm; Ggt1; Idh2; Lao1; Lipf; Nme4; Phgdh; Pigx; Pla2g10; Tph1	Metabolic pathways
420	chr8_119732348_119749198_R	Metabolic pathways	0.011117121	Atp5h; Cox15; Cyp1a1; Fut2; Gatm; Ggt1; Idh2; Lao1; Lipf; Nme4; Phgdh; Pigx; Pla2g10; Tph1	Metabolic pathways
421	FR136228	Metabolic pathways	0.011117121	Atp5h; Cox15; Cyp1a1; Fut2; Gatm; Ggt1; Idh2; Impdh1; Lao1; Lipf; Nme4; Pigx; Pla2g10; Tph1	Metabolic pathways
422	FR296035	Metabolic pathways	0.011117121	Atp5h; Cox15; Fut2; Gatm; Ggt1; Idh2; Impdh1; Lao1; Lipf; Nme4; Phgdh; Pigx; Pla2g10; Tph1	Metabolic pathways
423	FR187810	Thyroid hormone synthesis	0.011372647	Lrp2; Tg; Tshr	Thyroid hormone synthesis
424	FR263105	Thyroid hormone synthesis	0.011372647	Lrp2; Tg; Tshr	Thyroid hormone synthesis
425	chr19_33049479_33054553_R	Phagosome	0.011377529	Cd209a; Cd209f; Clec7a; Ncf4	Phagosome
426	FR167447	PPAR signaling pathway	0.011414681	Angptl4; Cpt2; Fabp3	PPAR signaling pathway
427	FR273612	PPAR signaling pathway	0.011414681	Angptl4; Cpt2; Fabp3	PPAR signaling pathway

428	FR180118	Osteoclast differentiation	0.011615554	Acp5; Ncf4; Sirpb1a	Osteoclast differentiation
429	n415399	Osteoclast differentiation	0.011615554	Acp5; Ncf4; Sirpb1a	Osteoclast differentiation
430	FR312018	Osteoclast differentiation	0.011615554	Acp5; Ncf4; Sirpb1a	Osteoclast differentiation
431	FR000826	Osteoclast differentiation	0.011615554	Acp5; Ncf4; Sirpb1a	Osteoclast differentiation
432	FR283504	Tryptophan metabolism	0.011650209	Cyp1a1; Lao1; Tph1	Tryptophan metabolism
433	chr6_86425510_86450160_F	NOD-like receptor signaling pathway	0.011804849	Cxcl1; Naip2	NOD-like receptor signaling pathway
434	chr6_86425510_86450160_F	Legionellosis	0.011804849	Cxcl1; Naip2	Legionellosis
435	chr17_64063362_64066067_R	NOD-like receptor signaling pathway	0.011856089	Cxcl1; Mefv; Naip2	NOD-like receptor signaling pathway
436	chr16_44720170_44723911_R	Fat digestion and absorption	0.011940412	Lipf; Pla2g10	Fat digestion and absorption
437	FR065159	Fat digestion and absorption	0.011940412	Lipf; Pla2g10	Fat digestion and absorption
438	n415160	Lysosome	0.011950867	Acp5; Cd68; Ctsz	Lysosome
439	n283487	Lysosome	0.011950867	Acp5; Cd68; Ctsz	Lysosome
440	FR283503	Lysosome	0.011950867	Acp5; Cd68; Ctsz	Lysosome
441	FR331566	Metabolic pathways	0.011989959	Atp5h; Cox15; Fut2; Gatm; Ggt1; Idh2; Lao1; Lipf; Nme4; Phgdh; Pigx; Pla2g10; Tph1	Metabolic pathways
442	FR150789	Metabolic pathways	0.011989959	Atp5h; Cox15; Gatm; Ggt1; Idh2; Impdh1; Lao1; Lipf; Nme4; Phgdh; Pigx; Pla2g10; Tph1	Metabolic pathways
443	FR233225	Metabolic pathways	0.011989959	Atp5h; Cox15; Cyp1a1; Fut2; Gatm; Ggt1; Idh2; Lao1; Lipf; Nme4; Pigx; Pla2g10; Tph1	Metabolic pathways
444	FR385633	Metabolic pathways	0.011989959	Atp5h; Cox15; Cyp1a1; Fut2; Gatm; Ggt1; Idh2; Impdh1; Lao1; Lipf; Nme4; Pla2g10; Tph1	Metabolic pathways
445	FR162485	Metabolic pathways	0.011989959	Atp5h; Cox15; Cyp1a1; Fut2; Gatm; Ggt1; Idh2; Lao1; Lipf; Nme4; Pigx; Pla2g10; Tph1	Metabolic pathways
446	chr7_26212709_26230775_F	Thyroid hormone synthesis	0.012003213	Lrp2; Tg; Tshr	Thyroid hormone synthesis
447	ENSMUST00000180590	Tryptophan metabolism	0.012070388	Lao1; Tph1	Tryptophan metabolism
448	ENSMUST00000180590	ABC transporters	0.012070388	Abcb8; Abcg3	ABC transporters
449	FR057875	ABC transporters	0.012070388	Abcb8; Abcg3	ABC transporters
450	FR057875	Tryptophan metabolism	0.012070388	Lao1; Tph1	Tryptophan metabolism
451	n415206	Mineral absorption	0.012070388	Ftl1; Slc39a4	Mineral absorption
452	FR044110	TNF signaling pathway	0.012470889	Bcl3; Cx3cl1; Vcam1	TNF signaling pathway

453	FR017535	Metabolic pathways	0.0124712	Atp5h; Cox15; Cyp1a1; Fut2; Gatm; Ggt1; Idh2; Impdh1; Lao1; Lipf; Nme4; Phgdh; Pigx; Pla2g10; Tph1	Metabolic pathways
454	FR283931	Metabolic pathways	0.0124712	Atp5h; Cox15; Cyp1a1; Fut2; Gatm; Ggt1; Idh2; Impdh1; Lao1; Lipf; Nme4; Phgdh; Pigx; Pla2g10; Tph1	Metabolic pathways
455	NR_033764.1	Metabolic pathways	0.0124712	Atp5h; Cox15; Cyp1a1; Fut2; Gatm; Ggt1; Idh2; Impdh1; Lao1; Lipf; Nme4; Phgdh; Pigx; Pla2g10; Tph1	Metabolic pathways
456	chr9_121845920_121858120_F	Metabolic pathways	0.0124712	Atp5h; Cox15; Cyp1a1; Fut2; Gatm; Ggt1; Idh2; Impdh1; Lao1; Lipf; Nme4; Phgdh; Pigx; Pla2g10; Tph1	Metabolic pathways
457	chr13_15947521_15999246_R	Metabolic pathways	0.0124712	Atp5h; Cox15; Cyp1a1; Fut2; Gatm; Ggt1; Idh2; Impdh1; Lao1; Lipf; Nme4; Phgdh; Pigx; Pla2g10; Tph1	Metabolic pathways
458	FR065962	Metabolic pathways	0.0124712	Atp5h; Cox15; Cyp1a1; Fut2; Gatm; Ggt1; Idh2; Impdh1; Lao1; Lipf; Nme4; Phgdh; Pigx; Pla2g10; Tph1	Metabolic pathways
459	chr9_56646768_56649068_R	Metabolic pathways	0.0124712	Atp5h; Cox15; Cyp1a1; Fut2; Gatm; Ggt1; Idh2; Impdh1; Lao1; Lipf; Nme4; Phgdh; Pigx; Pla2g10; Tph1	Metabolic pathways
460	chr15_100908323_100908645_F	Metabolic pathways	0.0124712	Atp5h; Cox15; Cyp1a1; Fut2; Gatm; Ggt1; Idh2; Impdh1; Lao1; Lipf; Nme4; Phgdh; Pigx; Pla2g10; Tph1	Metabolic pathways
461	FR030947	Metabolic pathways	0.0124712	Atp5h; Cox15; Cyp1a1; Fut2; Gatm; Ggt1; Idh2; Impdh1; Lao1; Lipf; Nme4; Phgdh; Pigx; Pla2g10; Tph1	Metabolic pathways
462	n272641	Metabolic pathways	0.0124712	Atp5h; Cox15; Cyp1a1; Fut2; Gatm; Ggt1; Idh2; Impdh1; Lao1; Lipf; Nme4; Phgdh; Pigx; Pla2g10; Tph1	Metabolic pathways
463	FR114000	Fat digestion and absorption	0.012669132	Lipf; Pla2g10	Fat digestion and absorption

464	FR396644	Fat digestion and absorption	0.012669132	Lipf; Pla2g10	Fat digestion and absorption
465	chr14_73729977_73751877_R	Fat digestion and absorption	0.012669132	Lipf; Pla2g10	Fat digestion and absorption
466	chr14_14688472_14747272_F	Fat digestion and absorption	0.012669132	Lipf; Pla2g10	Fat digestion and absorption
467	n294011	Fat digestion and absorption	0.012669132	Lipf; Pla2g10	Fat digestion and absorption
468	chr8_108153471_108159672_F	Fat digestion and absorption	0.012669132	Lipf; Pla2g10	Fat digestion and absorption
469	n415160	Osteoclast differentiation	0.012753717	Acp5; Ncf4; Sirpb1a	Osteoclast differentiation
470	FR202182	Metabolic pathways	0.012841866	Cox15; Cyp1a1; Fut2; Gatm; Ggt1; Idh2; Lao1; Lipf; Nme4; Pigx; Pla2g10; Tph1	Metabolic pathways
471	ENSMUST00000139056	Metabolic pathways	0.012841866	Cox15; Cyp1a1; Fut2; Gatm; Ggt1; Idh2; Lao1; Lipf; Nme4; Pigx; Pla2g10; Tph1	Metabolic pathways
472	FR140001	Metabolic pathways	0.012841866	Atp5h; Cox15; Cyp1a1; Fut2; Gatm; Ggt1; Idh2; Lipf; Nme4; Pigx; Pla2g10; Tph1	Metabolic pathways
473	FR238898	Tryptophan metabolism	0.01288529	Cyp1a1; Lao1; Tph1	Tryptophan metabolism
474	chr10_116814196_116920696_R	Tryptophan metabolism	0.012915348	Lao1; Tph1	Tryptophan metabolism
475	chr10_116814196_116920696_R	ABC transporters	0.012915348	Abcb8; Abcg3	ABC transporters
476	FR388349	Lysosome	0.013078865	Acp5; Cd68; Ctsz	Lysosome
477	FR106089	Lysosome	0.013078865	Acp5; Cd68; Ctsz	Lysosome
478	chr9_27148989_27155714_R	Ether lipid metabolism	0.013112263	Pla2g10; Pla2g7	Ether lipid metabolism
479	chr1_92546371_92551596_R	Ether lipid metabolism	0.013112263	Pla2g10; Pla2g7	Ether lipid metabolism
480	FR394282	Intestinal immune network for IgA production	0.013112263	Icos; Pigr	Intestinal immune network for IgA production
481	FR001084	Glycine, serine and threonine metabolism	0.013283234	Gatm; Phgdh	Glycine, serine and threonine metabolism
482	FR000724	Glycine, serine and threonine metabolism	0.013283234	Gatm; Phgdh	Glycine, serine and threonine metabolism
483	chr4_35106125_35127700_F	Fat digestion and absorption	0.013416882	Lipf; Pla2g10	Fat digestion and absorption
484	n343177	Fat digestion and absorption	0.013416882	Lipf; Pla2g10	Fat digestion and absorption
485	ENSMUST00000130677	NOD-like receptor signaling pathway	0.01344618	Cxcl1; Mefv; Naip2	NOD-like receptor signaling pathway
486	chr2_116796887_116797441_F	Neuroactive ligand-receptor interaction	0.013466307	Grid2; Grik3; P2ry1; Sctr; Tshr	Neuroactive ligand-receptor interaction
487	NR_102339.1	TNF signaling pathway	0.013499916	Bcl3; Cx3cl1; Vcam1	TNF signaling pathway

488	FR283082	Metabolic pathways	0.013616494	Atp5h; Cox15; Fut2; Gatm; Ggt1; Idh2; Impdh1; Lao1; Lipf; Nme4; Phgdh; Pigx; Pla2g10; Tph1	Metabolic pathways
489	chr1_196445304_196445818_R	Metabolic pathways	0.013616494	Atp5h; Cox15; Cyp1a1; Fut2; Gatm; Ggt1; Idh2; Lao1; Lipf; Nme4; Phgdh; Pigx; Pla2g10; Tph1	Metabolic pathways
490	ENSMUST00000150216	Metabolic pathways	0.013616494	Atp5h; Cox15; Cyp1a1; Fut2; Gatm; Ggt1; Idh2; Lao1; Lipf; Nme4; Phgdh; Pigx; Pla2g10; Tph1	Metabolic pathways
491	chr12_89519675_89533975_F	Metabolic pathways	0.013616494	Atp5h; Cox15; Cyp1a1; Fut2; Gatm; Ggt1; Idh2; Lao1; Lipf; Nme4; Phgdh; Pigx; Pla2g10; Tph1	Metabolic pathways
492	chr1_138570107_138632310_R	Metabolic pathways	0.013616494	Atp5h; Cox15; Cyp1a1; Fut2; Gatm; Ggt1; Idh2; Lao1; Lipf; Nme4; Phgdh; Pigx; Pla2g10; Tph1	Metabolic pathways
493	chr8_108153471_108159672_F	Metabolic pathways	0.013625166	Cox15; Cyp1a1; Fut2; Gatm; Ggt1; Lao1; Lipf; Nme4; Pigx; Pla2g10; Tph1	Metabolic pathways
494	chr2_132722530_132726802_F	ABC transporters	0.01378544	Abcb8; Abcg3	ABC transporters
495	chr2_132722530_132726802_F	Tryptophan metabolism	0.01378544	Lao1; Tph1	Tryptophan metabolism
496	FR124060	Tryptophan metabolism	0.01378544	Lao1; Tph1	Tryptophan metabolism
497	FR301567	Intestinal immune network for IgA production	0.013910127	Icos; Pigr	Intestinal immune network for IgA production
498	FR375933	Ether lipid metabolism	0.013910127	Pla2g10; Pla2g7	Ether lipid metabolism
499	chr8_108153471_108159672_F	Intestinal immune network for IgA production	0.013910127	Icos; Pigr	Intestinal immune network for IgA production
500	chr2_11454222_11456321_R	Intestinal immune network for IgA production	0.013910127	Icos; Pigr	Intestinal immune network for IgA production

Table-S6 200 most significant related TF-lncRNA pairs

No	groups	p.value	TransFactor
1	chr9_4260788_4261404_F	0.000232945	Myod1
2	chr2_108456668_108532418_R	0.000382705	Myod1
3	n271825	0.000382705	Myod1
4	chr11_87841800_87858725_F	0.000530277	Myod1
5	FR312018	0.000627705	Pparg
6	FR114000	0.000631573	Myod1
7	chr8_108153471_108159672_F	0.000884981	Myod1
8	n280804	0.00101084	Myod1
9	FR032503	0.001039101	Myod1
10	FR065159	0.001039101	Myod1
11	uc.31+	0.001041428	Myod1
12	n280963	0.001126365	Pparg
13	FR085857	0.001135682	Nr2f1
14	n272641	0.001188641	Myod1
15	FR301567	0.001216696	Myod1
16	FR034857	0.001220911	Myod1
17	FR301809	0.001220911	Myod1
18	ENSMUST00000149707	0.001235286	Pparg
19	chr12_16873410_16873872_F	0.00139461	Myod1
20	chr12_16873410_16873872_F	0.00139461	Myod1
21	FR134333	0.001442956	Myod1
22	FR028133	0.001516808	Nr2f1
23	FR301533	0.001613956	Myod1
24	FR052210	0.001691254	Myod1
25	chr3_21964899_21974524_F	0.001784139	Nr5a1
26	chr13_66381200_66410200_F	0.001788336	Myod1
27	FR120704	0.001861916	Myod1
28	chr7_134372392_134372943_R	0.001925165	Myod1
29	FR374009	0.001925165	Myod1
30	FR065159	0.001953462	Tcf3
31	FR074898	0.001973896	Myod1
32	XR_104983.1	0.002002992	Myod1
33	FR334236	0.002091332	Myod1
34	n344241	0.002091332	Myod1
35	chr7_82902248_82955248_F	0.002215648	Myod1
36	FR087925	0.002225431	Myod1
37	FR140001	0.002225431	Myod1
38	n343177	0.002225431	Myod1
39	chr8_126396600_126407275_R	0.002295215	Myod1
40	FR289473	0.002295215	Myod1
41	chr13_74642700_74701900_F	0.002345082	Myod1
42	FR000826	0.002411501	Pparg
43	chr2_116796887_116797441_F	0.002455415	Myod1
44	FR134333	0.00249149	Tcf3
45	n343233	0.002514735	Pparg
46	FR286933	0.002564112	Myod1
47	chr9_27155685_27156100_F	0.002656004	Nr2f1
48	FR087735	0.002662388	Myod1
49	FR040835	0.002793648	Myod1
50	chr19_23068397_23190256_R	0.002807297	Myod1
51	chr5_31883451_31884082_F	0.002807297	Myod1
52	FR249432	0.002807297	Myod1
53	Gomafu	0.002807297	Myod1
54	FR129526	0.002944933	Myod1
55	n415372	0.002944933	Myod1
56	FR099681	0.003070162	Myod1
57	n416957	0.003070162	Myod1
58	chr1_69531191_69570566_F	0.00316791	Pparg
59	ENSMUST00000139056	0.003176394	Myod1

60	FR202182	0.003176394	Myod1
61	FR000724	0.003176785	Aire
62	chr9_4260788_4261404_F	0.003180795	Tcf3
63	FR114000	0.003183302	Tcf3
64	chr6_129151512_129208737_R	0.003200479	Myod1
65	FR199374	0.003200479	Myod1
66	n263541	0.003285951	Rxra
67	n290758	0.003292255	Rxra
68	chr1_92546371_92551596_R	0.003352549	Nr2f1
69	n291764	0.003352549	Nr2f1
70	chr8_41475940_41476460_F	0.003371833	Myod1
71	FR117799	0.003371833	Myod1
72	FR234548	0.003371833	Myod1
73	FR054246	0.003481462	Pparg
74	chr8_96983590_96994419_F	0.003548114	Pparg
75	n415206	0.00363399	Srf
76	FR266999	0.003638609	Myod1
77	FR392828	0.003667529	Srf
78	chr15_78403377_78412545_F	0.003675691	Myod1
79	FR394282	0.003675691	Myod1
80	chr3_35782698_35789932_R	0.00368153	Rxra
81	FR263105	0.00370956	Pparg
82	FR074898	0.003714769	Tcf3
83	FR395572	0.003848971	Myod1
84	n294791	0.003848971	Myod1
85	NR_045776.1	0.003848971	Myod1
86	ENSMUST00000150216	0.003870866	Myod1
87	FR010306	0.004125523	Myod1
88	chr3_21964899_21974524_F	0.004139359	Nr2f1
89	FR060087	0.00415059	Pparg
90	FR396644	0.004237949	Myod1
91	chr2_71576218_71603818_R	0.004284779	Rxra
92	chr8_108153471_108159672_F	0.004317769	Tcf3
93	chr8_126396600_126407275_R	0.004317769	Tcf3
94	FR238898	0.004319409	Rxra
95	FR394282	0.004327227	Srf
96	n292619	0.004335736	Nr5a1
97	chr17_29374758_29387733_R	0.004380718	Myod1
98	chr9_27148989_27155714_R	0.004403908	Aire
99	n289590	0.004447706	Aire
100	n343177	0.004523539	Tcf3
101	chr8_48476637_48492537_F	0.004617378	Pparg
102	FR066939	0.004630984	Myod1
103	FR115704	0.004630984	Myod1
104	FR142459	0.004673715	Nr2f1
105	FR180118	0.004720482	Aire
106	chr9_121845920_121858120_F	0.004731276	Myod1
107	FR032503	0.004745005	Tcf3
108	FR090778	0.004759119	Efna2
109	FR078628	0.004766833	Rxra
110	FR066939	0.004778574	Nr2f1
111	chr8_48476637_48492537_F	0.004791327	Rxra
112	FR086793	0.004868914	Myod1
113	chr9_27148989_27155714_R	0.004875597	Nr5a1
114	chr10_18036490_18059965_F	0.004906784	Myod1
115	FR215734	0.004906784	Myod1
116	FR003529	0.005052101	Myod1
117	chr7_96640618_96649018_F	0.005138718	Rxra
118	FR301567	0.005199283	Tcf3
119	FR323263	0.005206407	Pparg
120	FR087925	0.00523253	Nr2f1

121	FR142459	0.005244934	Nr5a1
122	FR392828	0.005248093	Pparg
123	FR396644	0.00527982	Rxra
124	chr1_183783306_183834531_F	0.00540201	Myod1
125	FR003529	0.005429193	Rxra
126	FR283504	0.005433514	Rxra
127	chr9_56646768_56649068_R	0.005504941	Myod1
128	FR030947	0.005504941	Myod1
129	FR273612	0.005538306	Esr1
130	FR149067	0.005574606	Myod1
131	chr11_6796679_6797670_F	0.005626567	Myod1
132	chr9_66976750_67014625_F	0.005704064	Myod1
133	FR279378	0.005704064	Myod1
134	FR388349	0.0057112	Arhgef7
135	FR388349	0.0057112	Cd40
136	n283487	0.0057112	Arhgef7
137	n283487	0.0057112	Cd40
138	FR099681	0.005766868	Tcf3
139	FR301809	0.005766868	Tcf3
140	FR398266	0.005901773	Nr5a1
141	chr1_162965001_162966376_F	0.005920099	Myod1
142	chr12_110003268_110030953_F	0.005920099	Myod1
143	chr6_129151512_129208737_R	0.005960498	Pparg
144	chr11_120079199_120130099_R	0.00598428	Rxra
145	chr18_69923410_69965360_R	0.00598428	Rxra
146	FR042390	0.005995436	Myod1
147	FR208702	0.005995436	Myod1
148	n415206	0.00610045	Myod1
149	chr15_38205154_38206196_F	0.006161875	Myod1
150	FR064953	0.006216656	Runx3
151	n283487	0.006307416	Esr1
152	FR035623	0.006350434	Myod1
153	FR285140	0.006350434	Myod1
154	FR285140	0.006350434	Myod1
155	chr6_47959825_47960502_R	0.006356264	Myod1
156	chr12_55271691_55276492_R	0.006361298	Myod1
157	chr5_35893908_35896613_R	0.006379325	Rxra
158	n297308	0.006411243	Aire
159	FR388349	0.006445616	Nr2f1
160	n414277	0.006445616	Nr2f1
161	n415206	0.006604408	Pparg
162	FR167447	0.006635473	Esr1
163	ENSMUST00000132130	0.006644166	Myod1
164	FR296035	0.006644166	Myod1
165	FR365932	0.006644166	Myod1
166	FR365932	0.006679862	Nr2f1
167	chr2_35828611_35838083_F	0.006713985	Myod1
168	chr5_54053882_54054174_F	0.006749165	Esr1
169	chr7_91558475_91733625_R	0.00679122	Myod1
170	FR325017	0.006881757	Myod1
171	FR283082	0.006938234	Rxra
172	FR203009	0.007061258	Pparg
173	chr18_69923410_69965360_R	0.007068479	Myod1
174	n283487	0.007071847	Nr1h2
175	chrX_102568332_102587093_R	0.007148422	Myod1
176	chrX_18734106_18744367_R	0.007148422	Myod1
177	FR092975	0.007148422	Myod1
178	FR389545	0.007148422	Myod1
179	chr7_31709550_31723025_F	0.0072095	Myod1
180	ENSMUST00000141575	0.007289777	Rxra
181	FR269524	0.007295925	Nr2f1

182	FR066939	0.007328014	Nr5a1
183	FR052210	0.007358646	Tcf3
184	FR396644	0.007358646	Tcf3
185	chr9_27148989_27155714_R	0.007388519	Casr
186	chr9_27148989_27155714_R	0.007388519	Cxadr
187	chr9_27148989_27155714_R	0.007388519	Nr1i3
188	chr9_27148989_27155714_R	0.007388519	Prkar1a
189	chr9_27148989_27155714_R	0.007388519	Spg7
190	chr9_27148989_27155714_R	0.007388519	Trim13
191	chr11_120079199_120130099_R	0.007461167	Nr2f1
192	FR388349	0.00746851	Runx3
193	chr7_133402766_133415516_R	0.007478924	Rxra
194	FR065962	0.007478924	Rxra
195	FR312820	0.007668905	Myod1
196	chr13_98038568_98068079_F	0.00767049	Myod1
197	FR097477	0.00767049	Myod1
198	FR139436	0.00767049	Myod1
199	FR139436	0.00767049	Myod1
200	FR155040	0.00767049	Myod1
